# Integrative single-cell transcriptomics and proteomics reveal an immunometabolic framework for MSC-exosome-mediated remodeling of expanded NK cells

**DOI:** 10.1093/gigascience/giag049

**Published:** 2026-04-20

**Authors:** Yunyun Fu, Yi Liu, Mingwen Xu, Gaojun Liu, Jianzhi Sun, Fanyu Bu, Wenqing Xie, Jiayi Zhao, Jun Luo, Qiang Guo, Yinghua Huang, Fengping Xu, Siqi Liu, Longqi Liu, Ying Fu, Xuan Dong

**Affiliations:** College of Life Sciences, University of Chinese Academy of Sciences, 19A Yuquan Road, Shijingshan District, Beijing 100049, China; State Key Laboratory of Genome and Multi-omics Technologies, BGI Research, Hangzhou; Key Laboratory of Spatial Omics of Zhejiang Province, BGI Research, 203 Zhenzhong Road, Xihu District, Hangzhou 310030, China; College of Life Sciences, University of Chinese Academy of Sciences, 19A Yuquan Road, Shijingshan District, Beijing 100049, China; State Key Laboratory of Genome and Multi-omics Technologies, BGI Research, Hangzhou; Key Laboratory of Spatial Omics of Zhejiang Province, BGI Research, 203 Zhenzhong Road, Xihu District, Hangzhou 310030, China; State Key Laboratory of Genome and Multi-omics Technologies, BGI Research, Hangzhou; Key Laboratory of Spatial Omics of Zhejiang Province, BGI Research, 203 Zhenzhong Road, Xihu District, Hangzhou 310030, China; Key Laboratory of Systems Health Science of Zhejiang Province, School of Life Science, Hangzhou Institute for Advanced Study, University of Chinese Academy of Sciences, 1 Sub-Lane Xiangshan, Xihu District, Hangzhou 310024, China; College of Life Sciences, University of Chinese Academy of Sciences, 19A Yuquan Road, Shijingshan District, Beijing 100049, China; State Key Laboratory of Genome and Multi-omics Technologies, BGI Research, Hangzhou; Key Laboratory of Spatial Omics of Zhejiang Province, BGI Research, 203 Zhenzhong Road, Xihu District, Hangzhou 310030, China; College of Life Sciences, University of Chinese Academy of Sciences, 19A Yuquan Road, Shijingshan District, Beijing 100049, China; State Key Laboratory of Genome and Multi-omics Technologies, BGI Research, Hangzhou; Key Laboratory of Spatial Omics of Zhejiang Province, BGI Research, 203 Zhenzhong Road, Xihu District, Hangzhou 310030, China; State Key Laboratory of Genome and Multi-omics Technologies, BGI Research, Hangzhou; Key Laboratory of Spatial Omics of Zhejiang Province, BGI Research, 203 Zhenzhong Road, Xihu District, Hangzhou 310030, China; Interdisciplinary Research Center on Biology and Chemistry, Shanghai Institute of Organic Chemistry, Chinese Academy of Sciences, 384A Bibo Road, Pudong New Area, Shanghai 201210, China; School of Life Science and Technology, China Pharmaceutical University, 639 Longmian Avenue, Jiangning District, Nanjing 211198, China; College of Life Sciences, University of Chinese Academy of Sciences, 19A Yuquan Road, Shijingshan District, Beijing 100049, China; HIM-BGI Omics Center, Hangzhou Institute of Medicine (HIM), Chinese Academy of Sciences, 150 Dongfang Street, Xiasha Subdistrict, Hangzhou 310018, China; State Key Laboratory of Genome and Multi-omics Technologies, BGI Research, Hangzhou; Key Laboratory of Spatial Omics of Zhejiang Province, BGI Research, 203 Zhenzhong Road, Xihu District, Hangzhou 310030, China; BGI Cell, 203 Zhenzhong Road, Xihu District, Hangzhou 310030, China; College of Life Sciences, University of Chinese Academy of Sciences, 19A Yuquan Road, Shijingshan District, Beijing 100049, China; BGI Cell, 146 BeishanRoad, Yantian Street, Yantian District, Shenzhen 518083, China; HIM-BGI Omics Center, Hangzhou Institute of Medicine (HIM), Chinese Academy of Sciences, 150 Dongfang Street, Xiasha Subdistrict, Hangzhou 310018, China; State Key Laboratory of Genome and Multi-omics Technologies, BGI Research, Hangzhou; Key Laboratory of Spatial Omics of Zhejiang Province, BGI Research, 203 Zhenzhong Road, Xihu District, Hangzhou 310030, China; State Key Laboratory of Genome and Multi-omics Technologies, BGI Research, Hangzhou; Key Laboratory of Spatial Omics of Zhejiang Province, BGI Research, 203 Zhenzhong Road, Xihu District, Hangzhou 310030, China; State Key Laboratory of Genome and Multi-omics Technologies, BGI Research, Hangzhou; Key Laboratory of Spatial Omics of Zhejiang Province, BGI Research, 203 Zhenzhong Road, Xihu District, Hangzhou 310030, China; Guangdong Provincial Key Laboratory of Human Disease Genomics, BGI Research, 9 Yunhua Road, Meisha Street, Yantian District, Shenzhen 518083, China

**Keywords:** mesenchymal stem cell-derived exosomes, natural killer cells, single-cell transcriptomics, effector-cytotoxic state, immunometabolic framework

## Abstract

**Background:**

Natural killer (NK) cells play a central role in anti-tumor immunity and immunosurveillance of senescence, yet their clinical performance is frequently limited by functional exhaustion during *ex vivo* expansion. Mesenchymal stem cell-derived exosomes (MSC-Exos) are increasingly recognized as immunomodulators, but their broader effects on NK cell fitness and functional states remain incompletely characterized.

**Results:**

Here, we assessed MSC-Exos-mediated regulation of human NK cells using a standardized *ex vivo* priming platform integrated with single-cell transcriptomics and proteomic profiling. MSC-Exos significantly improved NK cell viability in a dose- and time-dependent manner while preserving a CD56⁺CD3^−^ NK cell-enriched phenotype. MSC-Exos-treated NK cells showed enhanced cytotoxicity against K562 tumor cells and senescent fibroblasts. This phenotype was accompanied by increased expression of the activating receptors NKG2D and CD16, reduced LAG3 expression, and enhanced granzyme B expression and degranulation. Consistent with improved NK cell fitness, MSC-Exos treatment was also associated with upregulated expression of genes involved in NRF2-linked redox programs and improved mitochondrial readouts in NK cells. Single-cell analyses of MSC-Exos-treated NK cells revealed enhanced immune-effector programs and reduced inflammatory stress, while trajectory inference indicated that MSC-Exos may bias the NK cell state distribution toward more cytotoxic effector-like states. Proteomic profiling of MSC-Exos identified enrichment of FcγR-associated signaling components, supporting the hypothesis that exosomal composition may be related to the FcγR/CD16-associated transcriptional and phenotypic features observed in MSC-Exos-treated NK cells.

**Conclusions:**

Our data indicate that MSC-Exos improve NK cell viability and functional fitness during *ex vivo* expansion and bias NK cells toward a more effector-cytotoxic state. Together, these findings provide an immunometabolic framework for MSC-Exos-assisted NK cell manufacturing while underscoring the need for further causal validation.

## Background

The global population is aging rapidly, intensifying the burden of immunosenescence and increasing susceptibility to infections, malignancies, and chronic degenerative diseases. This trend underscores the need for safe, effective, and scalable immune interventions. As key components of innate immunity, natural killer (NK) cells eliminate infected or malignant cells in a non-major histocompatibility complex (non-MHC)-restricted manner and contribute to both antitumor immunity and clearance of senescent cells [1–4]. However, clinical translation typically requires *ex vivo* expansion, which often induces functional attenuation, metabolic exhaustion, and phenotypic instability, thereby limiting efficacy in adoptive cell therapy [5, 6].

Mesenchymal stem cell-derived exosomes (MSC-Exos) are nanoscale extracellular vesicles secreted by mesenchymal stem cells (MSCs). They mediate intercellular communication by delivering diverse bioactive cargo, including proteins, lipids, and nucleic acids. With their low immunogenicity and high biocompatibility, MSC-Exos have emerged as important regulators of tissue repair, immune modulation, and tumor microenvironment remodeling [7, 8].

Notably, the immunomodulatory effects of MSC-Exos are context-dependent rather than fixed. Although MSC-derived signals are generally associated with immunosuppressive and anti-inflammatory effects in graft-versus-host disease (GvHD) and systemic inflammation models, MSCs can exert divergent effects on T-cell proliferation depending on cell ratios and culture conditions [9–12], indicating marked functional plasticity of MSC-derived regulatory cues. This plasticity suggests that MSC-Exos may not be limited to immune suppression, but may also support effector immune-cell function under defined conditions. In parallel, MSC-Exos have been implicated in immune regulation through effects on regulatory T-cell differentiation [13], macrophage polarization [14]. Moreover, engineered extracellular vesicles can modulate WNT signalling to promote tissue repair and regeneration, suggesting that MSC-Exos may also be further engineered to enhance their bioactivity and therapeutic potential [15, 16].

Recently, the potential of MSC-Exos to enhance innate immune cell function under specific conditions has garnered increasing attention. For instance, bone marrow-derived MSC-Exos can augment NK cell cytotoxicity against hepatocellular carcinoma cells [17], and a positive feedback regulatory loop between MSCs and NK cells may improve impaired NK cell function in severe pathological states [18]. Nevertheless, systematic investigations into how MSC-Exos influence NK cell viability, phenotypic state, and function during *ex vivo* expansion, as well as the underlying molecular coordination, remain limited.

Single-cell approaches are well suited to resolve hierarchical immune substructures and state transitions within heterogeneous populations, providing a high-resolution context for interpreting multi-omic changes [19, 20]. In this study, we established a standardized *ex vivo* workflow to investigate how MSC-Exos modulate expanding human peripheral blood-derived NK cells. Specifically, we examined their effects on cell viability, functional state, and cytotoxic programs, while assessing the preservation of core lineage features. By integrating flow cytometry, functional assays, single-cell transcriptomics, and proteomics, we aimed to define an immunometabolic framework for MSC-Exos-mediated NK cell remodeling and to provide a rationale for MSC-Exos-augmented NK cell immunotherapy.

## Results

### MSC-Exos improve NK cell viability during *ex vivo* expansion while preserving an NK cell-enriched phenotype

**Figure 1:**
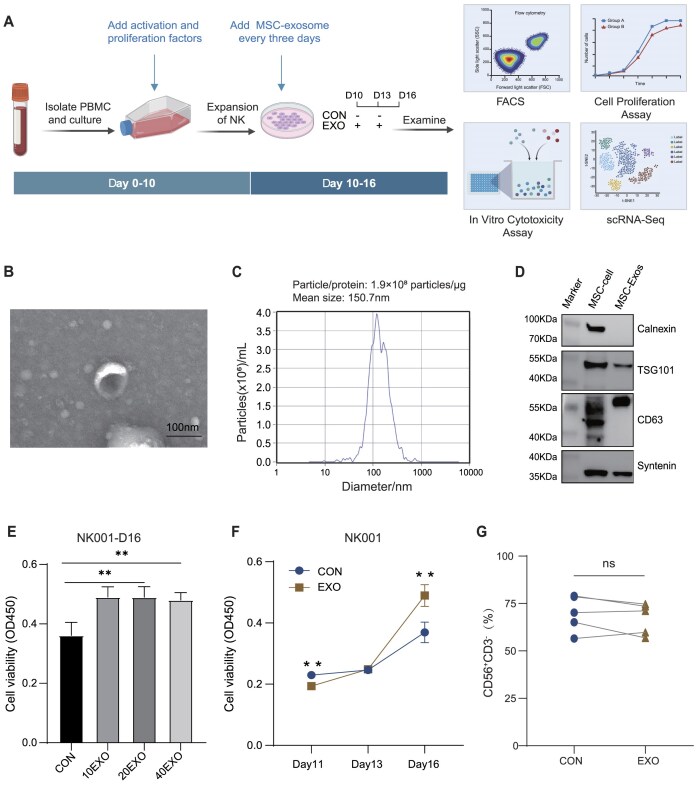
MSC-Exos improve NK cell viability during *ex vivo* expansion while preserving an NK cell-enriched phenotype. (A) Experimental design and workflow. PBMCs were cultured for *ex vivo* NK cell expansion from day 0 to day 10. From day 10 to day 16, the EXO group received MSC-Exos every 3 days, whereas the CON group was maintained without exosome supplementation. Endpoint analyses included flow cytometry, CCK-8-based viability assessment, *in vitro* cytotoxicity assays, and scRNA-seq. (B) Representative TEM image showing the morphology of MSC-Exos (scale bar, 100 nm). (C) NTA showing the size distribution profile of MSC-Exos. (D) Immunoblotting analysis of MSC-Exos showing enrichment of exosomal markers (CD63, Syntenin, and TSG101) and absence of the endoplasmic reticulum marker Calnexin. (E) Quantification of NK cell viability in donor NK001 by CCK-8 assay (OD450) following treatment with MSC-Exos at 10–40 μg/mL. (F) Quantification of NK cell viability in donor NK001 by CCK-8 assay (OD450) following treatment with 20 μg/mL MSC-Exos during the late expansion phase, with measurements collected on days 11, 13, and 16. (G) Flow cytometry analysis showing comparable frequencies of CD56⁺CD3^−^ NK cells between the CON and EXO groups. Statistics: For panels E and F, data are presented as mean ± SD from *n* = 4 technical replicates, and significance was evaluated using an unpaired two-tailed Student’s *t*-test. For panel G, *n* = 5 biological replicates (donors) were analyzed using a paired two-tailed Student’s *t*-test (paired samples are connected by lines). ns, not significant; **P < 0.01. Figure 1A partially use templates and icons from BioRender (BioRender.com), with permission for publication. These figures were prepared under a paid academic subscription plan.

To evaluate the effects of MSC-Exos on NK cell viability and functional state during *ex vivo* expansion, we established an expansion-and-intervention workflow with multidimensional readouts (Fig. 1A). Briefly, peripheral blood mononuclear cells (PBMCs) from healthy donors were cultured and expanded for 10 days using a commercially available, standardized culture system to generate NK cells. Starting on day 10, NK cells in the MSC-Exos-treated group (EXO) received MSC-Exos every three days, whereas those in the control group (CON) received no exosome supplementation. On day 16, NK cells from both the CON and EXO groups were subjected to flow cytometry, functional assays, and single-cell RNA sequencing (scRNA-seq).

Prior to subsequent intervention experiments, the isolated MSC-Exos were systematically characterized with respect to their morphology, size distribution, and marker protein expression. Vesicles isolated from MSC-conditioned medium by size-exclusion chromatography (SEC) exhibited typical exosome-like morphology under transmission electron microscopy (TEM) (Fig. 1B). These vesicles were further characterized by nanoparticle tracking analysis (NTA), which revealed a relatively homogeneous particle population with a mean diameter of 150.7 nm; most particles ranged from 60 to 200 nm, with a particle-to-protein ratio of 1.9 × 10^8^ particles/µg (Fig. 1C). Immunoblotting analysis of these vesicles confirmed enrichment of established exosomal markers (CD63, TSG101, and Syntenin) and the absence of the endoplasmic reticulum contaminant Calnexin (Fig. 1D), supporting their exosomal identity and purity in accordance with the Minimal Information for Studies of Extracellular Vesicles (MISEV) guidelines. To facilitate cross-preparation standardization, MSC-Exos input was quantified using protein-, particle-, and cell-normalized dosing metrics (Supplementary Table S1). Specifically, a dose of 20 μg/mL corresponded to 3.8 × 10^9^ particles/mL based on the measured particle-to-protein ratio of 1.9 × 10^8^ particles/μg. Under the culture condition used here (1.5 × 10^6^ cells/mL), this corresponded to an estimated exposure of 2,530 particles per cell, with a protein-to-particle ratio of 52.63 μg per 1 × 10^10^ particles.

We next evaluated whether MSC-Exos improve NK cell viability under standard expansion conditions by comparing NK cells from multiple donors in the CON and EXO groups, with the EXO group tested across graded MSC-Exos doses of 10, 20, and 40 μg/mL (Supplementary Tables S1 and S2). After 6 days of MSC-Exos treatment, corresponding to day 16 of culture, NK cell viability was significantly enhanced in a dose-dependent manner, as measured by the cell counting kit-8 (CCK-8) assay with optical density (OD) read at 450 nm, with the greatest effect observed at 20 μg/mL (Fig. 1E; Supplementary Fig. S1A). Longitudinal analysis further showed that this pro-viability effect increased over time and was most pronounced on day 16 (Fig. 1F and Supplementary Fig. S1B and S1C).

Flow cytometry indicated that both groups maintained a high frequency of CD56⁺CD3^−^ cells (~56.5–79.0%), with no significant differences between CON and EXO (Fig. 1G and Supplementary Fig. S1D). Thus, MSC-Exos improve NK cell viability during *ex vivo* expansion without compromising NK-lineage identity, providing a foundation for subsequent analyses of functional potentiation and mechanism.

### MSC-Exos enhance NK cell cytotoxicity with increased activating-receptor expression and improved mitochondrial fitness

**Figure 2:**
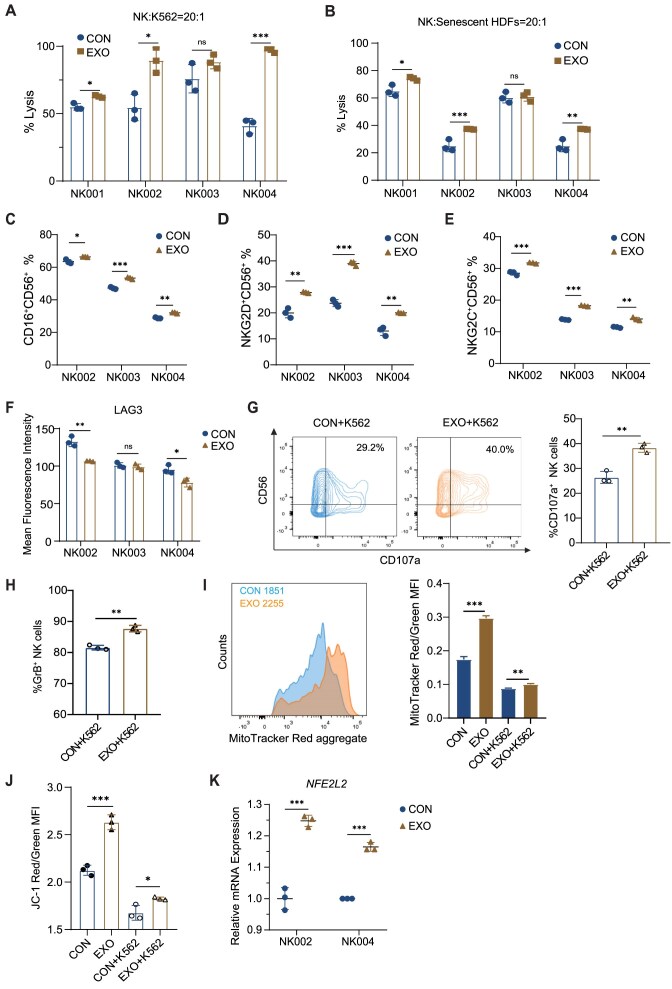
MSC-Exos are associated with enhanced NK cell cytotoxicity, activating receptor expression, degranulation, and mitochondrial fitness-related readouts. (A) Percentage lysis of K562 tumor cells by NK cells from different donors in the CON and EXO groups. The effector-to-target (E:T) ratio was 20:1. (B) Percentage lysis of DOX-induced senescent HDFs by NK cells from different donors in the CON and EXO groups. The E:T ratio was 20:1. (C–E) Flow cytometric analysis of the percentages of NK cells expressing the activating receptors CD16 (C), NKG2D (D), and NKG2C (E) from different donors in the CON and EXO groups. (F) Mean fluorescence intensity (MFI) of the inhibitory receptor LAG3 on NK cells from different donors in the CON and EXO groups. (G) Degranulation of NK cells, measured as the percentage of CD107a⁺ cells after stimulation with K562 target cells. (H) Granzyme B expression in NK cells, measured as the percentage of GrB⁺ cells after stimulation with K562 target cells. (I) Mitochondrial staining-based readout in NK cells assessed by MitoTracker Red/Green under basal conditions and after stimulation with K562 cells. (J) Mitochondrial membrane potential (ΔΨm) in NK cells, expressed as the JC-1 red/green fluorescence intensity ratio under basal conditions and after stimulation with K562 cells. (K) Relative mRNA expression of *NFE2L2* (NRF2), a key antioxidant regulator, in NK cells from different donors, as determined by qRT-PCR. Statistics: For panels A–K, data are presented as mean ± SD from *n* = 3 technical replicates, and significance was evaluated using an unpaired two-tailed Student’s *t*-test. ns, not significant; *P < 0.05, **P < 0.01, ***P < 0.001.

Having established that MSC-Exos improve NK cell viability during *ex vivo* expansion while maintaining a CD56⁺CD3⁻ NK cell-enriched phenotype, we next examined NK cell effector function. MSC-Exos pretreatment markedly increased NK cell-mediated lysis of K562 tumor cells (Fig. 2A and Supplementary Fig. S2D). To model a senescence-relevant *in vitro* context, we induced senescence in human dermal fibroblasts (HDFs) using doxorubicin (DOX). DOX exposure for 48 h reduced HDF viability in a dose-dependent manner (Supplementary Fig. S2A). Treatment with 150 nM DOX for 48 h robustly induced hallmark senescent features in HDFs, characterized by an enlarged, flattened morphology, a significant increase in senescence-associated β-galactosidase (SA-β-Gal) positivity (Supplementary Fig. S2B), and transcriptional upregulation of senescence- and inflammation-associated markers, including *TNF, CDKN1A*, and *IL1B* (Supplementary Fig. S2C). In cytotoxicity assays against senescent HDFs, MSC-Exos significantly enhanced NK cell killing in multiple donors (NK001, NK002, and NK004), whereas donor NK003 showed a non-significant trend (Fig. 2B and Supplementary Fig. S2E), indicating inter-donor variability in response to exosomal priming.

To identify phenotypic correlates of enhanced cytotoxicity, we profiled NK cell receptor expression. Compared with controls, NK cells in the EXO group exhibited increased expression of the activating receptors NKG2D, CD16, and NKG2C, together with reduced mean fluorescence intensity (MFI) of the inhibitory checkpoint LAG3 (Fig. 2C–F). At the effector level, MSC-Exos-treated NK cells displayed stronger degranulation responses, with higher CD107a mobilization and increased granzyme B expression upon target stimulation (Fig. 2G and H).

Given that sustained cytotoxic function depends on adequate metabolic support [21, 22], we next examined whether MSC-Exos also improved mitochondrial fitness in NK cells. NK cells in the EXO group showed an increased MitoTracker Red/Green ratio and elevated mitochondrial membrane potential (ΔΨm; JC-1 ratio), and these advantages were maintained after exposure to target tumor cells (Fig. 2I and J). Consistently, quantitative real-time polymerase chain reaction (qRT-PCR) showed higher NRF2 (*NFE2L2*) mRNA levels in MSC-Exos-treated NK cells (Fig. 2K), consistent with the engagement of NRF2-associated antioxidant programs that may help sustain mitochondrial homeostasis during repeated cytotoxic challenges [22].

Together, these findings support an association between MSC-Exos treatment and enhanced NK cell cytotoxicity against both tumor and senescent targets, accompanied by increased activating receptor expression, reduced inhibitory features, and improved mitochondrial fitness linked to redox-supportive programs. Collectively, these changes correspond to greater cytotoxic efficiency across multiple complementary functional readouts.

### MSC-Exos promote cytotoxic transcriptional programs while reducing stress-related signatures in NK cells

**Figure 3:**
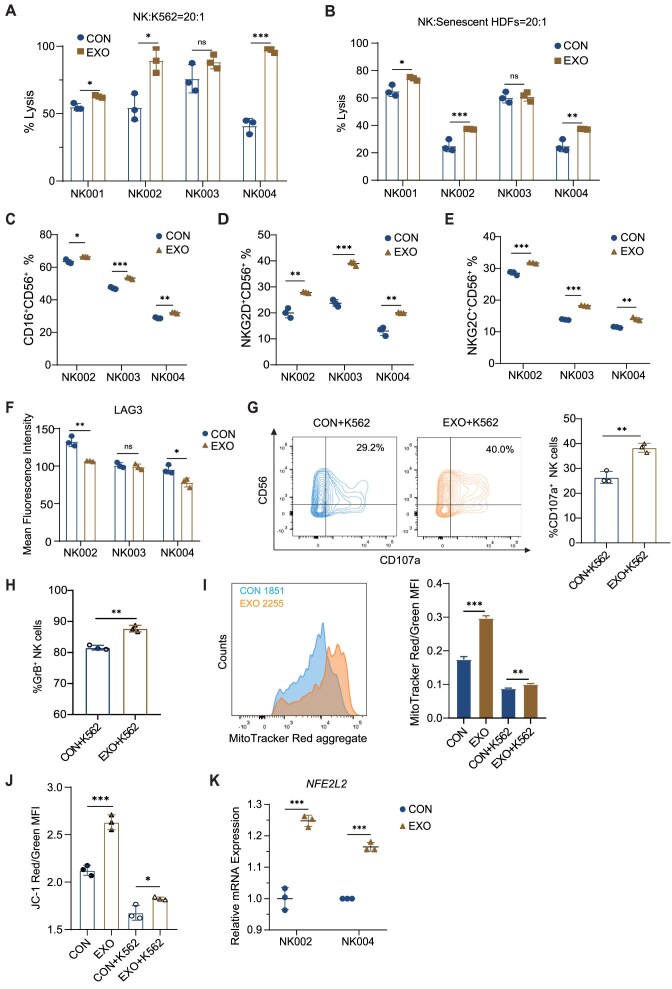
Transcriptomic profiling reveals immunometabolic alterations associated with MSC-Exos treatment in NK cells. (A) Experimental workflow for scRNA-seq of NK cells cultured with or without MSC-Exos. PBMCs from four independent healthy donors (NK001–NK004) were expanded for 16 days, with MSC-Exos added every 3 days during the final culture phase (days 10–16). Single-cell libraries were generated from each donor for group-level differential expression and subcluster analyses. (B) Heatmap showing the mean expression of cytotoxicity-associated genes (*FCGR3A, GZMB, NKG7, GNLY, IFNG*, and *TNF*) across the CON and EXO groups. Gene expression is scaled by *z*-score.(C) Violin plots showing cytotoxic granule signature scores. (D) Violin plots showing signature scores for cellular senescence, SASP, and oxidative stress responses. (E) Volcano plot showing DEGs between EXO and CON groups, with selected genes of interest highlighted. (F) GO enrichment analysis of genes upregulated in the EXO group, highlighting biological processes related to cytoplasmic translation, immune activation, and cytokine responses. (G) GSEA showing enrichment of Fcγ receptor-dependent phagocytosis, Rho GTPase signaling, and glycolysis pathways in the EXO group (FDR *q* < 0.25). Statistics: For panels C and D, significance was assessed using a two-sided Mann–Whitney *U* test, ***P < 0.001. For panel E, differential expression was evaluated using a two-sided Wilcoxon rank-sum test. Figure 3A partially use templates and icons from BioRender (BioRender.com), with permission for publication. These figures were prepared under a paid academic subscription plan.

To characterize transcriptional programs underlying MSC-Exos-mediated remodeling, we performed scRNA-seq on NK cells from CON and EXO groups followed by group-level differential expression (DE) analysis and subcluster annotation (Fig. 3A). To minimize lineage contamination and transcriptional ambiguity, major immune lineages were annotated using canonical marker sets, and non-NK-lineage cells were removed prior to constructing the NK cell-only dataset for downstream analyses. Clusters lacking defining NK cell markers and exhibiting poor marker specificity were further excluded to ensure high-quality lineage-restricted analyses (Supplementary Table S4). A total of 28,382 NK cells were retained for downstream analyses. Compared with controls, NK cells in the EXO group showed higher expression of cytotoxic effector genes, including *GZMB, FCGR3A, NKG7, CTSW*, and *GNLY*, whereas pro-inflammatory cytokine transcripts such as *IFNG* and *TNF* were reduced (Fig. 3B). Concomitantly, the EXO group exhibited lower IFN-γ protein levels (Supplementary Fig. S3A) alongside higher cytotoxic granule scores (Fig. 3C), consistent with features of a more cytotoxic phenotype. In addition, NK cells in the EXO group showed attenuated expression of genes associated with the senescence-associated secretory phenotype (SASP), cellular senescence, and oxidative stress responses, suggesting a reduced cellular stress burden and improved functional competence (Fig. 3D).

DE analysis further showed that NK cells in the EXO group upregulated activation-associated genes, including *EGR1, ISG20, JUNB*, and *MYC* (Fig. 3E). Consistent with these transcriptional changes, pathway analyses showed that NK cells in the EXO group were enriched for cytoplasmic translation and type I interferon signaling pathways (Fig. 3F), in line with enhanced biosynthetic capacity and immune activation. In this context, enrichment of type I interferon signaling likely reflects antiviral and activation-associated transcriptional circuitry rather than a direct increase in IFN-γ output. Gene set enrichment analysis (GSEA) further showed enrichment of FcγR-related processes, Rho GTPase-IQGAP signaling, and glycolytic pathways in the EXO group (Fig. 3G), suggesting coordinated cytoskeletal and metabolic remodeling associated with enhanced NK cell effector function. This interpretation was further supported by metabolic features of NK cells in the EXO group, including increased oxidative phosphorylation and pentose phosphate pathway activity, whereas decreased pyruvate and ketone body metabolism suggested altered carbon substrate utilization (Supplementary Fig. S3B and C). These transcriptomic shifts were concordant with the improved mitochondrial fitness and increased NRF2 (*NFE2L2*) expression observed in EXO-group NK cells (Fig. 2I–K), together indicating enhanced energy management and redox buffering that may help restrain inflammatory stress while maintaining functional stability.

### MSC-Exos are associated with a shift in NK cell states toward effector-cytotoxic phenotypes at the single-cell level

**Figure 4:**
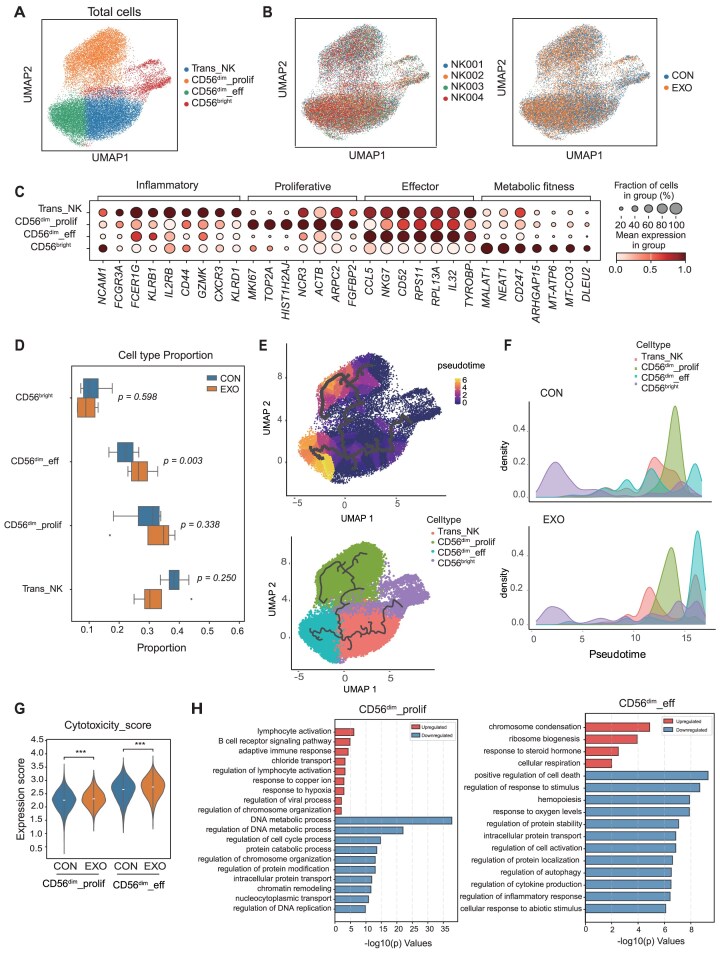
Single-cell analyses suggest that MSC-Exos bias NK cell subsets toward effector-cytotoxic states. (A) UMAP plot showing four major NK cell subsets: Trans_NK, CD56^dim^_prolif, CD56^dim^_eff, and CD56^bright^. (B) UMAP plots showing donor distribution (left) and treatment grouping (right), indicating comparable representation of subclusters across four donors and treatment conditions (CON vs. EXO). (C) Dot plot showing representative marker genes for inflammatory, proliferative, effector, and metabolic fitness signatures across NK cell subclusters, with dot size indicating fraction of cells per group and color indicating mean expression. (D) Box plot of NK cell subset proportions between CON and EXO groups. (E) Pseudotime analysis illustrating differentiation trajectories of NK cell subpopulations, with black lines representing predicted lineage paths. (F) Density distribution of pseudotime states across subclusters in CON and EXO groups.(G) Violin plots showing cytotoxicity expression scores of CD56^dim^_prolif and CD56^dim^_eff subsets between CON and EXO groups. (H) GO enrichment analysis of differentially expressed genes in CD56^dim^_prolif and CD56^dim^_eff subsets. Statistics: For panel D, significance was assessed using a paired two-tailed Student’s *t*-test. For panel G, significance was assessed using a two-sided Mann–Whitney *U* test, ***P < 0.001.

Given that MSC-Exos substantially reshaped the transcriptional programs of the expanded NK cell products, we hypothesized that MSC-Exos might also modulate NK cell differentiation states at the single-cell level. To test this, we performed unsupervised clustering of the integrated scRNA-seq dataset to resolve NK cell subpopulations and to determine whether MSC-Exos bias the population toward specific functional states. Unsupervised clustering identified four NK cell subclusters (Fig. 4A and C): (i) CD56^bright^, a regulatory-leaning subset with enhanced metabolic fitness features, characterized by high expression of *MALAT1, NEAT1, CD247*, and *MT-ATP6*; (ii) Trans_NK, a transitional or inflammatory subset enriched for *FCER1G, KLRB1, IL2RB*, and *CD44*; (iii) CD56^dim^_prolif, a proliferative subset marked by cell-cycle-associated genes, including *MKI67, TOP2A*, and *HIST1H2AC*; and (iv) CD56^dim^_eff, an effector-cytotoxic subset with elevated expression of *NKG7, CD52*, and *IL32*. These subclusters were consistently detected across donors and treatment conditions (Fig. 4B), supporting robust integration and effective batch correction. This was further supported by UMAP visualizations before and after Harmony correction, which showed improved donor mixing while preserving the overall NK cell subcluster architecture under both CON and EXO conditions (Supplementary Fig. S4A–C).

MSC-Exos treatment significantly increased the proportion of CD56^dim^_eff cells (*P =* 0.003), whereas the fraction of CD56^bright^ cells remained largely unchanged (Fig. 4D). Donor-stratified analyses further indicated that the EXO-associated enrichment of CD56^dim^_eff cells was not driven by a single donor (Supplementary Fig. S4D and Table S5). This shift toward the effector-cytotoxic compartment provides a cellular basis for the enhanced cytotoxic function observed in EXO-group NK cell products.

To examine whether MSC-Exos influence NK cell maturation dynamics, we reconstructed differentiation trajectories using pseudotime analysis (Fig. 4E and F). Trajectory inference suggested a shift from CD56^bright^ to CD56^dim^_eff states, with the EXO group showing a higher density of later effector-like cells along pseudotime. Supporting this observation, NK cells in the EXO group exhibited significantly increased cytotoxicity scores in both the CD56^dim^_prolif and CD56^dim^_eff subsets (Fig. 4G). Gene ontology (GO) enrichment analysis of subset-specific differentially expressed genes (DEGs) further revealed upregulation of pathways associated with lymphocyte activation, ribosome biogenesis, and cellular respiration in NK cells from the EXO group (Fig. 4H), supporting enhanced effector function and metabolic activity in these NK cell subsets. These findings suggest that MSC-Exos favor functional maturation of the CD56^dim^ NK cell compartment toward a more metabolically active and cytotoxic state.

Collectively, these results indicate that MSC-Exos remodel NK cell differentiation trajectories while preserving NK-lineage identity, thereby enhancing the balance between NK cell fitness and cytotoxic function.

### Proteomic profiling identifies FcγR-associated signaling components in MSC-Exos and supports a hypothesis of FcγR/CD16-related priming

To explore the molecular features of MSC-Exos that may relate to NK cell activation programs [23], we performed quantitative proteomic profiling of MSC-Exos. In total, 1,098 proteins were identified in MSC-Exos (Supplementary Fig. S5A; Supplementary Tables S6–S8). Most identified peptides ranged from 8 to 20 amino acids in length (Supplementary Fig. S5B). Notably, 83.88% of proteins were supported by two or more peptides (Supplementary Fig. S5C), and 69% of proteins showed sequence coverage >10% (Supplementary Fig. S5D), indicating high confidence in MSC-Exos protein identification.

**Figure 5:**
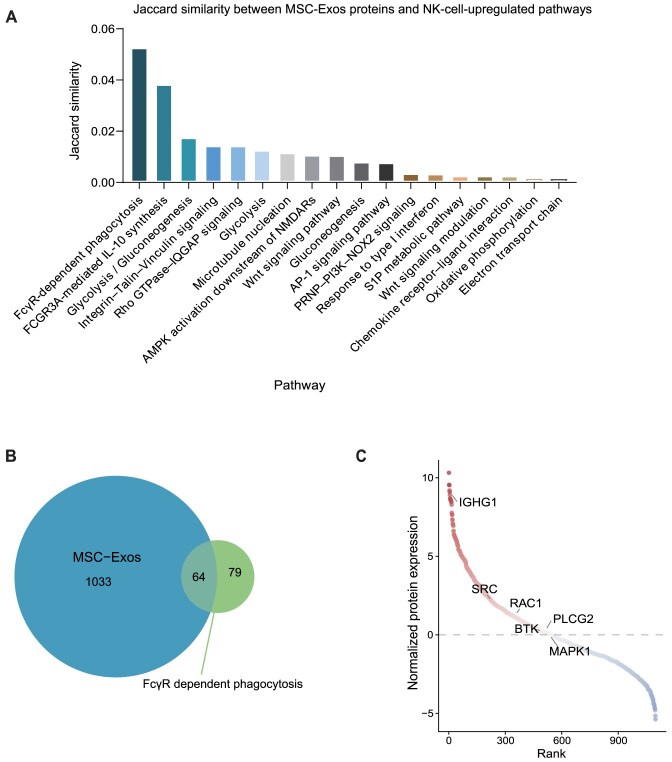
Proteomic characterization of MSC-Exos and identification of FcγR-related signaling modules. (A) Bar plot showing Jaccard similarity between gene sets represented by proteins identified in the MSC-Exos proteome and gene sets from pathways upregulated in NK cells by transcriptomic analysis. (B) Venn diagram showing the overlap between proteins identified in MSC-Exos and proteins annotated to the Reactome FcγR-dependent phagocytosis pathway. (C) Scatter plot showing the ranked normalized protein abundance in MSC-Exos, with selected components associated with the FcγR-dependent phagocytosis pathway (IGHG1, SRC, RAC1, PLCG2, BTK, and MAPK1) highlighted.

To relate exosomal protein composition to transcriptional programs upregulated in MSC-Exos-treated NK cells, we calculated the Jaccard similarity between gene sets represented by proteins identified in the MSC-Exos proteome and gene sets from pathways upregulated in NK cells by transcriptomic analysis. Among the top overlapping Reactome terms were FcγR-dependent phagocytosis, *FCGR3A*-mediated IL-10 synthesis, and glycolysis/gluconeogenesis (Fig. 5A). As NK cells are not professional phagocytes, we interpret the Reactome annotation “FcγR-dependent phagocytosis” in an NK cell context, where it more likely reflects FcγRIIIa/CD16-linked programs involving cytoskeletal remodeling, immune-synapse organization, membrane trafficking, and trogocytosis-like membrane transfer during Fc-mediated engagement, rather than canonical antibody-dependent cellular phagocytosis (ADCP). Venn analysis identified 64 proteins shared between the MSC-Exos proteome and the Reactome FcγR-dependent phagocytosis pathway gene set (Fig. 5B). This overlap included proximal kinases (e.g., SRC, BTK), downstream effectors (e.g., PLCG2, MAPK1), and cytoskeletal regulators (e.g., RAC1), which were involved in receptor-proximal signaling and immune-synapse organization [24–26]. Ranking by normalized protein abundance showed that several of these components were among the more abundant proteins detected in MSC-Exos (Fig. 5C), supporting their potential relevance to FcγR-associated signaling biology. We note that immunoglobulin-related proteins (e.g., IGHG1) detected in exosome proteomics might reflect co-isolation with MSC-Exos-associated proteins or surface adsorption during processing, rather than serum IgG carryover; therefore, their presence is interpreted with caution and is not used as standalone evidence for functional cargo delivery.

Together, these proteomic signatures are consistent with and support the hypothesis that MSC-Exos contain FcγR/CD16-associated signaling components that may be linked to a primed signaling state in NK cells. However, pathway overlap and proteomic enrichment by themselves do not establish functional cargo delivery to NK cells, nor do they constitute evidence of enhanced antibody-dependent cellular cytotoxicity (ADCC). Future mechanistic studies, including uptake and transfer assays, functional blockade, and targeted perturbation, will be required to test whether specific exosomal components are transferred to NK cells and whether they are necessary for the enhanced FcγR/CD16-associated transcriptional and phenotypic features observed in our system.

## Discussion

NK cells act as a key component of the innate immune system, but their clinical application is limited by functional decline and metabolic exhaustion during large-scale *ex vivo* expansion [27, 28]. In this study, MSC-Exos applied during *ex vivo* NK cell expansion were associated with improved NK cell fitness and a cytotoxicity-biased functional profile. Our data support a framework in which MSC-Exos may contribute to an efficient, lower-stress NK cell state, while the underlying causal mechanisms remain to be validated.

MSC-Exos are often described as immunosuppressive in other settings [29, 30], underscoring the context-dependent nature of their effects. In our expansion setting, baseline cytokine support and culture conditions may establish a pre-activated state that shifts MSC-Exos function toward supporting metabolic adaptation and effector readiness rather than broadly dampening immune activity. Importantly, the phenotype observed in *ex vivo*-expanded NK cells was not one of generalized inflammatory amplification. Instead, it is consistent with functional skewing in which cytotoxic output is enhanced while inflammatory cytokine production is restrained. This interpretation is consistent with emerging evidence that MSC-derived factors can, under specific priming conditions, be repurposed to support rather than suppress immune surveillance [31]. Notably, IFNG mRNA and IFN-γ protein were reduced despite increased degranulation and activating-receptor expression; this pattern is compatible with partial uncoupling of cytotoxicity and cytokine programs, which are governed by overlapping but distinct regulatory circuits [32–34]. Such a cytotoxic state with restrained inflammatory output may be advantageous for therapeutic manufacturing, as it preserves killing capacity while potentially limiting cytokine-associated toxicity.

At the mechanistic level, our data implicate multiple convergent regulatory axes that could collectively influence NK cell behavior, although the current evidence is primarily correlative. One plausible axis involves redox control and mitochondrial homeostasis. NRF2 is a central transcriptional regulator that couples antioxidant programs to mitochondrial resilience [35, 36] and has been linked to the metabolic robustness required for NK cell persistence and function in stressful microenvironments [22]. In our results, increased NRF2 mRNA expression together with improved mitochondrial readouts was consistent with enhanced stress-buffering capacity in MSC-Exos-treated NK cells. Future work should therefore directly assess NRF2 dependence using loss-of-function strategies (e.g., genetic silencing, CRISPR interference, or pharmacologic inhibition) to determine whether the MSC-Exos-associated enhancement of mitochondrial membrane potential, degranulation, and cytotoxic capacity is attenuated by NRF2 blockade.

A second candidate axis involves FcγR/CD16-related signaling readiness. Concordance between MSC-Exos proteomics and NK cell transcriptomic changes suggests enrichment of FcγR-associated components and CD16-related programs, which is consistent with a plausible hypothesis that MSC-Exos exposure may facilitate FcγR/CD16-linked activation states. However, this study does not directly demonstrate enhanced ADCC, nor does it establish functional transfer and requirement of specific exosomal proteins within recipient NK cells. These possibilities could be tested by (i) directly measuring antibody-triggered ADCC in standardized assays, (ii) tracing exosome uptake and assessing receptor-proximal signaling events (e.g., phosphorylation of FcγR pathway nodes), and (iii) evaluating sensitivity to pathway inhibition (e.g., BTK/PLCG2/SRC perturbation) to determine whether any observed augmentation depends on these components.

Beyond proteins, MSC-Exos may also exert effects through non-protein cargo such as microRNAs, which can reprogram signaling and transcriptional states in recipient cells [17, 37]. Dedicated profiling and functional interrogation of MSC-Exos microRNA cargo will therefore be important to define the contributions of distinct cargo classes. Finally, the limited number of donors and the evident inter-donor variability across functional readouts should be recognized as important limitations of this study. While MSC-Exos-associated functional benefits were observed across donors, the magnitude of these effects was variable, highlighting the need to identify baseline predictors of responsiveness, such as mitochondrial fitness or activating-receptor repertoire. Future work in larger donor cohorts should further define these predictors and evaluate the durability of MSC-Exos-associated gains through long-term persistence studies, serial killing assays, and ultimately *in vivo* models.

Our data support a testable framework in which MSC-Exos are linked to coordinated immunometabolic remodeling and a bias toward effector-like maturation in *ex vivo*-expanded human NK cells, while highlighting the need for targeted mechanistic studies to define the underlying causal pathways and the mode of exosomal cargo action.

## Methods

### Isolation, purification, and characterization of MSC-Exos

Human umbilical cord-derived MSCs (hUC-MSCs; Cyagen Biosciences, China) were cultured in Xeno-Free Human Mesenchymal Stem Cell Medium (Applied Cell, China) at 37°C in a humidified incubator with 5% CO₂. According to the manufacturer, this xeno-free medium is produced under serum-free conditions and contains no exogenous animal-derived protein components; therefore, conditioned medium for MSC-Exos isolation was collected under serum-free culture conditions. Conditioned medium was collected and stored at −80°C until further processing.

MSC-Exos were isolated from MSC culture supernatants using SEC, following previously published SEC optimization protocols [38]. A Sepharose CL-6B column (10 mL bed volume) was pre-equilibrated with phosphate-buffered saline (PBS) and stored overnight at 4°C to stabilize the resin. Supernatants were sequentially centrifuged at 300 × g for 5 min and 3,000 × g for 5 min to remove cells and debris, followed by filtration through a 0.22 μm membrane. The filtrate was concentrated to 1 mL using a 50 kDa molecular weight cut-off ultrafiltration device (3,000 × g for 30 min) and loaded onto the CL-6B column. After loading, the column was eluted with PBS in 500 μL increments. The first 2 mL of flow-through was discarded, and subsequent fractions were collected in 2 mL volumes. Exosome-rich SEC fractions were pooled based on high particle counts and minimal soluble-protein carryover, operationalized by a higher particle-to-protein ratio as a relative purity metric, consistent with published SEC optimization protocols.

Purified vesicles were characterized using orthogonal approaches. Morphology was assessed by TEM, particle size distribution was measured by NTA, and exosomal identity was confirmed by immunoblotting for positive markers (CD63, TSG101, and Syntenin) and the absence of the negative marker Calnexin.

### *Ex vivo* expansion of peripheral blood–derived NK cells and intervention with MSC-Exos

All human PBMCs were obtained under approval from the Drug Clinical Trial Ethics Committee of Liaocheng Second People’s Hospital and BGI Research. PBMCs from healthy donors were expanded *ex vivo* for 16 days using a commercial NK cell expansion kit (Jiake Biotechnology, China) to generate NK cell products. Beginning on day 10 of culture, the MSC-Exos-treated group received MSC-Exos at final concentrations of 10–40 μg/mL in NK cell expansion medium. Fresh medium containing the corresponding exosome concentration was replenished every 3 days. The CON was maintained under identical conditions but received exosome-free medium during medium changes.

### CCK-8 assay for evaluating the effect of MSC-Exos on NK cell viability

To assess the effect of MSC-Exos on NK cell viability, expanded NK cells were collected on day 10 and seeded into 96-well plates at 5 × 10^4^ cells per well in a total volume of 100 μL, with at least three replicate wells per condition. Cells were then cultured either in NK cell expansion medium alone (CON) or in the same medium supplemented with MSC-Exos (EXO) (10–40 μg/mL). Cell viability was measured on days 11, 13, and 16, corresponding to 1, 3, and 6 days after intervention, respectively. Medium-only wells were included as blanks.

For the day 16 measurement, 50% of the medium was replaced on day 13 with fresh medium containing the corresponding treatment, with MSC-Exos added to the EXO group at the same final concentration. At each indicated time point, 10 μL of CCK-8 reagent was added to each well, followed by incubation at 37°C for 2 h in the dark. The OD was measured at 450 nm using a microplate reader, and following blank subtraction, the absorbance values were used to calculate relative cell viability. Data are presented as mean ± standard deviation (SD) from at least three replicate wells. Relative cell viability was calculated using the following formula:


\begin{eqnarray*}
{\mathrm{Relative\ activity}} = \frac{{\left( {{\mathrm{O}}{{{\mathrm{D}}}_{{\mathrm{sample}}}} - {\mathrm{O}}{{{\mathrm{D}}}_{{\mathrm{blank}}}}} \right)}}{{\left( {{\mathrm{O}}{{{\mathrm{D}}}_{{\mathrm{control}}}} - {\mathrm{O}}{{{\mathrm{D}}}_{{\mathrm{blank}}}}} \right)}}.
\end{eqnarray*}

### Induction of senescence in HDFs

Primary HDFs (Jinyuan Biotechnology, China) were cultured in DMEM/Ham’s F-12 (1:1) supplemented with 10% fetal bovine serum (FBS) and 4 mM l-glutamine at 37°C in a humidified 5% CO₂ atmosphere. To induce senescence, confluent HDFs were treated with DOX for 48 h. After treatment, medium was replaced with fresh complete medium, and cells were incubated for an additional 4–6 days to allow full development of senescence-associated phenotypes. Senescence was quantified using the Senescence β-Galactosidase Staining Kit (MedChemExpress, USA) according to the manufacturer’s instructions. Cultures were considered significantly senescent when >50% of cells were positive for β-galactosidase staining.

### qRT-PCR

Total RNA was extracted using the FastPure Cell/Tissue Total RNA Isolation Kit V2 (Vazyme, China). RNA concentration and purity were assessed with a NanoDrop 2000 spectrophotometer (Thermo Fisher Scientific, USA). Complementary DNA (cDNA) was synthesized from 1 μg of total RNA using the HiScript II Q RT SuperMix for qPCR kit (Vazyme, China). qRT-PCR was performed using SYBR Green master mix (Yeasen, China) and primers (Supplementary Table S3) on a QuantStudio 5 Real-Time PCR System (Applied Biosystems, USA). Relative gene expression was calculated using the 2^−ΔΔCt^ method and normalized to *ACTB*.

### *In vitro* cytotoxicity assay of NK cells

NK cell cytotoxicity was assessed using the CytoTox 96® Non-Radioactive Cytotoxicity Assay (Promega, USA), which quantifies lactate dehydrogenase (LDH) release following target-cell lysis. For antitumor activity, NK cells from EXO and CON groups were co-cultured with K562 target cells at effector-to-target (E:T) ratios of 5:1 and 20:1 for 5 h. To assess senescent-cell clearance, NK cells were co-cultured with DOX-induced senescent HDFs at identical E:T ratios for 20 h. Prior to co-culture, HDFs were trypsinized and counted to ensure accurate E:T setup.

After incubation, supernatants were collected and the OD was measured at 490 nm with a reference wavelength of 680 nm using a microplate reader. Cytotoxicity was calculated according to the manufacturer’s instructions. All experiments were performed in triplicate, and data are presented as mean ± SD. NK cell-mediated cytotoxicity was calculated using the following formula:


\begin{eqnarray*}
&&{\mathrm{NK\ cytotoxicity\ (\% )}}\\&&\quad = \frac{{\left( {{\mathrm{O}}{{{\mathrm{D}}}_{{\mathrm{experimental}}}} - {\mathrm{O}}{{{\mathrm{D}}}_{{\mathrm{spontaneous\ target}}}} - {\mathrm{O}}{{{\mathrm{D}}}_{{\mathrm{spontaneous\ effector}}}}} \right)}}{{\left( {{\mathrm{O}}{{{\mathrm{D}}}_{{\mathrm{maximum\ release}}}} - {\mathrm{O}}{{{\mathrm{D}}}_{{\mathrm{spontaneous\ target}}}}} \right)}} \times 100{\mathrm{\% }}.
\end{eqnarray*}

### Flow cytometry

For cell surface staining, single-cell suspensions were incubated with fluorochrome-conjugated antibodies against CD56, CD3, CD16, LAG3, NKG2D, NKG2C, and CD107a at 4°C for 20 min in the dark, followed by washing with FACS buffer. For intracellular staining, cells were fixed and permeabilized using the Cyto-Fast™ Fix/Perm Buffer Set (Biolegend, USA) and then incubated with antibodies against granzyme B at room temperature for 20 min in the dark. Samples were acquired on a BD FACSAria III flow cytometer and analyzed using FlowJo (v10.0).

Mitochondrial membrane potential was assessed using the JC-1 Mitochondrial Membrane Potential Assay Kit (Yeasen, China). Mitochondrial mass and activity were evaluated using MitoTracker® Green FM and MitoTracker® Red CMXRos (Yeasen, China), respectively, followed by flow cytometric detection.

### Cytometric bead array measurement of IFN-γ

MSC-Exos-treated and control NK cells were co-cultured with K562 target cells at an E:T ratio of 5:1 for 12 h. Cell suspensions were centrifuged at 500 × g for 5 min, and supernatants were collected for cytokine quantification. IFN-γ concentrations were measured using the Cytometric Bead Array (CBA) Human Soluble Protein Master Buffer Kit (BD Biosciences, USA) according to the manufacturer’s instructions. Capture beads were incubated with supernatants for 1 h at room temperature in the dark, followed by addition of PE-conjugated detection reagent for 2 h to form bead–cytokine–detector complexes. Beads were washed, resuspended, acquired on a BD FACSAria III, and analyzed using FCAP Array software. Results are reported as pg/mL based on standard curves.

### Single-cell RNA-seq data processing and analysis

Raw sequencing reads were filtered, demultiplexed, and aligned to the hg38 human reference genome using a custom pipeline [39]. Only reads aligned to annotated gene exons were counted. Potential doublets were identified and removed using DoubletFinder (v2.0.3), and ambient RNA contamination was corrected using SoupX (v1.4.8) under default parameters.

Cells were retained based on the following quality thresholds: 500–20,000 UMIs and 500–6,000 detected genes, and <10% mitochondrial gene content. Downstream analyses were performed using Scanpy (v1.9.3) in Python 3.7. After library-size normalization and log transformation, the 2,000 most variable genes were selected. UMI counts and mitochondrial percentages were regressed out, and the expression matrix was scaled. Dimensionality reduction was performed by principal component analysis (PCA), followed by batch correction with Harmony (v0.0.10) with theta = 5 and max.iter.harmony = 50; all other parameters were kept at their default values. The top 30 Harmony-corrected principal components were used to construct a k-nearest neighbor (kNN) graph (*k* = 15). Clustering was performed using the Leiden algorithm (Scanpy implementation) with resolution = 0.1, and clusters were annotated based on canonical marker genes.

Major immune lineages were annotated using canonical marker sets (T cell markers: *CD3D, CD3E, CD4, IL7R, CD8A, CD8B*; B cell markers: *CD79A, MS4A1*; NK cell markers: *NCAM1, KLRD1, FCGR3A, GNLY, NKG7*). Non-NK lineage cells were excluded prior to generating the NK cell-only subset used for integration, clustering, DE, and trajectory inference. Additionally, clusters lacking canonical NK cell markers and exhibiting low marker specificity were considered transcriptionally ambiguous and removed to reduce the influence of low-quality cells, potential doublets, or uninformative populations. After subsetting to NK cells, batch correction was re-applied with Harmony (using theta = 2 and max.iter.harmony = 50), and the kNN graph was recomputed, followed by de novo UMAP embedding and Leiden clustering (with resolution = 0.4) on the NK cell-only dataset. NK cell subclusters were then annotated and used for downstream comparisons between the MSC-Exos-treated and untreated conditions. DE analysis between the CON and EXO groups was performed using Scanpy. Genes with |log₂(fold change)| > 0.25 and an adjusted *P*-value < 0.05 (Benjamini–Hochberg corrected) were identified as DEGs.

### Pseudotime trajectory analysis

Pseudotime trajectory analysis was performed on NK cells extracted from the integrated scRNA-seq dataset to infer differentiation dynamics. For Monocle 2 (v2.26.0), we selected ordering genes as cell-type differential genes across the four NK cell subclusters (qval < 0.01) and reduced dimensionality using DDRTree to reconstruct branched trajectories; the root state was determined automatically by Monocle 2. In parallel, we used Monocle 3 (v1.0.0) with UMAP for dimensionality reduction to validate the global differentiation manifold. The pseudotime root node was specified based on cell-type annotation, using cells from the CD56^bright^ subset as the starting population. Together, these complementary analyses mapped a continuum from early regulatory-like states toward mature cytotoxic effector phenotypes based on dynamic, lineage-associated transcriptional programs.

### Metabolic profiling

Metabolic pathway activity scores were computed using scMetabolic (v0.2.1) with the built-in KEGG metabolism gene sets under default settings, enabling single-cell level comparison of energy metabolism and biosynthetic programs between treatment groups.

### Gene ontology and GSEA

GO enrichment analyses were performed in R using clusterProfiler (SCR_016,884). The enrichGO function was executed with Benjamini–Hochberg correction and a false discovery rate (FDR) threshold of *q* < 0.01. Curated gene sets (c2.cp.v2025.1.Hs.symbols.gmt) were obtained from the Molecular Signatures Database (MSigDB). For single-sample and preranked enrichment analyses, GSEApy (SCR_025,803, v1.1.8) was used in Python, with parameters set to min_size = 5, max_size = 1,000, and 1,000 permutations. Gene sets with FDR *q* < 0.25 were considered significantly enriched.

### Exosome proteomic analysis

Purified MSC-Exos were resuspended in lysis buffer containing 8 M urea and 1× protease inhibitor cocktail. Samples were lysed by ultrasonication and centrifuged at 25,000 × g for 15 min at 4°C to collect supernatants for protein quantification. Proteins were reduced with 10 mM dithiothreitol (DTT) at 37°C for 30 min and alkylated with 55 mM iodoacetamide (IAM) in the dark for 45 min. Trypsin was added at a substrate-enzyme ratio of 1:50 (w/w) and digested overnight at 37°C. The peptides were purified and desalted by C18 column, concentrated under vacuum, and reconstituted with mobile phase (ultrapure water containing 0.1% formic acid) for analysis by liquid chromatography-tandem mass spectrometry (LC-MS/MS).

Raw mass spectrometry data were processed using Spectronaut 17 (Biognosys) and searched against the UniProt Homo sapiens database (20,360 Swiss-Prot entries) using the directDIA+ (Deep) workflow. This library-free DIA strategy was coupled with label-free quantification (LFQ) to enable high-coverage protein identification and accurate quantification without a prebuilt spectral library, generating a global proteomic landscape and differential abundance profiles for MSC-Exos.

### Statistical analysis

Data are presented as mean ±SD, unless otherwise stated in the figure legends. Statistical analyses were performed using GraphPad Prism 8.3.1. For comparisons between two groups, a two-tailed Student’s *t*-test was used for normally distributed data, with paired or unpaired tests applied according to the experimental design. For non-normally distributed data, group comparisons were performed using a two-sided Mann–Whitney *U* test. For single-cell transcriptomic analyses, DE between groups was assessed using a two-sided Wilcoxon rank-sum test. Statistical significance was defined as *P < 0.05, **P < 0.01, ***P < 0.001, and ^****^P < 0.0001. The definition of n (biological replicates vs. technical replicates) and the specific test used for each analysis are provided in the corresponding figure legends.

## Availability of source code and requirements

Project name: NK-MSC-exos-scRNAseq-analysis

Project homepage: https://github.com/fuyunyun-95/NK-MSC-exos-scRNAseq-analysis.git

Operating system: Linux

Programming language: Python and R

Package management: Conda

Hardware requirements: Tested on a laptop with 8-core CPU, 64 GB RAM and 256 GB SSD

License: MIT License

## Additional files

**Supplementary Fig. S1:** Additional evaluation of MSC-Exos treatment dose and duration during NK cell expansion. (A) Quantification of NK cell viability in donors NK002, NK003, and NK004 by CCK-8 assay (OD450) following treatment with MSC-Exos at 10–40 μg/mL, measured on day 16. (B) Quantification of NK cell viability in donor NK005 by CCK-8 assay (OD450) following treatment with 20 μg/mL MSC-Exos, measured on days 11, 13, and 16. (C) Quantification of NK cell viability in donor NK006 by CCK-8 assay (OD450) following treatment with MSC-Exos at 10–40 μg/mL, measured on days 11, 13, and 16.(D) Representative flow cytometry plots showing the frequencies of CD56⁺CD3⁻ NK cells in the CON and EXO groups across multiple donors.

Statistics: For panels A and C, data are presented as mean ± SD from three technical replicates. For panel B, data are presented as mean ± SD from four technical replicates. Statistical significance was assessed using an unpaired two-tailed Student’s *t*-test. ns, not significant; *P < 0.05, **P < 0.01.

**Supplementary Fig. S2:** Doxorubicin-induced senescence in human dermal fibroblasts and additional assessment of NK cell effector responses. (A) Viability of HDFs treated with increasing concentrations of DOX (0–250 μM) for 48 h, measured by CCK-8 assay (OD450). The 0 μM group served as the control. (B) Representative bright-field images showing morphological changes and SA-β-Gal staining in HDFs. HDFs-CON indicates untreated control cells, and HDFs-DOX indicates cells treated with 150 nM DOX for 48 h. Quantification of SA-β-Gal-positive cells is shown on the right. Scale bar, 200 μm. (C) Relative mRNA expression of senescence- and inflammation-associated genes in HDFs, as determined by RT-qPCR. (D) Percentage lysis of K562 tumor cells by NK cells from different donors in the EXO and CON groups. (E) Percentage lysis of DOX-induced senescent HDFs by NK cells from different donors in the EXO and CON groups. The effector-to-target (E:T) ratio was 5:1 for both assays.

Statistics: For panels A–E, data are presented as mean ± SD from three technical replicates. Statistical significance was assessed using an unpaired two-tailed Student’s *t*-test. ns, not significant; *P < 0.05, **P < 0.01, ***P < 0.001.

**Supplementary Fig. S3:** MSC-Exos are associated with altered NK cell secretory output and metabolic pathway enrichment. (A) CBA measurement of IFN-γ secretion in culture supernatants from NK cells in the CON and EXO groups. (B) GSEA plots showing enrichment of oxidative phosphorylation and interferon-α/β signaling pathways in the EXO group. (C) Bar plot showing differential enrichment of metabolic pathways. Green bars represent pathways upregulated in the EXO group, while orange bars represent downregulated pathways.

Statistics: For panel A, data are presented as mean ± SD from three technical replicates. Statistical significance was assessed using an unpaired two-tailed Student’s *t*-test. ***P < 0.001.

**Supplementary Fig. S4:** Harmony integration improves donor mixing while retaining condition-associated structure. (A) UMAP plots colored by donors (NK001, NK002, NK003, NK004) before and after Harmony integration, showing improved donor mixing after batch correction. (B) UMAP plots colored by treatment condition (CON vs. EXO) before and after Harmony integration, indicating that condition-associated structure remains observable after correction. (C) UMAP plots colored by batch before and after Harmony integration, showing reduced batch-associated separation after correction. (D) Stacked bar plots showing the proportions of annotated NK cell subclusters for each donor under CON and EXO treatment conditions.

**Supplementary Fig. S5:** Overview of proteomic profiling and peptide features of MSC-derived exosomes. (A) Bar plot showing the total number of quantified proteins identified in MSC-Exos by LC–MS/MS. Data are shown from three technical replicates. (B) Length distribution of peptides identified in MSC-Exos. (C) Distribution of the number of unique peptides identified per protein in MSC-Exos. (D) Distribution of sequence coverage across proteins identified in MSC-Exos.

**Supplementary Table S1:** MSC-Exos dosing metrics (protein-, particle-, and cell-normalized).

**Supplementary Table S2:** Donor information for peripheral blood-derived NK cells.

**Supplementary Table S3:** Primer sequences used for qRT-PCR.

**Supplementary Table S4:** Donor-by-condition cell counts during scRNA-seq processing (pre- and post-filtering).

**Supplementary Table S5:** Donor-by-condition cell counts for annotated NK subclusters (n_cells).

**Supplementary Table S6:** Protein data of MSC-Exos by Mass Spectrometry.

**Supplementary Table S7:** Protein coverage of MSC-Exos by Mass Spectrometry.

**Supplementary Table S8:** Protein normalized expression of MSC-Exos by Mass Spectrometry.

## Abbreviations

ADCC: antibody-dependent cellular cytotoxicity; ADCP: antibody-dependent cellular phagocytosis; CBA: cytometric bead array; CCK-8: cell counting kit-8; cDNA: complementary DNA; CNSA: China National GeneBank Sequence Archive; CON: the control group; DE: differential expression; DEGs: differentially expressed genes; DOX: doxorubicin; DTT: dithiothreitol; EXO: the MSC-Exos-treated group; FBS: fetal bovine serum; GSA: Genome Sequence Archive; GSEA: gene set enrichment analysis; GvHD: graft-versus-host disease; HDFs: human dermal fibroblasts; IAM: iodoacetamide; LC–MS/MS: liquid chromatography-tandem mass spectrometry; LDH: lactate dehydrogenase; LFQ: label-free quantification; MFI: mean fluorescence intensity; MISEV: Minimal Information for Studies of Extracellular Vesicles; MSCs: mesenchymal stem cells; MSC-Exos: mesenchymal stem cell-derived exosomes; NK: natural killer; NTA: nanoparticle tracking analysis; non-MHC: non-major histocompatibility complex; OD: optical density; PBMCs: peripheral blood mononuclear cells; PBS: phosphate-buffered saline; PCA: principal component analysis; qRT-PCR: quantitative real-time polymerase chain reaction; SASP: senescence-associated secretory phenotype; SA-β-Gal: senescence-associated β-galactosidase; SD: standard deviation; scRNA-seq: single-cell RNA sequencing; SEC: size-exclusion chromatography; TEM: transmission electron microscopy.

## Author contributions

Y.Y.F.: Investigation, Methodology, Formal analysis, Validation, Visualization, Writing—original draft; Y.l.: Methodology, Formal analysis, Visualization, Writing—review & editing; M.X.: Formal analysis, Visualization; G.L.: Formal analysis, Visualization; J.S.: Formal analysis, Visualization; F.B.: Formal analysis, Visualization; W.X.: Validation; J.Z.: Validation; J.L.: Validation; Q.G.: Methodology; Y.H.: Resources; F.X.: Resources; S.L.: Resources, Project administration; L.L.: Resources; Y.F.: Supervision, Writing—review & editing; X.D.: Conceptualization, Project administration, Supervision, Resources, Writing—review & editing.

## Competing interests

The authors declare that they have no competing interests.

## Supplementary Material

giag049_Supplemental_Files

giag049_Authors_Response_To_Reviewer_Comments_original_submission

giag049_GIGA-D-26-00032_original_submission

giag049_GIGA-D-26-00032_revision_1

giag049_Reviewer_1_Report_original_submission Reviewer 1 -- 2/10/2026

giag049_Reviewer_1_Report_revision_1 Reviewer 1 -- 3/3/2026

giag049_Reviewer_2_Report_original_submission Reviewer 2 -- 2/10/2026

giag049_Reviewer_2_Report_revision_1 Reviewer 2 -- 3/3/2026

giag049_Reviewer_3_Report_original_submission Reviewer 3 -- 2/12/2026

giag049_Reviewer_3_Report_revision_1 Reviewer 3 -- 3/4/2026

## Data Availability

The scRNA-seq data generated in this study and supporting its findings have been deposited in the China National GeneBank Sequence Archive (CNSA) [40] under accession number CNP0008912 and in the Genome Sequence Archive (GSA) under accession number PRJCA057478. The mass spectrometry proteomics data have been deposited to the ProteomeXchange Consortium via the PRIDE partner repository with the accession number PXD073707.
