# Supplementary material for: Integrative single-cell transcriptomics and proteomics reveal an immunometabolic framework for MSC-exosome-mediated remodeling of expanded NK cells
Source: Gigascience. 2026 Apr 20;15:giag049. doi: 10.1093/gigascience/giag049 (PMC13273429; doi:10.1093/gigascience/giag049)
Supplement: giag049_Supplemental_Files [file giag049_Supplemental_Files.zip › Supplementary Figures_260330.docx]

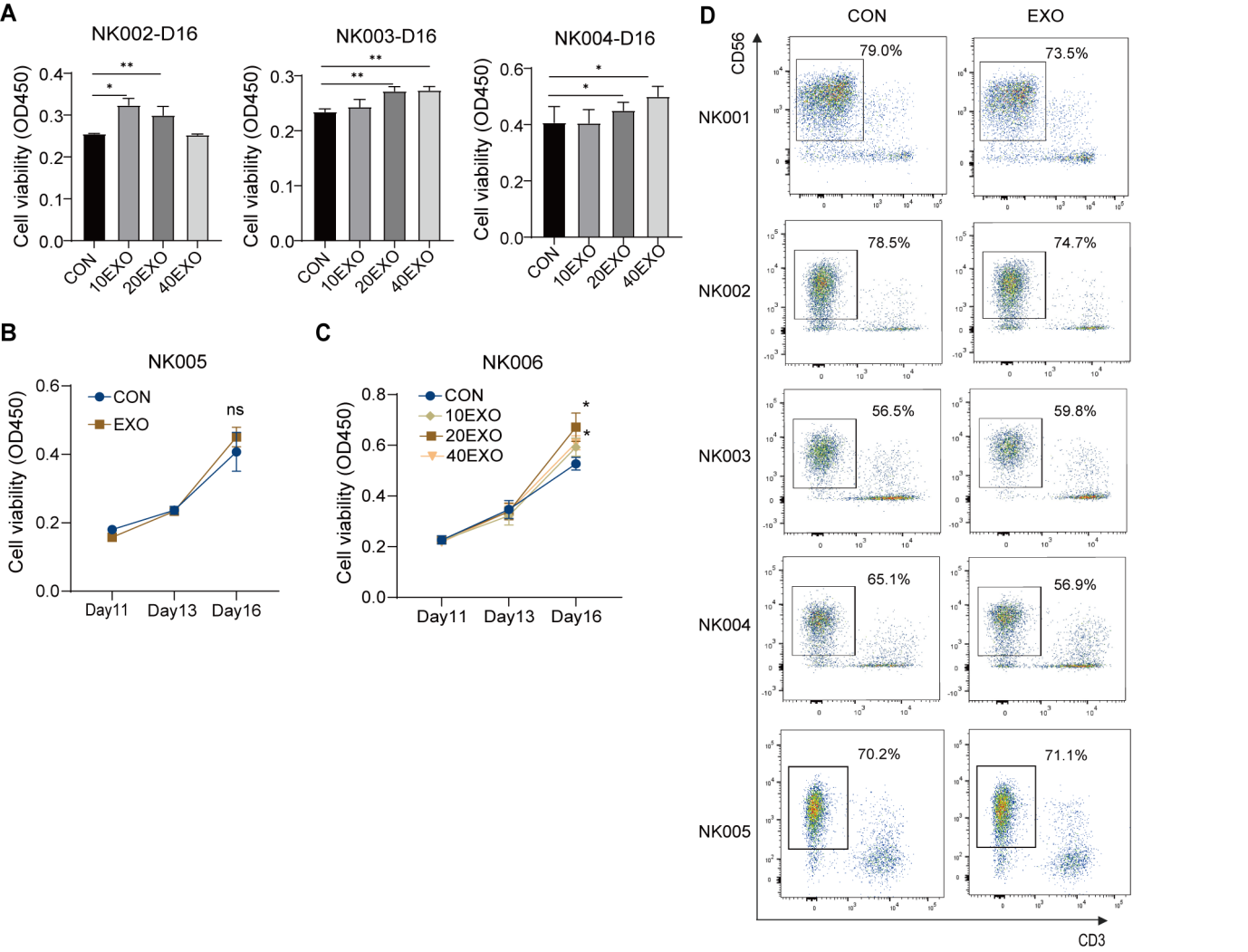


**Supplementary Figure S1. Additional evaluation of MSC-Exos treatment dose and duration during NK-cell expansion.**

**(A)** Quantification of NK-cell viability in donors NK002, NK003, and NK004 by CCK-8 assay (OD450) following treatment with MSC-Exos at 10–40 μg/mL, measured on day 16. **(B)** Quantification of NK-cell viability in donor NK005 by CCK-8 assay (OD450) following treatment with 20 μg/mL MSC-Exos, measured on days 11, 13, and 16. **(C)** Quantification of NK-cell viability in donor NK006 by CCK-8 assay (OD450) following treatment with MSC-Exos at 10–40 μg/mL, measured on days 11, 13, and 16. **(D)** Representative flow cytometry plots showing the frequencies of CD56⁺CD3⁻ NK cells in the CON and EXO groups across multiple donors.

Statistics: For panels A and C, data are presented as mean ± SD from three technical replicates. For panel B, data are presented as mean ± SD from four technical replicates. Statistical significance was assessed using an unpaired two-tailed Student’s t-test. ns, not significant; *P < 0.05, **P < 0.01.


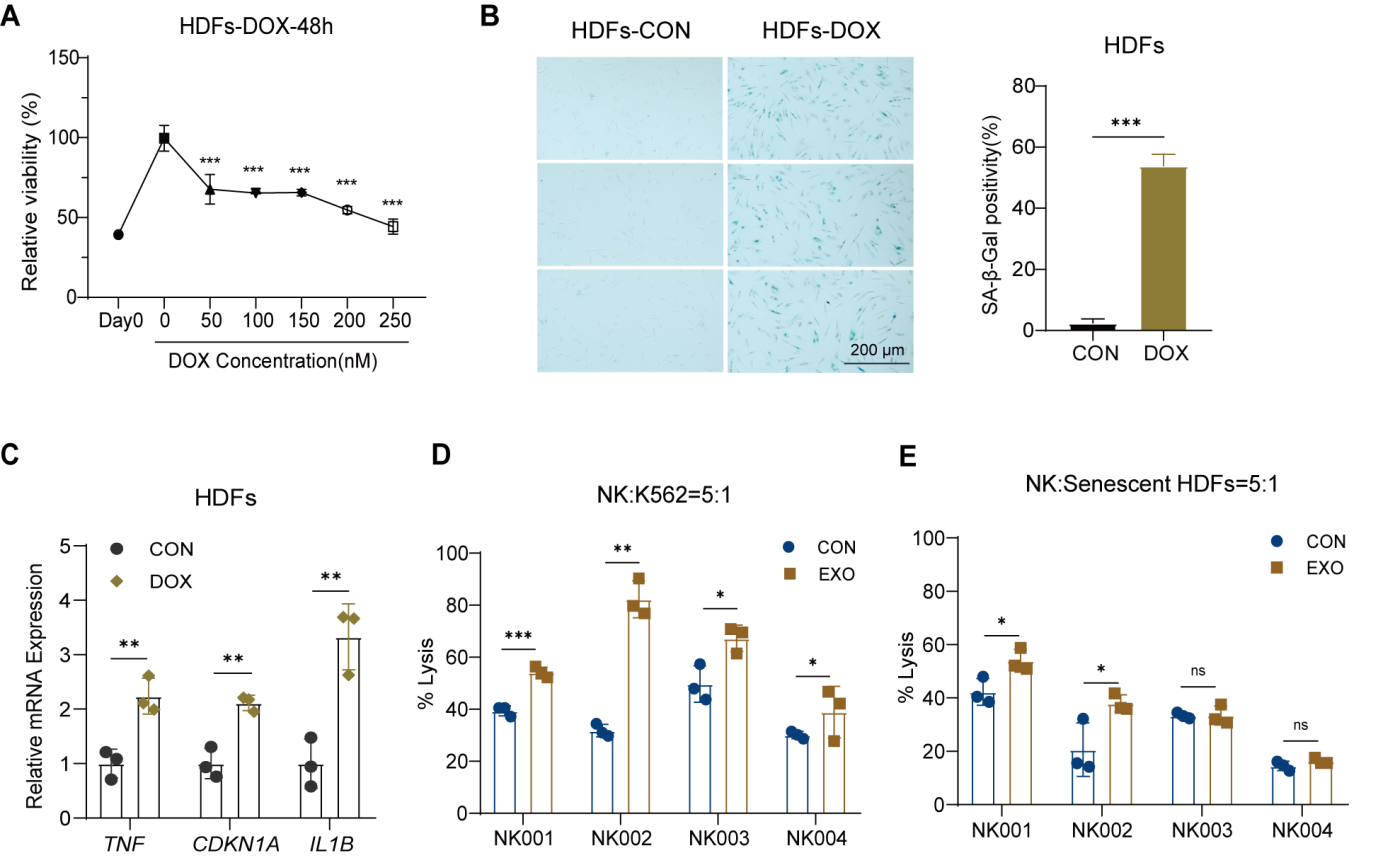


**Supplementary Figure S2. Doxorubicin-induced senescence in human dermal fibroblasts and additional assessment of NK-cell effector responses.**

**(A)** Viability of HDFs treated with increasing concentrations of DOX (0–250 μM) for 48 h, measured by CCK-8 assay (OD450). The 0 μM group served as the control. **(B)** Representative bright-field images showing morphological changes and SA-β-Gal staining in HDFs. HDFs-CON indicates untreated control cells, and HDFs-DOX indicates cells treated with 150 nM DOX for 48 h. Quantification of SA-β-Gal-positive cells is shown on the right. Scale bar, 200 μm. **(C)** Relative mRNA expression of senescence- and inflammation-associated genes in HDFs, as determined by RT-qPCR. **(D)** Percentage lysis of K562 tumor cells by NK cells from different donors in the EXO and CON groups. **(E)** Percentage lysis of DOX-induced senescent HDFs by NK cells from different donors in the EXO and CON groups. The effector-to-target (E:T) ratio was 5:1 for both assays.

Statistics: For panels A–E, data are presented as mean ± SD from three technical replicates. Statistical significance was assessed using an unpaired two-tailed Student’s t-test. ns, not significant; *P < 0.05, **P < 0.01, ***P < 0.001.


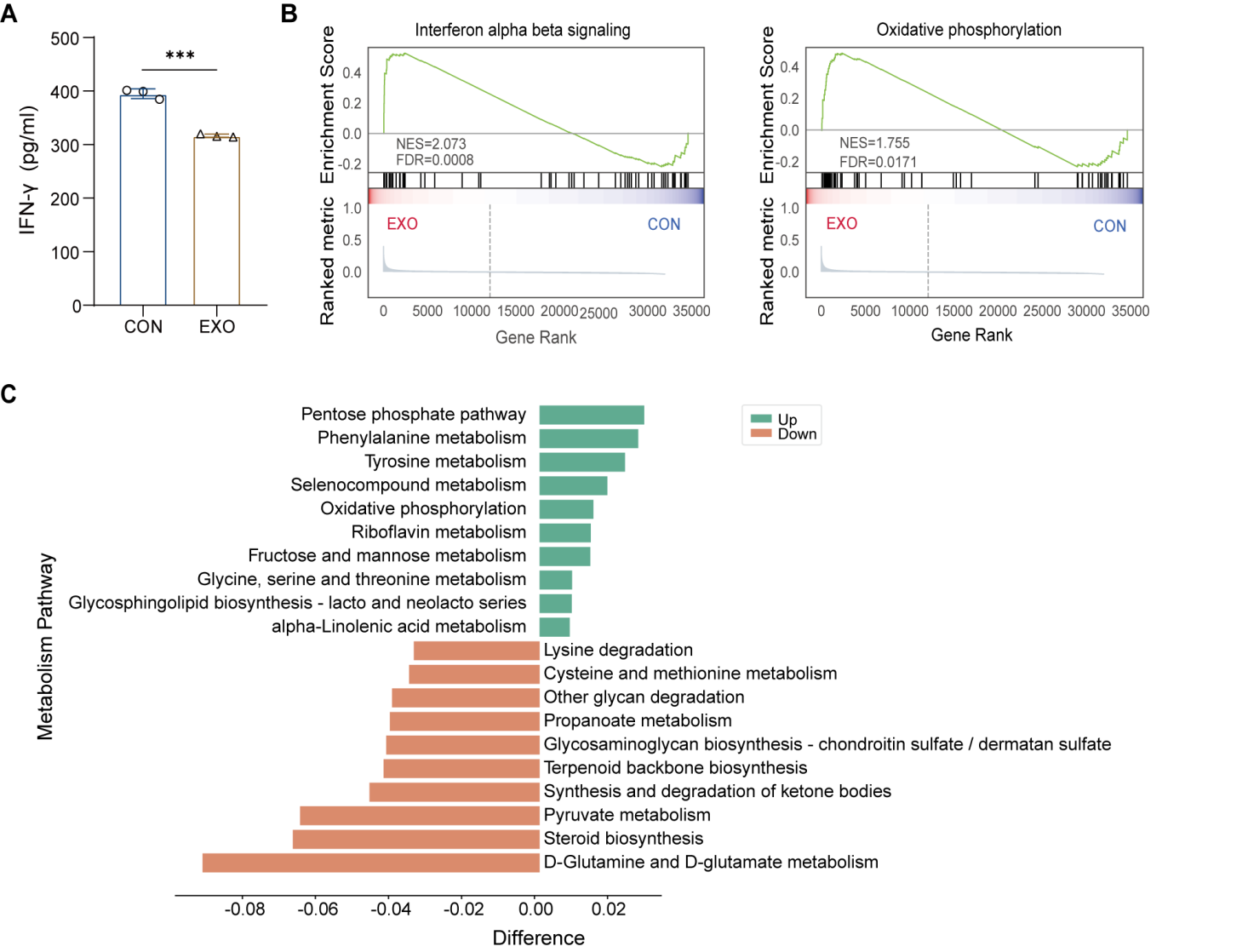


**Supplementary Figure S3. MSC-Exos are associated with altered NK-cell secretory output and metabolic pathway enrichment.**

**(A)** CBA measurement of IFN-γ secretion in culture supernatants from NK cells in the CON and EXO groups. **(B)** GSEA plots showing enrichment of oxidative phosphorylation and interferon-α/β signaling pathways in the EXO group. **(C)** Bar plot showing differential enrichment of metabolic pathways. Green bars represent pathways upregulated in the EXO group, while orange bars represent downregulated pathways.

Statistics: For panel A, data are presented as mean ± SD from three technical replicates. Statistical significance was assessed using an unpaired two-tailed Student’s t-test. ***P < 0.001.

**
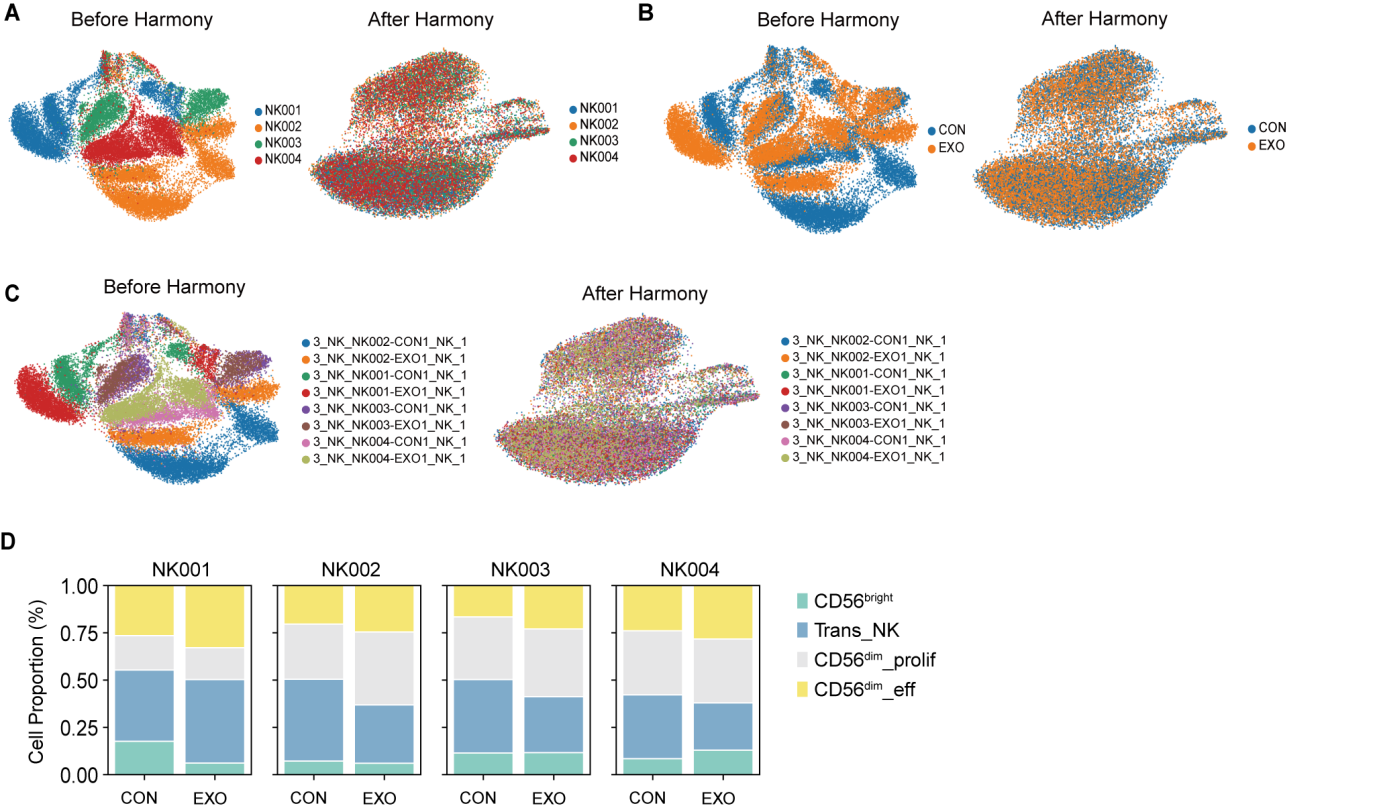
**

**Supplementary Figure S4. Harmony integration improves donor mixing while retaining condition-associated structure.**

**(A)** UMAP plots colored by donors (NK001, NK002, NK003, NK004) before and after Harmony integration, showing improved donor mixing after batch correction. **(B)** UMAP plots colored by treatment condition (CON vs EXO) before and after Harmony integration, indicating that condition-associated structure remains observable after correction. **(C)** UMAP plots colored by batch before and after Harmony integration, showing reduced batch-associated separation after correction. **(D)** Stacked bar plots showing the proportions of annotated NK cell subclusters for each donor under CON and EXO treatment conditions.


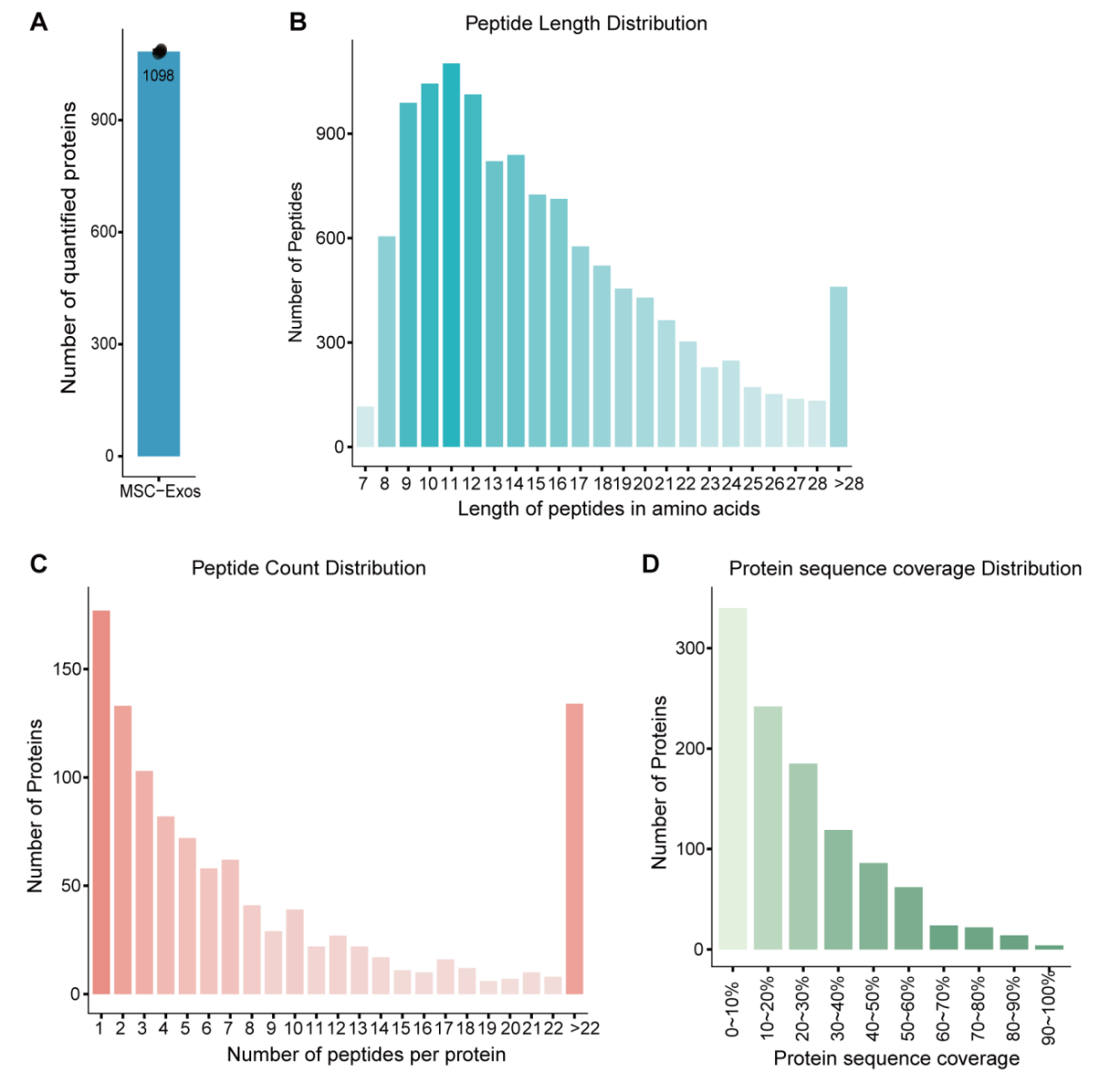


**Supplementary Figure S5. Overview of proteomic profiling and peptide features of MSC-derived exosomes.**

**(A)** Bar plot showing the total number of quantified proteins identified in MSC-Exos by LC–MS/MS. Data are shown from three technical replicates. **(B)** Length distribution of peptides identified in MSC-Exos. **(C)** Distribution of the number of unique peptides identified per protein in MSC-Exos. **(D)** Distribution of sequence coverage across proteins identified in MSC-Exos.
