# Supplementary material for: Integrative single-cell transcriptomics and proteomics reveal an immunometabolic framework for MSC-exosome-mediated remodeling of expanded NK cells
Source: Gigascience. 2026 Apr 20;15:giag049. doi: 10.1093/gigascience/giag049 (PMC13273429; doi:10.1093/gigascience/giag049)
Supplement: giag049_GIGA-D-26-00032_original_submission [file giag049_GIGA-D-26-00032_original_submission.pdf]

GigaScience

Integrative single-cell transcriptomics and MSC-exosome proteomics reveal a mechanistic basis for MSC-exosome-driven NK-cell expansion and effector reprogramming

--Manuscript Draft--

**Manuscript Number:**	GIGA-D-26-00032
**Full Title:**	Integrative single-cell transcriptomics and MSC-exosome proteomics reveal a mechanistic basis for MSC-exosome-driven NK-cell expansion and effector reprogramming
**Article Type:**	Research
**Funding Information:**	
**Abstract:**	Background<br>Natural killer (NK) cells are pivotal for anti-tumor immunity and immunosurveillance of senescence, yet their clinical performance is frequently limited by functional exhaustion during ex vivo expansion. Mesenchymal stem cell-derived exosomes (MSC-Exos) are increasingly recognized as potent immunomodulators, but their systematic effects on NK-cell fitness and functional states remain insufficiently characterized.<br>Results<br>Here, we assessed MSC-Exo-mediated regulation of human NK cells using a standardized ex vivo priming platform integrated with single-cell transcriptomics and proteomic profiling. MSC-Exos significantly accelerated NK-cell proliferation in a dose- and time-dependent manner while preserving the CD56+CD3- phenotype. Consistently, MSC-Exos enhanced cytotoxicity against K562 tumor cells and senescent fibroblasts, accompanied by increased expression of activating receptors (NKG2D, CD16), reduced LAG3 expression, and stronger granzyme B-associated degranulation. Mechanistically, MSC-Exos reinforced NRF2-linked redox homeostasis and improved mitochondrial fitness. Single-cell analyses indicated prioritized translational programs and immune-effector pathways with reduced inflammatory stress, and trajectory inference supported a shift from regulatory to cytotoxic effector states. In parallel, proteomic profiling revealed enrichment of FcγR signaling components in MSC-Exos, which supports a plausible molecular basis for enhanced CD16/FcγR-associated effector responses.<br>Conclusions<br>Together, MSC-Exos promote NK-cell expansion and effector maturation through coordinated immunometabolic reprogramming, providing a practical strategy to strengthen NK cell-based immunotherapies.
**Corresponding Author:**	Xuan DONG<br>BGI Research<br>Hangzhou, Zhejiang Province CHINA
**Corresponding Author Secondary Information:**	
**Corresponding Author's Institution:**	BGI Research
**Corresponding Author's Secondary Institution:**	
**First Author:**	Yunyun Fu
**First Author Secondary Information:**	
**Order of Authors:**	Yunyun Fu
	Yi Liu
	Mingwen Xu
	Gaojun Liu
	Jianzhi Sun

Powered by Editorial Manager® and ProduXion Manager® from Aries Systems Corporation

	Fanyu Bu
	Wenqing Xie
	Jiayi Zhao
	Jun Luo
	Qiang Guo
	Yinghua Huang
	Fengping Xu
	Siqi Liu
	Longqi Liu
	Ying Fu
	Xuan DONG
**Order of Authors Secondary Information:**	
**Additional Information:**	
**Question**	**Response**
Are you submitting this manuscript to a special series or article collection?	No
**Experimental design and statistics**<br><br>Full details of the experimental design and statistical methods used should be given in the Methods section, as detailed in our Minimum Standards Reporting Checklist. Information essential to interpreting the data presented should be made available in the figure legends.<br><br>Have you included all the information requested in your manuscript?	Yes
**Resources**<br><br>A description of all resources used, including antibodies, cell lines, animals and software tools, with enough information to allow them to be uniquely identified, should be included in the Methods section. Authors are strongly encouraged to cite Research Resource Identifiers (RRIDs) for antibodies, model organisms and tools, where possible.<br><br>Have you included the information requested as detailed in our Minimum	Yes

Powered by Editorial Manager® and ProduXion Manager® from Aries Systems Corporation

Standards Reporting Checklist?	
**Availability of data and materials**<br><br>All datasets and code on which the conclusions of the paper rely must be either included in your submission or deposited in publicly available repositories (where available and ethically appropriate), referencing such data using a unique identifier in the references and in the "Availability of Data and Materials" section of your manuscript.<br><br>Have you have met the above requirement as detailed in our Minimum Standards Reporting Checklist?	Yes
GigaScience has policies and guidelines in place for the use of generative AI-writing tools such as ChatGPT. If you have used such writing tools to assist with writing the manuscript this must be declared and cited in the text. Authors should not list AI-writing tools and other AI-assisted technologies as an author or co-author and should acknowledge that they are fully responsible for text generated or refined by AI-writing tools.<p><br><br>A summary of use (particularly in the introduction or among methods) needs to be included at the end of the paper, and the outputs should also be included as a supplementary file hosted in GigaDB or other open repositories. Please read our guidelines for more information. <p><br><br>By submitting to GigaScience, you are aware of the journal's AI-writing tools policy, and if you have declared use of such tools below, you have acknowledged this where appropriate in your manuscript and have made a summary of use and outputs available. <p> <b>AI-assisted writing tools have been used in the preparation of this manuscript?	Yes

Manuscript

Click here to access/download;Manuscript;Manuscript_gigascience_260129

Integrative single-cell transcriptomics and MSC-exosome proteomics reveal a mechanistic basis for MSC-exosome-driven NK-cell expansion and effector reprogramming

Yunyun Fu[1, 2], Yi Liu[1, 2], Mingwen Xu[2, 3], Gaojun Liu[1, 2], Jianzhi Sun[1, 2], Fanyu Bu[2], WenQing Xie[4], JiaYi Zhao[5], Jun Luo[1,6], Qiang Guo[2], Yinghua Huang[7], Fengping Xu[8], Siqi Liu[6], Longqi Liu[2], Ying Fu[2,*], Xuan Dong[2,9,*]

1. College of Life Sciences, University of Chinese Academy of Sciences, Beijing 100049, China.

2. BGI Research, Hangzhou 310030, China.

3. Key Laboratory of Systems Health Science of Zhejiang Province, School of Life Science, Hangzhou Institute for Advanced Study, University of Chinese Academy of Sciences, Hangzhou 310024, China.

4. Interdisciplinary Research Center on Biology and Chemistry, Shanghai Institute of Organic Chemistry, Chinese Academy of Sciences, Shanghai 201210, China.

5. Department of School of Life Science and Technology, China Pharmaceutical University, Nanjing 210009, China.

6. HIM-BGI Omics Center, Hangzhou Institute of Medicine (HIM), Chinese Academy of Sciences, Hangzhou 310018, China.

7. BGI Cell, Hangzhou 310030, China.

8. BGI Cell, Shenzhen 518083, China.

9. Guangdong Provincial Key Laboratory of Human Disease Genomics, BGI Research, Shenzhen 518083, China

* Authors for correspondence

Correspondence: dongxuan@genomics.cn, fuying3@genomics.cn.

Abstract

Background

Natural killer (NK) cells are pivotal for anti-tumor immunity and immunosurveillance of senescence, yet their clinical performance is frequently limited by functional exhaustion during *ex vivo* expansion. Mesenchymal stem cell-derived exosomes (MSC-Exos) are increasingly recognized as potent immunomodulators, but their systematic effects on NK-cell fitness and functional states remain insufficiently characterized.

Results

Here, we assessed MSC-Exo-mediated regulation of human NK cells using a standardized ex vivo priming platform integrated with single-cell transcriptomics and proteomic profiling. MSC-Exos significantly accelerated NK-cell proliferation in a dose- and time-dependent manner while preserving the $CD56^{+}CD3^{-}$ phenotype. Consistently, MSC-Exos enhanced cytotoxicity against K562 tumor cells and senescent fibroblasts, accompanied by increased expression of activating receptors (NKG2D, CD16), reduced LAG3 expression, and stronger granzyme B-associated degranulation. Mechanistically, MSC-Exos reinforced NRF2-linked redox homeostasis and improved mitochondrial fitness. Single-cell analyses indicated prioritized translational programs and immune-effector pathways with reduced inflammatory stress, and trajectory inference supported a shift from regulatory to cytotoxic effector states. In parallel, proteomic profiling revealed enrichment of FcγR signaling components in MSC-Exos, which supports a plausible molecular basis for enhanced CD16/FcγR-associated effector responses.

Conclusions

Together, MSC-Exos promote NK-cell expansion and effector maturation through coordinated immunometabolic reprogramming, providing a practical strategy to strengthen NK cell-based immunotherapies.

Keywords

Mesenchymal stem cell-derived exosomes, natural killer cells, immunometabolic reprogramming, FcγR signaling, FcγR-associated effector function

Background

The global population is aging rapidly, intensifying the burden of immunosenescence and increasing susceptibility to infections, malignancies, and chronic degenerative diseases. This trend underscores the need for safe, effective, and scalable immune interventions. As key components of innate immunity, natural killer (NK) cells eliminate infected or malignant cells in a non-major histocompatibility complex (non-MHC)-restricted manner and contribute to both antitumor immunity and clearance of senescent cells [1-4]. However, clinical translation typically requires *ex vivo* expansion, which often induces functional attenuation, metabolic exhaustion, and phenotypic instability, thereby limiting efficacy in adoptive cell therapy [5, 6].

Mesenchymal stem cell-derived exosomes (MSC-Exos) are potent mediators of intercellular communication, featuring low immunogenicity, high biocompatibility, and diverse bioactive cargos (proteins, lipids, and nucleic acids) [7]. Their immunomodulatory effects are context-dependent: MSC-Exos are often described as anti-inflammatory and pro-repair, for example by promoting regulatory T-cell differentiation[8] or modulating macrophage polarization [9]. Increasing evidence, however, supports a dynamic profile rather than a fixed suppressive role. This plasticity is illustrated by observations that MSCs inhibit T-cell proliferation at high co-culture ratios yet promote T-cell activation at lower ratios [10-13].

Importantly, the immunosuppressive activity of MSC-Exos in models such as graft-versus-host disease (GvHD) or systemic inflammation does not preclude their ability to enhance NK-cell activity under specific conditions. For example, bone marrow-derived MSC-Exos reportedly increase miR-1925 and enhance NK cell-mediated cytotoxicity against hepatocellular carcinoma [14]. These findings underscore the bidirectional potential of MSC-Exos, yet the global impact of MSC-Exos on NK-cell proliferation, functional states, and underlying molecular coordination remains poorly elucidated.

In this study, we established a standardized *ex vivo* NK-cell expansion and MSC-Exos intervention workflow to map how MSC-Exos reshape NK-cell proliferation, phenotypic stability, and cytotoxic programs in human peripheral blood-derived NK cells. By integrating flow cytometry, functional assays,

single-cell transcriptomics, and proteomics, we define an immunometabolic framework for exosome-mediated NK-cell activation and provide a rationale for exosome-augmented NK-cell immunotherapy.

Analyses

MSC-Exos promote NK cells expansion and phenotypic stability

To evaluate MSC-Exos effects on *ex vivo* expansion and functional states of human peripheral blood NK cells, we implemented an expansion-and-intervention workflow with multidimensional readouts (Fig. 1A). Briefly, peripheral blood mononuclear cells (PBMCs) from healthy donors were expanded for 10 days using a commercial culture system. Starting on day 10, the exosome-treated group (EXO) received MSC-Exos every three days, whereas the control group (CON) received no exosome supplementation. Flow cytometry, functional assays, and single-cell RNA sequencing (scRNA-seq), were performed on day 16.

Vesicles isolated via size-exclusion chromatography (SEC) exhibited typical exosome-like morphology under transmission electron microscopy (TEM) (Fig. 1B). Nanoparticle tracking analysis (NTA) showed a relatively homogeneous population with a size distribution peaking between 60 and 200 nm (Fig. 1C). Immunoblotting confirmed enrichment of established exosomal markers (CD63, TSG101, and Syntenin) and absence of the endoplasmic reticulum contaminant Calnexin (Fig. 1D), supporting exosome identity and purity consistent with the MISEV (Minimal Information for Studies of Extracellular Vesicles) guidelines.

We next assessed whether MSC-Exos promote NK-cell proliferation under standard expansion conditions. NK cells from multiple donors were cultured with graded doses of MSC-Exos (10, 20, and 40 μg/mL) (Supplementary Table 1). MSC-Exos significantly increased NK-cell viability (CCK8, OD450 nm) after 6 days in a dose-dependent manner, with a pronounced effect at 20 μg/mL (Fig. 1E; Supplementary Fig. 1A). Longitudinal measurements further showed accelerated expansion over time (Fig. 1F and Supplementary Fig. 1B-C), with the largest difference observed by day 16.

Importantly, flow cytometry indicated that both groups maintained high frequency of $CD3^{-}CD56^{+}$ cells (approximately 56.5%-79.0%), with no significant differences between EXO and CON (Fig. 1G and Supplementary Fig. 1D). Thus, MSC-Exos enhance NK-cell expansion without compromising the core lineage phenotypic, providing a foundation for subsequent analyses of functional potentiation and mechanism.

MSC-Exos potentiate NK cells cytotoxicity by upregulating activating receptors and optimizing mitochondrial metabolism

Having established enhanced expansion with preserved phenotype, we next examined NK-cell effector function. MSC-Exos pretreatment markedly increased NK cell-mediated lysis of K562 tumor cells (Fig. 2A and Supplementary Fig. 2D). To model a clinically relevant senescent microenvironment, we induced senescence in human dermal fibroblasts (HDFs) using doxorubicin (DOX). DOX exposure for 48 hours reduced HDF viability in a dose-dependent manner (Supplementary Fig. 2A). Treatment with 150 nM DOX for 48 hours effectively produced hallmark senescent features, including enlarged flattened morphology, increased SA-β-Gal activity (Supplementary Fig. 2B), and significant transcriptional upregulation of senescence- and inflammation-associated markers, including *TNF*, *CDKN1A*, and *IL1B* (Supplementary Fig. 2C). In cytotoxicity assays against senescent HDFs, MSC-Exos significantly enhanced NK-cell killing in multiple donors (NK001, NK002, NK004), whereas donor NK003 showed a non-significant trend (Fig. 2B and Supplementary Fig. 2E), indicating inter-donor variability in response to exosomal priming.

To identify phenotypic correlates of enhanced cytotoxicity, we profiled NK-cell receptor expression. Compared with controls, MSC-Exos increased expression of activating receptors (NKG2D, CD16, and NKG2C) and reduced the mean fluorescence intensity (MFI) of the inhibitory checkpoint LAG3 (Fig. 2C-F). At the effector level, MSC-Exos–treated NK cells displayed stronger degranulation responses, with higher CD107a mobilization and increased granzyme B secretion upon target stimulation (Fig. 2G-H).

Metabolic profiling indicated improved mitochondrial fitness following MSC-Exos treatment. MSC-Exos increased the MitoTracker Red/Green ratio and elevated mitochondrial membrane potential ($\Delta\Psi m$; JC-1 ratio), and these advantages persisted after target engagement (Fig. 2I-J). Consistently, Quantitative real-time polymerase chain reaction (qRT-PCR) showed higher NRF2 (*NFE2L2*) mRNA levels in MSC-Exos-treated NK cells (Fig. 2K), supporting activation of NRF2-associated antioxidant programs that may sustain mitochondrial homeostasis during repeated cytotoxic challenges.

Together, these data indicate that MSC-Exos enhance NK-cell antitumor and anti-senescence surveillance by strengthening activating receptor signaling, dampening inhibitory cues, and improving mitochondrial and redox homeostasis, thereby increasing cytotoxic efficiency across complementary functional readouts.

MSC-Exos enhance NK cells cytotoxic activity and alleviate stress through immunometabolic

coordination

To characterize transcriptional programs underlying MSC-Exos–mediated remodeling, we performed single-cell RNA sequencing (scRNA-seq) on NK cells from EXO and CON groups followed by pseudo-bulk analysis and subcluster annotation (Fig. 3A). A total of 28,382 NK cells were subjected to further analyses. MSC-Exos–treated NK cells showed higher expression of cytotoxic effector genes (*GZMB, FCGR3A, NKG7,* and *GNLY*), whereas pro-inflammatory cytokine transcripts (*IFNG* and *TNF*) were reduced (Fig. 3B). Concomitantly, IFN-γ protein levels were also lower (Supplementary Fig. 2A), while cytotoxic granule scores were elevated (Fig. 3C), consistent with a shift toward a more cytotoxic phenotype. In addition, MSC-Exos attenuated signatures of senescence, the senescence-associated secretory phenotype (SASP), and oxidative stress, suggesting improved functional competence with reduced stress burden (Fig. 3D).

Differential expression analysis further identified upregulation of activation-associated genes, including *FGR1*, *ISG20*, *TNFSF9*, and *MYC* (Fig. 3E). Pathway analyses highlighted increased cytoplasmic translation and type I interferon signaling (Fig. 3F), consistent with enhanced biosynthetic capacity and immune activation. Gene set enrichment analysis (GSEA) also indicated enrichment of FcγR-related processes and Rho GTPase-IQGAP signaling, together with glycolytic pathways (Fig. 3G), suggesting coordinated cytoskeletal and metabolic remodeling that supports NK-cell effector function. This interpretation was supported by increased oxidative phosphorylation and pentose phosphate pathway activity (Supplementary Fig. 3B-C), whereas decreased pyruvate and ketone body metabolism suggested altered carbon substrate utilization. Collectively, these transcriptional changes are consistent with improved energy management and redox balance, which may help limit inflammatory stress while sustaining NK-cell functional stability.

MSC-Exos orchestrate the transition of NK cells from regulatory to an effector-cytotoxic phenotype at the single-cell level

Given that MSC-Exos substantially reshaped the transcriptional programs of the expanded NK-cell products, we hypothesized that MSC-Exos might also modulate NK-cell differentiation states at the single-cell level. To test this, we performed unsupervised clustering of the integrated scRNA-seq dataset to resolve NK-cell subpopulations and to determine whether MSC-Exos bias the population toward specific functional states. Unsupervised clustering identified four NK-cell subclusters (Fig. 4A and 4C): (i) CD56bright, a

regulatory-like and metabolic fitness subset characterized by high expression of *MALAT1*, *NEAT1*, *CD247* and *MT-ATP6*; (ii) Trans_NK, a transitional or inflammatory subset enriched for *FCER1G, KLRB1, IL2RB*, and *CD44*; (iii) CD56dim_prolif, a proliferative subset marked by cell-cycle genes including *TOP2A, MKI67*, and *HIST1H2AC*; and (iv) CD56dim_eff, an effector-cytotoxic subset with elevated *NKG7*, *CD52*, and *IL32*. These subclusters were consistently detected across donors and treatment conditions (Fig. 4B), supporting robust integration and effective batch correction. MSC-Exos treatment significantly increased the proportion of CD56dim_eff cells ($p = 0.003$), whereas the fraction of CD56bright cells remained largely unchanged (Fig. 4D). This shift toward the effector-cytotoxic compartment provides a cellular basis for the enhanced cytotoxic function observed in MSC-Exos–treated NK-cell products.

To examine whether MSC-Exos influence NK-cell maturation dynamics, we reconstructed differentiation trajectories using pseudotime analysis (Fig. 4E-F). The inferred trajectory suggested a continuous progression from CD56bright to CD56dim_eff states, and the MSC-Exos group displayed a higher density of late-stage effector cells along pseudotime, consistent with accelerated functional maturation. Consistent with the trajectory analysis, MSC-Exos-treated NK cells exhibited significantly higher cytotoxicity scores in both CD56dim_prolif and CD56dim_eff subsets (Fig. 4G). Gene Ontology (GO) enrichment analysis of subset-specific differentially expressed genes further highlighted upregulation of pathways related to mitochondrial activity, protein translation, and responses to oxygen and cellular stress, accompanied by downregulation of chromatin-condensation-associated processes (Fig. 4H). Together, these findings indicate that MSC-Exos promote a metabolically active and cytotoxic effector phenotype within the CD56dim compartment.

Collectively, these results demonstrate that MSC-Exos remodel NK-cell differentiation trajectories by expanding the effector arm (CD56dim_eff) while maintaining overall phenotypic stability, thereby optimizing the balance between proliferation and cytotoxic function.

Proteomic profiling reveals enrichment of FcγR signaling modules in MSC-Exos, supporting a plausible molecular basis for enhanced CD16/FcγR-associated effector responses

To explore the molecular basis underlying MSC-Exos-mediated NK-cell activation [15], we performed quantitative proteomic profiling of MSC-Exos. A total of 1,098 proteins were identified (Supplementary Fig. 4A; Supplementary Tables 3-5). Most identified peptides ranged from 8 to 20 amino acids in length (Supplementary Fig. 4B). Notably, 83.88% of proteins were supported by two or more peptides

(Supplementary Fig. 4C), and 69% of proteins showed sequence coverage greater than 10% (Supplementary Fig. 4D), indicating high confidence in exosomal protein identification.

To connect exosomal cargos with NK-cell activation programs, we calculated Jaccard similarity between the MSC-Exos proteome and pathways upregulated in MSC-Exos-treated NK cells based on transcriptomic analysis. FcγR-dependent phagocytosis, *FCGR3A*-mediated IL-10 synthesis, and glycolysis /gluconeogenesis exhibited the highest overlap, suggesting that MSC-Exos carry signaling components relevant to FcγR-associated immune regulation and energy metabolism (Fig. 5A). Consistently, Venn analysis identified 64 proteins shared between the MSC-Exos proteome and the Reactome FcγR-dependent phagocytosis pathway (Fig. 5B). This shared set included proximal kinases (e.g., SRC and BTK), downstream signaling effectors (e.g., PLCG2 and MAPK1), critical cytoskeletal regulators (e.g., RAC1), and immunoglobulin constant-region–associated proteins (e.g., IGHG1), which collectively participate in receptor-proximal signaling and immune-synapse organization. Ranking by normalized protein abundance further placed several of these components among highly represented exosomal proteins (Fig. 5C), supporting their potential functional relevance.

Together, these proteomic signatures support a model in which MSC-Exos may act as delivery vehicles for a preconfigured FcγR-associated signaling module. By providing essential signaling hubs, MSC-Exos could lower activation thresholds and facilitate rapid synapse assembly upon antibody engagement, thereby contributing to a plausible molecular basis for enhanced CD16/FcγR-associated effector responses. Further mechanistic studies will be required to determine whether these cargos are functionally transferred into recipient NK cells and whether they are necessary for the observed enhancement of effector function.

Discussion

NK cells act as a key component of the innate immune system, but their clinical application is limited by functional decline and metabolic exhaustion during large-scale *ex vivo* expansion [16, 17]. Here, we demonstrate that MSC-Exos serve as potent bio-catalysts to resolve this bottleneck. By integrating multi-omic datasets, we propose that MSC-Exos drive a distinct state of high-efficiency and low-stress NK cells activation, fundamentally differentiating them from cells produced via traditional expansion protocols.

While the literature traditionally characterizes MSC-Exos as immunosuppressive [18, 19], our findings underscore the profound context-dependency of their action. The presence of IL-2 in our system likely

establishes a pre-activated metabolic baseline. Under these conditions, MSC-Exos may function less as inhibitors and more as metabolic facilitators, potentially supporting the upregulation of key activating receptors. Consistent with this interpretation, MSC-Exos were associated with improved NK-cell fitness during late-stage *ex vivo* expansion, including enhanced cytotoxicity against tumor and senescent targets, higher expression of activating receptors (e.g., NKG2D and CD16), increased degranulation output, and improved mitochondrial indices. Together with single-cell transcriptomic evidence showing enriched cytotoxic programs and coordinated metabolic remodeling in MSC-Exos-treated NK cells, these results suggest that MSC-Exos exposure is consistent with a high-effector, low-stress functional state rather than widespread inflammatory hyperactivation. This interpretation is consistent with emerging evidence that MSC-derived factors can, under specific priming conditions, be repurposed to support rather than suppress immune surveillance[20].

Importantly, our current data do not support a single linear mechanism; rather, they point to several converging regulatory axes that may collectively shape the observed phenotype. For instance, NRF2 acts as a central transcriptional regulator that couples redox homeostasis to mitochondrial bioenergetics [21, 22]. Its activation enhances the metabolic resilience required for NK cells to persist and function within hostile microenvironments[23]. The observed upregulation of NRF2 (*NFE2L2*), together with improved mitochondrial membrane potential, aligns with a model of strengthened redox buffering and mitochondrial homeostasis; however, causality remains to be established. Future studies should therefore directly assess NRF2 dependence using loss-of-function strategies (e.g., genetic silencing, CRISPR interference, or pharmacologic inhibition) to determine whether MSC-Exos-associated improvements in $\Delta\Psi m$, degranulation, and cytotoxic capacity are attenuated upon NRF2 blockade.

Likewise, MSC-Exos are enriched with a preassembled FcγR signaling module, encompassing proximal kinases such as SRC and BTK and downstream effectors including PLCG2, MAPK1, and RAC1[24-26]. Because CD16 (*FCGR3A*) is the dominant activating Fcγ receptor mediating antibody-dependent cellular cytotoxicity (ADCC) in human NK cells, the enrichment of FcγR-pathway components in MSC-Exos, together with increased FCGR3A (CD16) expression and FcγR-related transcriptional signatures in MSC-Exos-treated NK cells, supports the possibility that MSC-Exos may potentiate CD16-dependent antibody-triggered effector responses.However, the present study does not directly establish that ADCC is enhanced, nor does it demonstrate functional transfer and requirement of these exosomal signaling proteins in recipient

NK cells. This hypothesis can be tested by tracing exosome uptake and BTK/PLCG2/SRC localization, measuring antibody-triggered phosphorylation, and disrupting function via BTK/PLCG2 inhibitors to see if augmentation diminishes. In parallel, many reported immunomodulatory effects of MSC-Exos are mediated by exosomal microRNAs, which can reshape recipient-cell signaling and transcriptional programs [14, 27]. Therefore, future work should incorporate a dedicated assessment of the MSC-Exo microRNA cargo and its contribution to NK-cell reprogramming and potential ADCC potentiation. Finally, the donor-to-donor variability observed across functional assays highlights the need to identify baseline predictors of responsiveness (e.g., mitochondrial fitness or receptor repertoire) and to evaluate the durability of these functional gains from long-term or serial-killing assays to ultimate *in vivo* models.

Collectively, our multi-omic datasets support an integrated, testable framework in which MSC-Exos are linked to coordinated immunometabolic tuning and enhanced effector maturation, while emphasizing that definitive causal pathways and the nature of exosomal cargo action require targeted mechanistic validation.

Methods

Isolation, purification, and characterization of MSC-Exos

Human umbilical cord-derived mesenchymal stem cells (hUC-MSCs; OriCell, China) were cultured in mesenchymal stem cell growth medium (APPLIEcell, China) at 37 °C in a humidified incubator with 5% CO_2. Conditioned medium was collected and stored at −80 °C until further processing.

MSC-Exos were isolated from MSC culture supernatants using size-exclusion chromatography (SEC). A Sepharose CL-6B column (10 mL bed volume) was pre-equilibrated with phosphate-buffered saline (PBS) and stored overnight at 4 °C to stabilize the resin. Supernatants were sequentially centrifuged at 300 × g for 5 min and 3,000 × g for 5 min to remove cells and debris, followed by filtration through a 0.22 μm membrane. The filtrate was concentrated to 1 mL using a 50 kDa molecular weight cut-off ultrafiltration device (3,000 × g for 30 min) and loaded onto the CL-6B column. After loading, the column was eluted with PBS in 500 μL increments. The first 2 mL of flow-through was discarded, and subsequent fractions were collected in 2 mL volumes. Exosome-rich fractions—defined by high particle counts with low protein contamination—were pooled for downstream analyses.

Purified vesicles were characterized using orthogonal approaches. Morphology was assessed by

transmission electron microscopy (TEM), particle size distribution was measured by nanoparticle tracking analysis (NTA), and exosomal identity was confirmed by immunoblotting for positive markers (CD63, TSG101, and Syntenin) and the absence of the negative marker Calnexin.

In vitro expansion of peripheral blood–derived NK cells and intervention with MSC-derived exosomes (MSC-Exos)

All human peripheral blood mononuclear cells (PBMCs) were obtained under approval from the Drug Clinical Trial Ethics Committee of Liaocheng Second People's Hospital and BGI Research. PBMCs from healthy donors were expanded *ex vivo* for 16 days using a commercial NK-cell expansion kit (Jiake Biotechnology, China) to generate NK-cell products. Beginning on day 10 of culture, the experimental group (EXO) received MSC-Exos at final concentrations of 10-40 μg/mL in NK expansion medium. Fresh medium containing the corresponding exosome concentration was replenished every 72 h. Control cultures (CON) were maintained under identical conditions but received exosome-free medium during medium changes.

CCK8 assay for evaluating the effect of MSC-Exos on NK cells proliferation

To evaluate the effect of MSC-Exos on NK-cell proliferation, expanded cells on day 10 were seeded in triplicate into 96-well plates at 5×10^4 cells per well (100 μL total volume). Proliferation/viability was assessed at 24 h, 72 h, and 144 h after intervention. EXO wells contained NK expansion medium supplemented with MSC-Exos (10-40 μg/mL), whereas CON wells received an equal volume of exosome-free medium. Medium-only wells served as blanks.

For the 144 h time point, partial medium replacement (50%) was performed on day 13 using fresh medium containing the corresponding exosome concentration for the EXO group. At each time point, 10 μL of CCK8 reagent was added per well and incubated at 37 °C for 2 h in the dark. Absorbance was measured at 450 nm using a microplate reader. A background control consisting of medium containing 20 μg/mL exosomes without cells was included, and sample readings were corrected when the absorbance difference (ΔOD) exceeded 0.1. Absorbance was measured at 450 nm using a microplate reader. All experiments were performed in triplicate, and data are presented as mean ± SD. Relative cell viability was calculated as follows:

$$\text{Relative activity} = \frac{(OD_{sample} - OD_{blank})}{(OD_{control} - OD_{blank})}$$

Induction of senescence in human dermal fibroblasts (HDFs)

Primary human dermal fibroblasts (HDFs; Jinyuan Biotechnology, China) were cultured in

DMEM/Ham's F-12 (1:1) supplemented with 10% fetal bovine serum (FBS) and 4 mM L-glutamine at 37 °C in a humidified 5% CO_2 atmosphere. To induce senescence, confluent HDFs were treated with doxorubicin hydrochloride (DOX) for 48 h. After treatment, medium was replaced with fresh complete medium, and cells were incubated for an additional 4–6 days to allow full development of senescence-associated phenotypes. Senescence was quantified using the Senescence β-Galactosidase Staining Kit (MedChemExpress, USA) according to the manufacturer's instructions. Cultures were considered significantly senescent when more than 50% of cells were positive for β-galactosidase staining.

Quantitative real-time polymerase chain reaction (qRT-PCR)

Total RNA was extracted using the FastPure Cell/Tissue Total RNA Isolation Kit V2 (Vazyme, China). RNA concentration and purity were assessed with a NanoDrop 2000 spectrophotometer (Thermo Fisher Scientific, USA). Complementary DNA (cDNA) was synthesized from 1 µg of total RNA using the HiScript II Q RT SuperMix for qPCR kit (Vazyme, China). Quantitative PCR was performed using SYBR Green master mix (Yeasen, China) and primers (Supplementary Table 2) on a QuantStudio 5 Real-Time PCR System (Applied Biosystems, USA). Relative gene expression was calculated using the 2^(-ΔΔCt) method and normalized to *ACTB*.

In vitro cytotoxicity assay of NK cells

NK-cell cytotoxicity was assessed using the CytoTox 96® Non-Radioactive Cytotoxicity Assay (Promega, USA), which quantifies lactate dehydrogenase (LDH) release following target-cell lysis. For antitumor activity, NK cells from EXO and CON groups were co-cultured with K562 target cells at effector-to-target (E:T) ratios of 5:1 and 20:1 for 5 h. To assess senescent-cell clearance, NK cells were co-cultured with DOX-induced senescent HDFs at identical E:T ratios for 20 h. Prior to co-culture, HDFs were trypsinized and counted to ensure accurate E:T setup.

After incubation, supernatants were collected and absorbance was measured at 490 nm with a reference wavelength of 680 nm using a microplate reader. Cytotoxicity was calculated according to the manufacturer's instructions. All experiments were performed in triplicate, and data are presented as mean ± SD. NK cells-mediated cytotoxicity was calculated using the following formula:

$$\text{NK cytotoxicity (\%)} = \frac{(\text{OD}_{\text{experimental}} - \text{OD}_{\text{spontaneous target}} - \text{OD}_{\text{spontaneous effector}})}{(\text{OD}_{\text{maximum release}} - \text{OD}_{\text{spontaneous target}})} \times 100\%.$$

Flow cytometry

For cell surface staining, single-cell suspensions were incubated with fluorochrome-conjugated antibodies against CD56, CD3, CD16, LAG3, NKG2D, NKG2C, and CD107a at 4 °C for 20 min in the dark, followed by washing with FACS buffer. For intracellular staining, cells were fixed and permeabilized using the Cyto-Fast™ Fix/Perm Buffer Set (BioLegend, USA) and then incubated with antibodies against granzyme B at room temperature for 20 min in the dark. Samples were acquired on a BD FACSAria III flow cytometer and analyzed using FlowJo (v10.0).

Mitochondrial membrane potential was assessed using the JC-1 Mitochondrial Membrane Potential Assay Kit (Yeasen, China). Mitochondrial mass and activity were evaluated using MitoTracker® Green FM and MitoTracker® Red CMXRos (Yeasen, China), respectively, followed by flow cytometric detection.

Cytometric bead array (CBA) measurement of IFN-γ

MSC-Exos-treated and control NK cells were co-cultured with K562 target cells at an E:T ratio of 5:1 for 12 h. Cell suspensions were centrifuged at 500 × g for 5 min, and supernatants were collected for cytokine quantification. IFN-γ concentrations were measured using the CBA Human Soluble Protein Master Buffer Kit (BD Biosciences, USA) according to the manufacturer's instructions. Capture beads were incubated with supernatants for 1 h at room temperature in the dark, followed by addition of PE-conjugated detection reagent for 2 h to form bead–cytokine–detector complexes. Beads were washed, resuspended, acquired on a BD FACSAria III, and analyzed using FCAP Array software. Results are reported as pg/mL based on standard curves.

Single-cell RNA-seq data processing and analysis

Raw sequencing reads were filtered, demultiplexed, and aligned to the hg38 human reference genome using a custom pipeline (https://github.com/MGI-tech-bioinformatics/DNBelab_C_Series_HT_scRNA-analysis-software). Only reads aligned to annotated gene exons were counted. Potential doublets were identified and removed using DoubletFinder (v2.0.3), and ambient RNA contamination was corrected using SoupX (v1.4.8) under default parameters.

Cells were retained based on the following quality thresholds: 500-20,000 UMIs, 500-6,000 detected genes, and <10% mitochondrial gene content. Downstream analyses were performed using Scanpy (v1.8.1) in Python 3.7. After library-size normalization and log transformation, the 2,000 most variable genes were selected. UMI counts and mitochondrial percentages were regressed out, and the expression matrix was scaled. Dimensionality reduction was performed by principal component analysis (PCA), followed by batch

correction with Harmony. The top 30 Harmony-corrected principal components were used to construct a k-nearest neighbor graph (k = 15). Clustering was performed using the Leiden algorithm, and clusters were annotated based on canonical marker genes. Differentially expressed genes (DEGs) were identified using Scanpy with thresholds of $\log_2$(fold change) > 0.25 and q < 0.05.

Pseudotime trajectory analysis

To delineate the developmental hierarchy of NK cells, we subsetted NK cells from the integrated scRNA-seq dataset and reconstructed pseudotime trajectories. Using the top-ranked differentially expressed genes across the four subclusters as ordering genes, we applied Monocle 2 to infer branched trajectories and used Monocle 3 to validate the global differentiation manifold. This strategy enabled mapping of the transition from early regulatory-like states to mature effector phenotypes based on dynamic lineage-associated expression patterns.

Metabolic profiling

Metabolic pathway activity scores were computed using scMetabolic under default settings to evaluate differences in energy metabolism and biosynthetic programs between treatment groups at single-cell resolution.

Gene ontology (GO) and gene set enrichment analysis (GSEA)

GO enrichment analyses were performed in R using clusterProfiler (SCR_016884). The enrichGO function was executed with Benjamini–Hochberg correction and a false discovery rate (FDR) threshold of q < 0.01. Hallmark gene sets (h.all.v2023.1.Hs.symbols.gmt) were obtained from the Molecular Signatures Database (MSigDB). For single-sample and preranked enrichment analyses, gseapy (SCR_025803) was used in Python, with parameters set to min_size = 5, max_size = 1000, and 1000 permutations.

Exosome proteomic analysis

Purified MSC-Exos were resuspended in lysis buffer containing 8 M urea and 1× protease inhibitor cocktail. Samples were lysed by ultrasonication and centrifuged at 25,000 × g for 15 min at 4 °C to collect supernatants for protein quantification. Proteins were reduced with 10 mM dithiothreitol (DTT) at 37 °C for 30 min and alkylated with 55 mM iodoacetamide (IAM) in the dark for 45 min. Trypsin was added at a 1:50 (w/w) enzyme-to-substrate ratio and digestion was performed at 37 °C for 8 h. Peptides were purified and desalted using a C18 column, collected by centrifugation (20,000 × g, 10 min, 4 °C), and reconstituted in mobile phase A (0.1% formic acid in ultrapure water) for liquid chromatography-tandem mass spectrometry

(LC-MS/MS) analysis.

Raw mass spectrometry data were processed using Spectronaut (Biognosys) and searched against the UniProt Homo sapiens database (20,360 Swiss-Prot entries) using the directDIA+ (Deep) workflow. This library-free DIA strategy was coupled with label-free quantification (LFQ) to enable high-coverage protein identification and accurate quantification without a prebuilt spectral library, generating a global proteomic landscape and differential abundance profiles for MSC-Exos.

Statistical analysis

Data are presented as mean ± standard deviation (SD). Statistical analyses were performed using GraphPad Prism 8.3.1. For normally distributed data, comparisons were conducted using Student's t-test or one-way ANOVA followed by Tukey's or Dunnett's post hoc tests, as appropriate. For single-cell transcriptomic analyses, group comparisons were performed using the Wilcoxon rank-sum test. P values were considered significant at $*P < 0.05$, $**P < 0.01$, $***P < 0.001$, and $****P < 0.0001$.

Data Availability

The sequencing data that support the findings of this study have been deposited into China National GeneBank Sequence Archive (CNSA) [28] with accession number CNP0008912 (http://db.cngb.org/cnsa/project/CNP0008912_7abac950/reviewlink/). The mass spectrometry proteomics data have been deposited to the ProteomeXchange Consortium via the PRIDE partner repository with the dataset identifier PXD073707.

Abbreviations

ADCC: antibody-dependent cellular cytotoxicity; CBA: Cytometric Bead Array; CON: the control group; DEGs: differentially expressed genes; DOX: doxorubicin; EXO: the exosomes experimental group; GSEA: Gene set enrichment analysis; GvHD: graft-versus-host disease; HDFs: human dermal fibroblasts; LC-MS/MS: liquid chromatography-tandem mass spectrometry; MFI: mean fluorescence intensity; MISEV: Minimal Information for Studies of Extracellular Vesicle; MSC-Exos: Mesenchymal stem cell-derived exosomes; NK: Natural killer; NTA: Nanoparticle tracking analysis; non-MHC: non-major histocompatibility complex; PBMCs: peripheral blood mononuclear cells; PCA: principal component

analysis; qRT-PCR: quantitative real-time polymerase chain reaction; SASP: senescence-associated secretory phenotype; scRNA-seq: Single-cell RNA sequencing; SEC: size-exclusion chromatograph; TEM: transmission electron microscopy.

Declarations

Not applicable.

Consent for publication

Not applicable.

Competing interests

The author(s) declare that they have no competing interests.

Funding

This research received no specific grant from any funding agency in the public, commercial, or not-for-profit sectors.

Authors' contributions

YY.F.: investigation, methodology, formal analysis, validation, visualization, writing-original draft; Y.L.: methodology, formal analysis, visualization, writing-review & editing; M.X.: formal analysis, visualization; G.L.: formal analysis, visualization; J.S.: formal analysis, visualization; F.B.: formal analysis, visualization; W.X.: validation; J.Z.: validation; J.L.: validation; Q.G.: methodology; Y.H.: resources; F.X.: resources; S.L.: resources, project administration; L.L.: resources; Y.F.: supervision, writing-review & editing; X.D.: conceptualization, project administration, supervision, resources, writing-review & editing.

Acknowledgements

We would like to thank DCS Cloud (https://cloud.stomics.tech/) for providing the computational resources and software support necessary for this study.

References

1. Deng X and Terunuma H. Adoptive NK cell therapy: a potential revolutionary approach in longevity therapeutics. Immun Ageing. 2024;21 1:43. doi:10.1186/s12979-024-00451-2.
2. Sagiv A, Burton DG, Moshayev Z, Vadai E, Wensveen F, Ben-Dor S, et al. NKG2D ligands mediate immunosurveillance of senescent cells. Aging (Albany NY). 2016;8 2:328-44. doi:10.18632/aging.100897.
3. Brighton PJ, Maruyama Y, Fishwick K, Vrljicak P, Tewary S, Fujihara R, et al. Clearance of senescent decidual cells by uterine natural killer cells in cycling human endometrium. Elife. 2017;6 doi:10.7554/eLife.31274.
4. Antonangeli F, Zingoni A, Soriani A and Santoni A. Senescent cells: Living or dying is a matter of NK cells. J Leukoc Biol. 2019;105 6:1275-83. doi:10.1002/JLB.MR0718-299R.
5. Bryceson YT, March ME, Ljunggren H-G and Long EO. Synergy among receptors on resting NK cells for the activation of natural cytotoxicity and cytokine secretion. Blood. 2006;107 1:159-66. doi:10.1182/blood-2005-04-1351.
6. Myers JA and Miller JS. Exploring the NK cell platform for cancer immunotherapy. Nature Reviews Clinical Oncology. 2020;18 2:85-100. doi:10.1038/s41571-020-0426-7.
7. Zhao W, Zhang H, Liu R and Cui R. Advances in Immunomodulatory Mechanisms of Mesenchymal Stem Cells-Derived Exosome on Immune Cells in Scar Formation. International Journal of Nanomedicine. 2023;Volume 18:3643-62. doi:10.2147/ijn.S412717.
8. Misaghian A, Ghadiri AA, Asadirad A, Amirzadeh S and Amari A. The Effect of Exosomes Isolated from Poly (I:C) Treated Human Wharton's Jelly Mesenchymal Stem Cells on CD4+CD25+Foxp3+ Regulatory T Cells. Iranian Journal of Allergy, Asthma and Immunology. 2024; doi:10.18502/ijaai.v23i3.15638.
9. Arabpour M, Saghazadeh A and Rezaei N. Anti-inflammatory and M2 macrophage polarization-promoting effect of mesenchymal stem cell-derived exosomes. Int Immunopharmacol. 2021;97 doi:10.1016/j.intimp.2021.107823.

10. Tse WT, Pendleton JD, Beyer WM, Egalka MC and Guinan EC. Suppression of allogeneic T-cell proliferation by human marrow stromal cells: implications in transplantation. Transplantation. 2003;75 3:389-97. doi:10.1097/01.Tp.0000045055.63901.A9.

11. Bartholomew A, Sturgeon C, Siatskas M, Ferrer K, McIntosh K, Patil S, et al. Mesenchymal stem cells suppress lymphocyte proliferation in vitro and prolong skin graft survival in vivo. Exp Hematol. 2002;30 1:42-8. doi:10.1016/s0301-472x(01)00769-x.

12. Glennie S, Soeiro Is, Dyson PJ, Lam EWF and Dazzi F. Bone marrow mesenchymal stem cells induce division arrest anergy of activated T cells. Blood. 2005;105 7:2821-7. doi:10.1182/blood-2004-09-3696.

13. Zhou Y, Day A, Haykal S, Keating A and Waddell TK. Mesenchymal stromal cells augment CD4+ and CD8+ T-cell proliferation through a CCL2 pathway. Cytotherapy. 2013;15 10:1195-207. doi:10.1016/j.jcyt.2013.05.009.

14. Ding C, Zheng Y, Li D, Zhu M and Zhu Y. Up-Regulation of miR-1925 by Bone Marrow Mesenchymal Stem Cell (BMSC) Inhibits the Growth of Liver Cancer by Promoting the Anti-Tumor Activity of Natural Killer (NK) Cells. Journal of Biomaterials and Tissue Engineering. 2022;12 3:630-3.

15. Zhang H, Xiao X, Wang L, Shi X, Fu N, Wang S, et al. Human adipose and umbilical cord mesenchymal stem cell-derived extracellular vesicles mitigate photoaging via TIMP1/Notch1. Signal Transduct Target Ther. 2024;9 1:294. doi:10.1038/s41392-024-01993-z.

16. Szmania S, Lapteva N, Garg T, Greenway A, Lingo J, Nair B, et al. Ex Vivo–expanded Natural Killer Cells Demonstrate Robust Proliferation In Vivo in High-risk Relapsed Multiple Myeloma Patients. Journal of Immunotherapy. 2015;38 1:24-36. doi:10.1097/cji.0000000000000059.

17. Granzin M, Soltenborn S, Müller S, Kollet J, Berg M, Cerwenka A, et al. Fully automated expansion and activation of clinical-grade natural killer cells for adoptive immunotherapy. Cytotherapy. 2015;17 5:621-32. doi:10.1016/j.jcyt.2015.03.611.

18. Liu X, Wei Q, Lu L, Cui S, Ma K, Zhang W, et al. Immunomodulatory potential of mesenchymal stem cell-derived extracellular vesicles: Targeting immune cells. Front Immunol. 2023;14 doi:10.3389/fimmu.2023.1094685.

19. Fan Y, Herr F, Vernochet A, Mennesson B, Oberlin E and Durrbach A. Human Fetal Liver Mesenchymal Stem Cell-Derived Exosomes Impair Natural Killer Cell Function. Stem Cells Dev. 2019;28 1:44-55. doi:10.1089/scd.2018.0015.

20. Almutairi A, Alshehri NA, Al Subayyil A, Bahattab E, Alshabibi M, Abomaray F, et al. Human decidua basalis mesenchymal stem/stromal cells enhance anticancer properties of human natural killer cells, in vitro. Front Cell Dev Biol. 2024;12:1435484. doi:10.3389/fcell.2024.1435484.

21. Wang T, Jian Z, Baskys A, Yang J, Li J, Guo H, et al. MSC-derived exosomes protect against oxidative stress-induced skin injury via adaptive regulation of the NRF2 defense system. Biomaterials. 2020;257 doi:10.1016/j.biomaterials.2020.120264.

22. Dinkova-Kostova AT and Abramov AY. The emerging role of Nrf2 in mitochondrial function. Free Radical Biology and Medicine. 2015;88:179-88. doi:10.1016/j.freeradbiomed.2015.04.036.

23. Poznanski SM, Singh K, Ritchie TM, Aguiar JA, Fan IY, Portillo AL, et al. Metabolic flexibility determines human NK cell functional fate in the tumor microenvironment. Cell Metabolism. 2021;33 6:1205-20.e5. doi:10.1016/j.cmet.2021.03.023.

24. Nimmerjahn F and Ravetch JV. Fcγ receptors as regulators of immune responses. Nature Reviews Immunology. 2008;8 1:34-47. doi:10.1038/nri2206.

25. Capuano C, Pighi C, Battella S, De Federicis D, Galandrini R and Palmieri G. Harnessing CD16-Mediated NK Cell Functions to Enhance Therapeutic Efficacy of Tumor-Targeting mAbs. Cancers. 2021;13 10 doi:10.3390/cancers13102500.

26. Galvez-Cancino F, Simpson AP, Costoya C, Matos I, Qian D, Peggs KS, et al. Fcγ receptors and immunomodulatory antibodies in cancer. Nat Rev Cancer. 2024;24 1:51-71. doi:10.1038/s41568-023-00637-8.

27. Bi Y, Qiao X, Cai Z, Zhao H, Ye R, Liu Q, et al. Exosomal miR-302b rejuvenates aging mice by reversing the proliferative arrest of senescent cells. Cell Metab. 2025;37 2:527-41.e6. doi:10.1016/j.cmet.2024.11.013.

28. Wang W, Tan C, Li L, Li X, Zhang L, Li X, et al. The China National GeneBank Sequence Archive (CNSA) 2024 update. Horticulture Research. 2025;12 5 doi:10.1093/hr/uhaf036.

Figure legend

Figure 1: MSC-Exos promote NK-cell proliferation and maintain phenotypic stability.

(A) Experimental design and workflow. PBMCs were cultured for *ex vivo* NK expansion (Day 0-10). From Day 10 to Day 16, MSC-derived exosomes (EXO) were added every three days; controls received no exosomes (CON). End-point assays included flow cytometry, in-vitro cytotoxicity, proliferation, and scRNA-seq. **(B)** Morphology of MSC-Exos was examined by Transmission electron microscopy (TEM) (scale bar, 100 nm). **(C)** Size distribution profile of MSC-Exos detected by Nanoparticle tracking analysis (NTA). **(D)** Western blot analysis for exosomal marker proteins CD63, Syntenin, and TSG101 and cell-specific marker Calnexin. **(E)** Quantitation of NK-cell proliferation (donor NK001) detected by CCK8 assay (OD450) across 10-40 μg/mL MSC-Exos treatment. n = 3, **P < 0.01. **(F)** Quantitation of NK-cell proliferation (donor NK001) by CCK8 assay (OD450) following treatment with 20 μg/mL MSC-Exos during late-stage culture (sampling on Day 11, 13, and 16). n = 3, **P < 0.01. **(G)** Flow cytometry analysis showing comparable $CD56^{+}CD3^{-}$ NK cell frequencies between CON and EXO groups. n = 5, ns, not significant.

Figure 2: Enhancement of NK cells cytotoxic efficacy, activating receptor expression, degranulation, and mitochondrial homeostasis by MSC-Exos.

(A) Percentage of lysis of K562 tumor cells by NK cells from different donors following treatment with MSC-Exos (EXO) or control (CON). The effector-to-target (E:T) ratio was 20:1 for all assays. n = 3, *P < 0.05, ***P < 0.001, ns, not significant. **(B)** Percentage of lysis of DOX-induced senescent HDF cells by NK cells from different donors following treatment with EXO or CON. The effector-to-target (E:T) ratio was 20:1 for all assays. n = 3, *P < 0.05, **P < 0.01, ***P < 0.001, ns, not significant. **(C)** Flow cytometric analysis of the expression percentages of activating receptors CD16 on the surface of NK cells in each group. n = 3, *P < 0.05, **P < 0.01, ***P < 0.001. **(D)** Flow cytometric analysis of the expression percentages of activating receptors NKG2D on the surface of NK cells in each group. n = 3, **P < 0.01, ***P < 0.001. **(E)** Flow cytometric analysis of the expression percentages of activating receptors NKG2C on the surface of NK cells in each group. n = 3, **P < 0.01, ***P < 0.001. **(F)** Mean fluorescence intensity (MFI) of the inhibitory receptor LAG3 on the surface of NK cells across groups. n = 3, *P < 0.05, **P < 0.01, ns, not significant. **(G)** Statistical analysis of NK cells degranulation levels (percentage of $CD107a^{+}$ cells) upon stimulation with target cells (K562). n = 3, **P < 0.01. **(H)** Statistical analysis of granzyme B (GrB) secretion in NK

cells (percentage of GrB^+ cells) upon stimulation with target cells (K562). n = 3, **P < 0.01. **(I)** Quantification of mitochondrial mass in NK cells using MitoTracker Red/Green staining under both basal conditions and following stimulation with K562 cells. n = 3, **P < 0.01, ***P < 0.001. **(J)** Measurement of mitochondrial membrane potential (ΔΨm) in NK cells, expressed as the JC-1 red/green fluorescence intensity ratio under both basal conditions and following K562 cells stimulation. n = 3, *P < 0.05, ***P < 0.001. **(K)** Relative mRNA expression levels of the key antioxidant gene *NFE2L2* (NRF2) in NK cells from different donors, as determined by qRT-PCR. n = 3, ***P < 0.001.

Figure 3: Single-cell transcriptomic profiling reveals MSC-Exos-induced immunometabolic reprogramming in NK cells.

(A) Experimental workflow of single-cell RNA sequencing of NK cells treated with or without MSC-Exos. PBMCs were obtained from four independent healthy donors (NK001, NK002, NK003, NK004) expanded for 16 days, with exosomes added every three days during the final culture phase (Day 10-16). Single-cell libraries were generated from each donor for pseudo-bulk and subcluster analyses. **(B)** Heatmap showing mean expression levels of cytotoxicity-associated genes (*FCGR3A, GZMB, NKG7, GNLY, IFNG, TNF*) across CON and EXO groups. **(C)** Violin plots depicting expression scores for cytotoxic granules. ***P < 0.001. **(D)** Violin plots depicting expression scores for cellular senescence, SASP, and oxidative stress response signatures. ***P < 0.001. **(E)** Volcano plot showing DEGs between EXO and CON groups. **(F)** GO enrichment analysis of upregulated DEGs in the EXO group highlighted biological processes such as cytoplasmic translation, immune activation, and cytokine responses. **(G)** GSEA showing enrichment of Fcγ receptor-dependent phagocytosis, Rho GTPase signaling, and glycolysis pathways in EXO-treated NK cells. FDR < 0.05.

Figure 4: Single-cell transcriptomic analysis reveals MSC-Exos remodel NK cell subsets toward an effector-cytotoxic phenotype.

(A) UMAP plot showing four major NK cell subsets: Trans_NK, CD56dim_prolif, CD56dim_eff, and CD56bright. **(B)** UMAP plots showing donor distribution (left) and treatment grouping (right), confirming consistent subcluster representation across four donors and conditions (CON vs EXO). **(C)** Dot plot showing representative marker genes for inflammatory, proliferative, effector, and metabolic fitness signatures across

NK subclusters, with dot size indicating fraction of cells per group and color indicating mean expression. **(D)** Comparison of NK cell subset proportions between CON and EXO groups. **(E)** Pseudotime analysis illustrating differentiation trajectories of NK subpopulations, with black lines representing predicted lineage paths. **(F)** Density distribution of pseudotime states across subclusters in CON and EXO groups. **(G)** Violin plots showing cytotoxicity expression scores of CD56dim_prolif and CD56dim_eff subsets between CON and EXO groups. ***P < 0.001. **(H)** Gene Ontology (GO) enrichment analysis of differentially expressed genes in CD56dim_prolif and CD56dim_eff subsets.

Figure 5. Proteomic characterization of MSC-Exos and identification of FcγR-related signaling modules. **(A)** Bar plot showing Jaccard similarity between MSC-Exos proteomic pathways and NK cell upregulated pathways identified by transcriptomic analysis. **(B)** Venn diagram showing the overlap between MSC-Exos proteins and those associated with the FcγR-dependent phagocytosis pathway from the Reactome database. **(C)** Ranked normalized protein expression of MSC-Exos showing key FcγR-dependent phagocytosis pathway components (IGHG1, SRC, RAC1, PLCG2, BTK, MAPK1), suggesting a conserved signaling module potentially mediating NK cells activation.

Figure1

Click here to access/download;Figure;Figure1.pdf

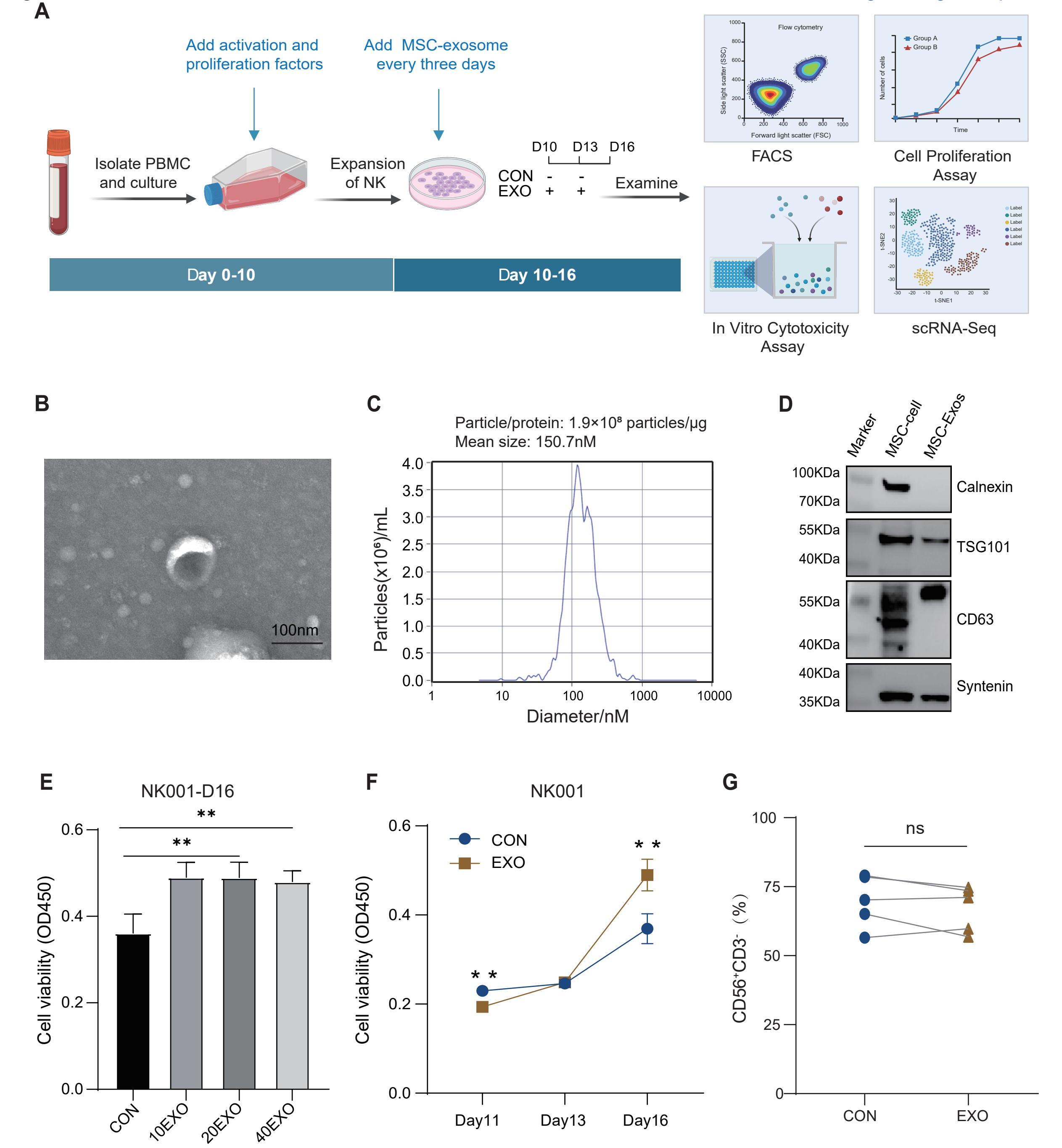

Figure2

Click here to access/download;Figure;Figure2.pdf

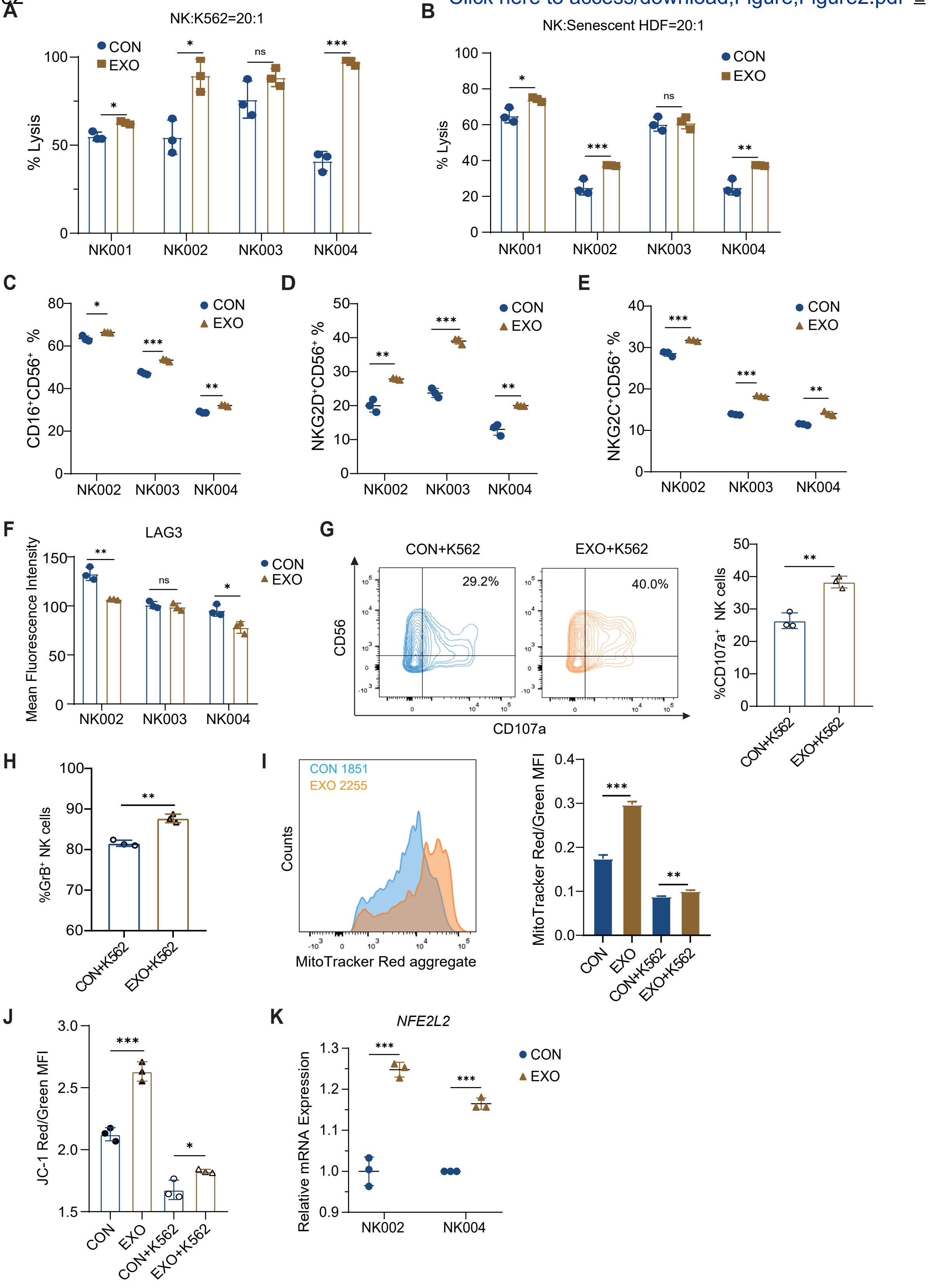

Figure3

Click here to access/download;Figure;Figure3.pdf

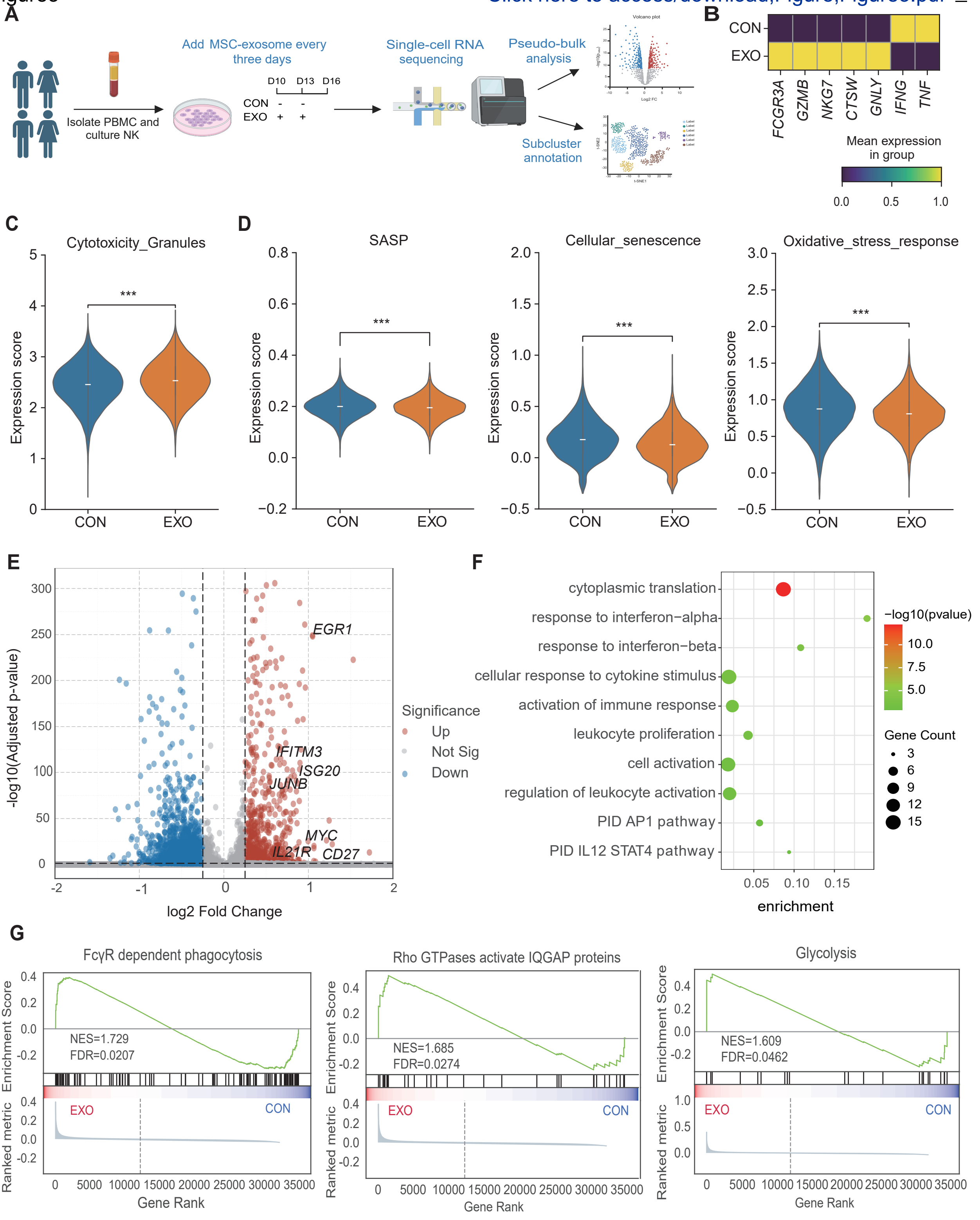

Figure4

Click here to access/download;Figure;Figure4.pdf

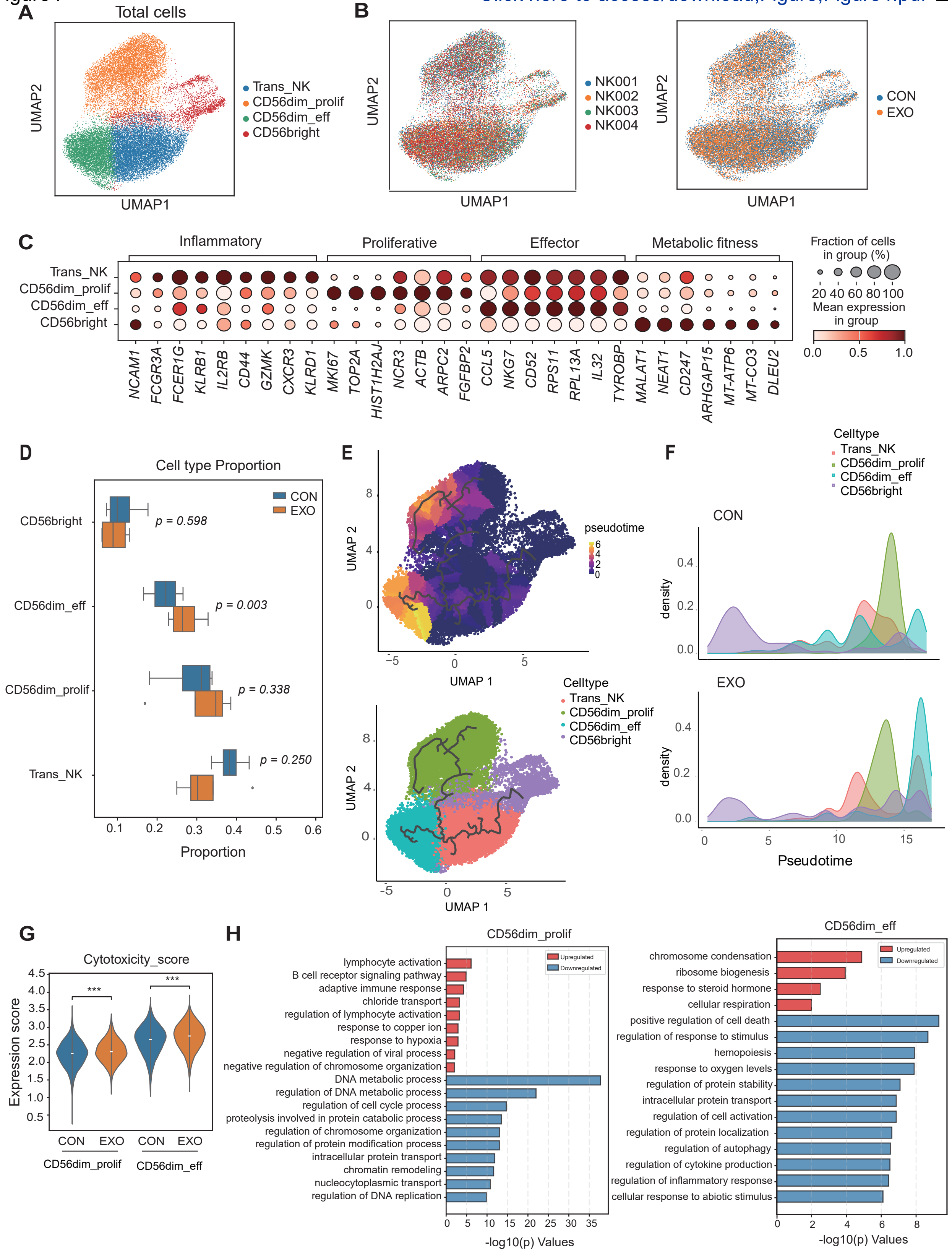

Figure5

Click here to access/download;Figure;Figure5.pdf

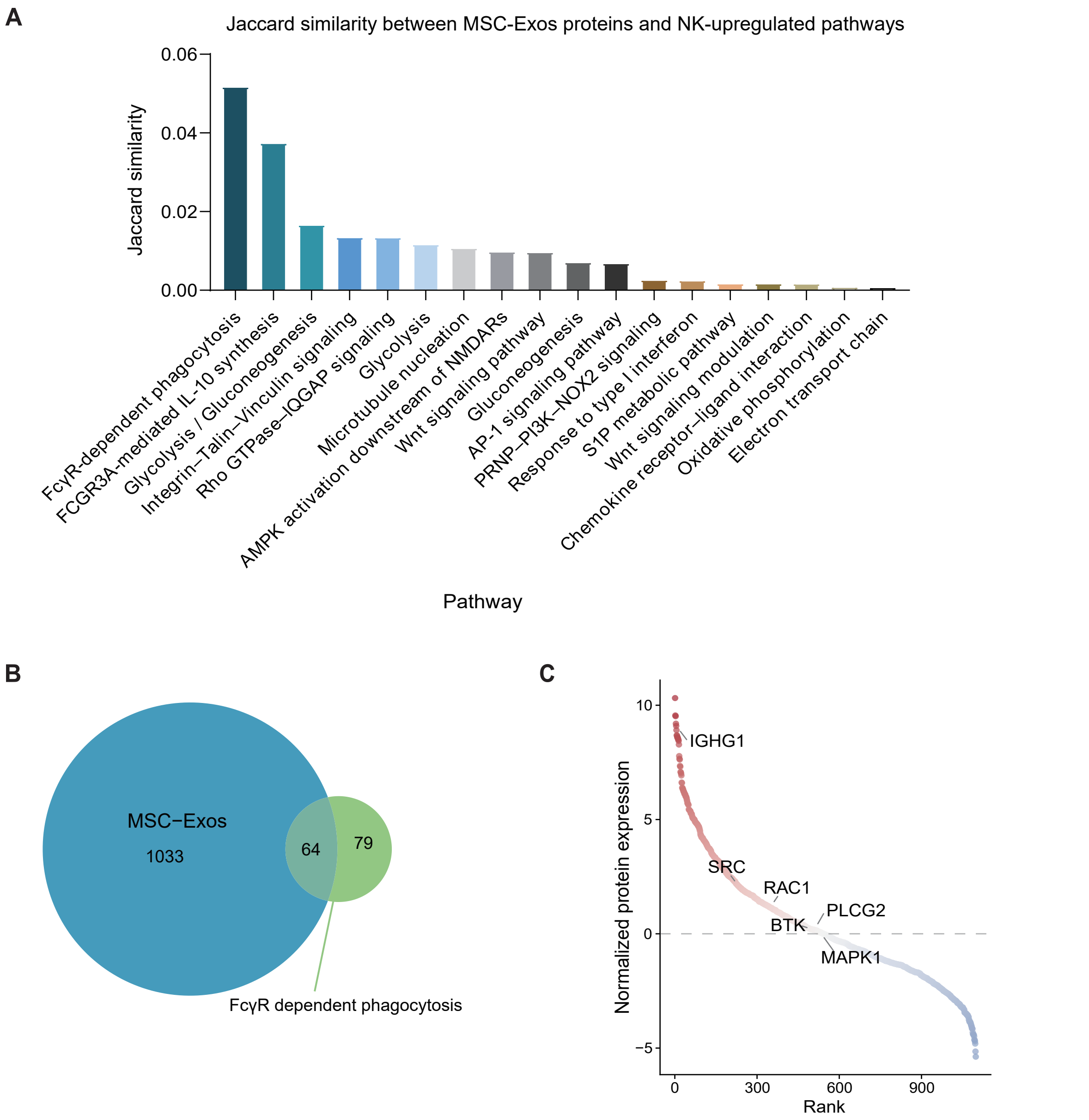

Click here to access/download
Supplementary Material
Supplementary Tables.xlsx

Click here to access/download
Supplementary Material
Supplementary Figures.docx
