# Supplementary material for: Integrative single-cell transcriptomics and proteomics reveal an immunometabolic framework for MSC-exosome-mediated remodeling of expanded NK cells
Source: Gigascience. 2026 Apr 20;15:giag049. doi: 10.1093/gigascience/giag049 (PMC13273429; doi:10.1093/gigascience/giag049)
Supplement: giag049_GIGA-D-26-00032_revision_1 [file giag049_GIGA-D-26-00032_revision_1.pdf]

GigaScience

Integrative single-cell transcriptomics and MSC-exosome proteomics reveal a mechanistic basis for MSC-exosome-driven NK-cell expansion and effector reprogramming

--Manuscript Draft--

**Manuscript Number:**	GIGA-D-26-00032R1
**Full Title:**	Integrative single-cell transcriptomics and MSC-exosome proteomics reveal a mechanistic basis for MSC-exosome-driven NK-cell expansion and effector reprogramming
**Article Type:**	Research
**Funding Information:**	
**Abstract:**	Background<br>Natural killer (NK) cells are pivotal for anti-tumor immunity and immunosurveillance of senescence, yet their clinical performance is frequently limited by functional exhaustion during ex vivo expansion. Mesenchymal stem cell-derived exosomes (MSC-Exos) are increasingly recognized as immunomodulators, but their systematic effects on NK-cell fitness and functional states remain insufficiently characterized.<br>Results<br>Here, we assessed MSC-Exo-mediated regulation of human NK cells using a standardized ex vivo priming platform integrated with single-cell transcriptomics and proteomic profiling. MSC-Exos significantly accelerated NK-cell proliferation in a dose- and time-dependent manner while preserving the CD56+CD3- phenotype. Consistently, MSC-Exos enhanced cytotoxicity against K562 tumor cells and senescent fibroblasts, accompanied by increased expression of activating receptors (NKG2D, CD16), reduced LAG3 expression, and stronger granzyme B-associated degranulation. Consistent with improved cellular fitness, MSC-Exos treatment was associated with enhanced NRF2-linked redox programs and improved mitochondrial readouts. Single-cell analyses indicated prioritized translational programs and immune-effector pathways with reduced inflammatory stress, and trajectory inference supported a shift from regulatory to cytotoxic effector states. In parallel, proteomic profiling showed enrichment of FcγR-associated signaling components in MSC-Exos, a finding that supports a plausible hypothesis linking exosomal composition with the observed enhancement of FcγR/CD16-related effector features.<br>Conclusions<br>Together, our data indicate that MSC-Exos are associated with improved NK-cell expansion and a more cytotoxic effector state through coordinated immunometabolic remodeling. These findings provide a framework and practical rationale to strengthen NK cell-based immunotherapies, while highlighting mechanistic aspects that warrant further causal validation.
**Corresponding Author:**	Xuan DONG<br>BGI Research<br>Hangzhou, Zhejiang Province CHINA
**Corresponding Author Secondary Information:**	
**Corresponding Author's Institution:**	BGI Research
**Corresponding Author's Secondary Institution:**	
**First Author:**	Yunyun Fu
**First Author Secondary Information:**	
**Order of Authors:**	Yunyun Fu
	Yi Liu
	Mingwen Xu
	Gaojun Liu
	Jianzhi Sun

Powered by Editorial Manager® and ProduXion Manager® from Aries Systems Corporation

	Fanyu Bu
	Wenqing Xie
	Jiayi Zhao
	Jun Luo
	Qiang Guo
	Yinghua Huang
	Fengping Xu
	Siqi Liu
	Longqi Liu
	Ying Fu
	Xuan DONG
**Order of Authors Secondary Information:**	
**Response to Reviewers:**	Reviewer #1:<br>1. Please clarify whether, in your scRNA-seq analysis workflow, you removed non-NK lineages (e.g., T cells) prior to downstream analyses (e.g., integration, clustering, differential expression analysis, and trajectory inference). If so, please add the corresponding procedures and rationale to the Methods section.<br>We sincerely thank the reviewer for this important point. Prior to performing NK-focused downstream analyses (integration, clustering, differential expression, and trajectory inference), we applied a stringent lineage and quality filtering strategy to minimize lineage-driven confounding and low-quality cell interference. First, major immune lineages were annotated using canonical marker sets (T cell markers: CD3D, CD3E, CD4, IL7R, CD8A, CD8B; B cell markers: CD79A, MS4A1; NK cell markers: NCAM1, KLRD1, FCGR3A, GNLY, NKG7). Non-NK-lineage cells were removed prior to constructing the NK-only dataset. In addition, we excluded low-quality or transcriptionally ambiguous clusters that lacked defining lineage markers and exhibited poor marker specificity, as these likely represented low-quality cells, multiplets, or transcriptionally uninformative populations. The resulting curated NK-only dataset was then used for integration, clustering, differential expression analysis, and trajectory inference. We have clarified this filtering strategy in both the Analyses and Methods sections to improve transparency and reproducibility.<br>We have clarified this filtering strategy in the Analyses section (Page 6, Lines 98–102) and provided the corresponding details in the Methods (Page 15, Lines 350–355).<br>2. After generating the NK-only dataset, please specify whether you recomputed the neighborhood graph and re-ran UMAP/Leiden clustering, and whether you re-ran trajectory inference on the NK-only subset (rather than reusing results obtained from the full-cell dataset).<br>We thank the reviewer for pointing out this important issue. After generating the NK-only subset, we re-performed batch correction and rebuilt the kNN neighborhood graph. We then re-ran UMAP embedding and Leiden clustering de novo on the NK-only dataset, and only thereafter proceeded with NK subset annotation and downstream comparative analyses between the MSC-Exos–treated and untreated groups. These steps have now been explicitly described in the revised Methods section. These steps have now been explicitly described in the revised Methods section (Page 15, Lines 355-–359).<br>3. Please provide stepwise cell counts for each donor×condition before and after filtering, including at minimum: (i) total cells after QC, (ii) number of cells removed (non-NK lineages), and (iii) final number of cells retained for NK-only analyses.<br>We have added a new supplementary table reporting stepwise cell counts per donor × condition, including: (i) Total cells after QC, (ii) Removed cells (non-NK lineages), and (iii) Retained cells (NK-only) for downstream analyses. We also provide an overview of cell-type composition to facilitate evaluation of potential composition shifts. We have added Supplementary Table 4 (donor-by-condition cell counts pre- and post-filtering) and clarified the lineage/quality filtering procedure in the Analyses section (Page 6, Lines 98–102) (Supplementary Table 4).<br>4. The decrease in IFNG mRNA (Fig. 3B) and IFN-γ protein (Suppl. Fig. 3A) appears at odds with enhanced effector phenotypes. Please add a focused Discussion explaining

Powered by Editorial Manager® and ProduXion Manager® from Aries Systems Corporation

that NK cytotoxic degranulation and inflammatory cytokine production can be partially uncoupled (functional skewing/uncoupling), and discuss the physiological and translational significance of this 'de-inflammatory' state (e.g., preserved killing with restrained inflammatory output).

We sincerely thank the reviewer for their insightful comment. We agree that the reduction in IFNG mRNA and IFN-γ protein may appear inconsistent with enhanced effector phenotypes. We have now added a focused paragraph in the Discussion explaining that NK cell cytotoxic degranulation and inflammatory cytokine production are partially regulated by distinct signaling modules and can be functionally uncoupled. We further discuss the physiological and translational implications of this functional skewing, highlighting that preserved cytotoxicity with restrained IFN-γ production may represent a de-inflammatory state that limits collateral tissue damage while maintaining anti-target activity. This clarification has been added to the Discussion section (Page 10, Lines 204–209) with supporting citations [28–30].

5. Enrichment of "FcγR-dependent phagocytosis" should be interpreted cautiously for NK cells. This signal likely reflects FcγR-associated cytoskeletal remodeling, immune-synapse organization, and trogocytosis/phagocytosis-like processes rather than canonical antibody-dependent cellular phagocytosis (ADCP). Please clarify this NK-specific meaning in Results/Discussion and add limiting statements in figure legends.

We agree and have revised the text to avoid implying canonical ADCP by NK cells. The relevant interpretation and limiting language have been added in the Analyses text (Page 9, Lines 176–180) and in the Figure 5B legend (Page 25, Lines 632–633).

6. Methods: please report key parameters (Harmony theta/iterations—state defaults if used; Leiden resolution; Monocle2/3 version and key settings; scMetabolic gene-set source and version).

Thank you for pointing this out. We agree that key single-cell analysis parameters should be explicitly reported for reproducibility. We have added these clarifications to the Methods section at the relevant subsections (Harmony/Leiden: Page 15, Lines 344–359; Monocle2/3: Page 15, Lines 362–370; scMetabolic: Page 16, Lines 372–374).

7. Provide UMAPs before/after Harmony correction (colored by donor/batch/condition) in the Supplementary to improve transparency of batch correction.

We sincerely thank the reviewer for this valuable suggestion. To improve transparency of batch correction, we have added Supplementary Figure 4, which visualizes UMAP embeddings before and after Harmony integration:

“(A) UMAP colored by donor (NK001–NK004) before and after Harmony, showing improved donor mixing after integration. (B) UMAP colored by condition (CON vs EXO) before and after Harmony, indicating that condition-associated structure remains observable after batch correction. (C) UMAP colored by batch before and after Harmony, showing reduced batch-associated separation after correction.”

These clarifications have been added to the Analyses section (Page 7, Lines 134–136).

8. Add a donor×condition cell-count/composition overview plot (barplot/stacked bar) to help readers assess composition shifts.

We sincerely thank the reviewer for this valuable suggestion. To facilitate assessment of donor-by-condition composition shifts, we added (i) absolute cell counts for each donor × condition in Supplementary Table 5, and (ii) a donor-stratified compositional overview in Supplementary Figure 4D, which displays the proportions of annotated NK subclusters under CON and EXO conditions.

Added figure legend (Supplementary Fig. 4D):

“(D) Stacked bar plots showing the proportions of annotated NK subclusters for each donor under CON and EXO treatment conditions.”

We have added this clarification in the Analyses section (Page 7, Lines 138–139).

9. Share version-locked analysis scripts (Scanpy, Harmony, Leiden, Monocle, scMetabolic, GSEA) or provide a private review link; list the computing environment (Python/R and package versions) in the Supplementary.

We sincerely thank the reviewer for this insightful suggestion.

To support reproducibility, we have made the scRNA-seq analysis scripts publicly available at GitHub: https://github.com/fuyunyun-95/NK-MSC-exos-scRNAseq-analysis.git. The repository contains the complete analysis code for Scanpy-based preprocessing, Harmony integration, kNN graph reconstruction, UMAP visualization,

	Leiden clustering, trajectory inference (Monocle), scMetabolic scoring, and GSEA. We also provide the software environment information in the same repository, including the Python/R versions and the versions of key packages. In the Data Availability, we revised:<br>“The scRNA-seq analysis scripts used in this study are available at GitHub (repository: https://github.com/fuyunyun-95/NK-MSC-exos-scRNAseq-analysis.git).”<br><br>Reviewer #2:<br>10. In correlating exosomal proteomic findings (e.g., enrichment of FcγR signaling modules) with NK cell functional changes, certain expressions in the current manuscript may lead readers to overinterpret the certainty of causal relationships. It is recommended to systematically enhance the prudence of wording in key sections of the manuscript (abstract, conclusions, discussion)—for instance, by revising phrases to alternatives like "supports a plausible hypothesis" or "is consistent with an enhanced state."<br>We thank the reviewer for this important suggestion. We agree that, when integrating exosome proteomics with NK-cell functional readouts, causal relationships should not be overstated. Accordingly, we systematically revised the wording in the Abstract, Conclusions, and Discussion to more clearly distinguish observations from hypotheses. Specifically, we replaced mechanistic/causal phrasing (e.g., “mechanistically,” “provide a mechanistic basis”) with more cautious language such as “is associated with,” “is consistent with,” “supports a plausible hypothesis,” and we added explicit limitation statements noting that the multi-omics concordance is hypothesis-generating and warrants further causal validation. These revisions are reflected in the Abstract (Page 2, Lines 40–46), Analyses (Page 9, Lines 174–176; Page 9, Lines 178–188), and Discussion (Page 9–10, Lines 192–203; Page 10, Lines 220–224).<br>11. Additionally, the discussion should clearly delineate direct evidence, hypotheses requiring future validation, and multiple possible interpretations, thereby precisely defining the contributions of this study and the directions for future verification.<br>We sincerely thank the reviewer for this valuable suggestion. We revised the Discussion to more clearly distinguish (i) conclusions directly supported by our functional and single-cell data, (ii) hypotheses suggested by multi-omics concordance that require causal validation, and (iii) alternative interpretations and future verification. Specifically, we now present the FcγR/CD16 “signaling readiness” concept as a plausible hypothesis (rather than a confirmed mechanism), explicitly note that enhanced ADCC and functional transfer/requirement of exosomal proteins were not directly demonstrated, and outline concrete validation experiments (ADCC assays, uptake/transfer and phosphorylation readouts, and BTK/PLCG2/SRC perturbation). We also discuss non-protein cargo (e.g., microRNAs) as an additional explanatory layer and highlight donor-to-donor variability as motivation to identify predictors and test durability in long-term, serial-killing, and in vivo models. These revisions are reflected in the Discussion (Page 10–11, Lines 220–235).<br>12. Exosome dosing should be more engineering-oriented and reproducible across batches. Reporting dose only as total protein (μg/mL) can be confounded by batch-to-batch variation in the protein-to-particle ratio. Please report in the main text and figure legends: particle concentration (particles/mL), particle-to-cell dosing (particles per cell), protein-to-particle ratio, and the rationale for SEC fraction selection.<br>We thank the reviewer for this insightful suggestion. To improve engineering-oriented reproducibility across preparations, we have revised the manuscript to report MSC-Exos dosing using both protein- and particle-based metrics. Specifically, we now provide (i) the particle-to-protein ratio measured by NTA, (ii) the corresponding particle concentration (particles/mL) for each protein dose, and (iii) the particle-to-cell dose (particles per cell) calculated using the dosing cell density, together with the reciprocal protein-to-particle ratio (μg per 1×10^10 particles) (Supplementary Table 1). In addition, we clarified the rationale for SEC fraction selection: fractions were pooled from the particle-enriched peak with minimal soluble-protein carryover (guided by a higher particle-to-protein ratio as a relative purity metric) and were subsequently validated by EV marker enrichment (CD63, TSG101, Syntenin) and depletion of the negative marker Calnexin, consistent with published SEC optimization studies and established CL-6B SEC practices.<br>We have added the NTA characterization and dosing/reproducibility reporting details to the Analyses section (Page 4, Lines 46–48, Lines 52–54) and Supplementary Table 1, and clarified the MSC-Exos isolation procedure (SEC) in the Methods (Page 11–12, Lines 256–259).

Powered by Editorial Manager® and ProduXion Manager® from Aries Systems Corporation

13. The multi-omics integration should more strictly separate pathway overlap from mechanistic delivery. Jaccard similarity and pathway overlap indicate potential concordance but do not demonstrate exosomal delivery and functional activity inside NK cells. Please harmonize phrasing across the Abstract/Conclusions/Discussion, replacing "provide a mechanistic basis/act as a delivery vehicle" with "supports a plausible hypothesis/is consistent with/suggestive of."

We thank the reviewer for this important point. This comment overlaps substantially with Reviewer Comment 10 (prudence of causal wording) and Comment 11 (clear separation of direct evidence vs hypotheses), and we have addressed these issues in a unified manner across the manuscript. Specifically, we revised the Abstract, Results/Analyses, Conclusions, and Discussion to (i) explicitly distinguish pathway overlap/Jaccard concordance from mechanistic delivery and functional activity, (ii) harmonize language to reflect the evidence strength using phrases such as “is associated with,” “is consistent with,” “supports a plausible hypothesis,” and (iii) add clear limitation statements noting that pathway overlap does not demonstrate intracellular delivery or causal necessity, which requires targeted validation. These edits are incorporated in the Abstract (Page 2, Lines 44–46), Analyses (Page 9, Lines 182–188), and Discussion (Page 10, Lines 220–228).

14. Language and terminology: refine grammar and standardize terms. For example, change line 35 "promote NK cells expansion" to "promote NK cell expansion," and line 57 "core lineage phenotypic" to "core lineage phenotype." Use a consistent term ("MSC-Exos" or "MSC-derived exosomes") and define the abbreviation at first mention.

We thank the reviewer for this helpful comment. We have thoroughly edited the manuscript to refine grammar and standardize terminology. We have updated the corresponding wording in the Analyses section (Page 4, Line 38; Page 5, Line 63–64).

15. Reproducibility details for scRNA-seq: add a supplementary table listing, per donor and condition, cell numbers pre/post filtering, Harmony parameters.

We thank the reviewer for this helpful suggestion. To improve reproducibility, we added a new supplementary table reporting stepwise cell counts per donor × condition, including (i) total cells post-QC, (ii) cells removed as non-NK lineages, and (iii) final NK-only cells retained for downstream analyses. We also summarize donor-by-condition composition to facilitate assessment of potential composition shifts. In addition, we expanded the Methods to explicitly report the Harmony parameters and downstream graph/cluster settings used for both the full dataset and the NK-only reanalysis. These updates are reflected in Supplementary Table 4 and the corresponding text in the Analyses (Page 6, Lines 98–102) and Methods (Page 15, Lines 344–345 Page 15, Lines 355–359).

16. Strength of claims: several statements are overly strong (e.g., line177 "fundamentally differentiating"). Please soften and explicitly note where causality remains to be validated.

We thank the reviewer for this important comment. We have systematically softened the wording to better align claims with the current evidence strength and explicitly noted where causality remains to be established.

These changes are reflected in the relevant sections, including the main multi-omics interpretation framework and the statements on FcγR/CD16-related enrichment (Abstract: Page 2, Lines 44–46; Discussion: Page 9, Lines 192–196; Page 10, Lines 220–228).

17. Fig. 4H GO terms are long; consider truncating/rotating/abbreviating to improve readability.

We thank the reviewer for the suggestion. To improve readability of Fig. 4H, we have truncated overly long GO term labels (with standardized shortening) and adjusted the layout (including font size/margins) to avoid label crowding. The updated Fig. 4H is shown in the revised manuscript.

Reviewer #3:

18. Please clarify whether serum-free collection conditions were used. Detection of immunoglobulin proteins (e.g., IGHG1) in exosome proteomics may reflect serum/IgG contamination, co-isolation, or surface adsorption. Please provide appropriate controls (e.g., SEC fraction selection rationale) and discuss how this affects interpretation and mechanistic inferences.

We thank the reviewer for this important quality-control and interpretation comment. We have revised the manuscript to clarify serum-free collection conditions, strengthen SEC fraction-selection rationale, and temper mechanistic inferences related to

	immunoglobulin detection. Specifically, in the Methods, we now explicitly state that hUC-MSCs were cultured in a xeno-free, serum-free medium according to the manufacturer, and that conditioned medium for MSC-Exos isolation was therefore collected under serum-free conditions. We also expanded the SEC fraction-selection rationale, specifying that exosome-rich fractions were pooled from the particle-enriched peak with minimal soluble-protein carryover, operationalized by a higher particle-to-protein ratio as a relative purity metric and further validated by MSC-Exos marker enrichment (CD63/TSG101/Syntenin) and depletion of Calnexin. In the Results/Discussion, we revised the interpretation of immunoglobulin-related proteins (e.g., IGHG1) to note that their detection may reflect co-isolation or surface adsorption rather than serum IgG carryover, and we avoid using IGHG1 as standalone evidence for functional cargo delivery. Consistent with this, we harmonized multi-omics language to emphasize that pathway overlap/proteomic enrichment is hypothesis-generating and does not demonstrate intracellular delivery or functional activity without targeted validation. These updates are reflected at the relevant locations in the manuscript (Methods: Page 11, Lines 244–247; Page 11–12, Lines 256–258; Analyses: Page 9, Lines 178–181).<br>19. Although the single-cell analysis is rich, some sections read as descriptive and could better connect to functional assays. Please add cross-level synthesis statements in the Fig. 3-4 Results/Discussion (e.g., link increased CD56dim_eff frequency with killing/degranulation; connect metabolic pathway shifts with mitochondrial readouts such as membrane potential, ROS, or respiration).<br>We thank the reviewer for this constructive suggestion. We agree that the single-cell results should be more explicitly integrated with functional and metabolic readouts. Accordingly, we revised the Fig. 2–4 Results/Discussion to add cross-level synthesis statements linking (i) scRNA-seq–defined NK states and composition shifts to cytotoxicity/degranulation assays and (ii) transcriptomic metabolic programs to mitochondrial measurements. These revisions are reflected in the Analyses (Page 7, Lines 117–120; Page 7, Lines 140–141).<br>20. NK cells are not professional phagocytes; therefore, enrichment of "FcγR-dependent phagocytosis" should be interpreted cautiously. The data are more consistent with FcγR-associated cytoskeletal remodeling, immune-synapse organization, and trogocytosis/phagocytosis-like processes rather than canonical phagocytosis. Please clarify this distinction in Analyses and Discussion.<br>We agree and have revised the text to avoid implying canonical ADCP by NK cells. We added limiting language in Results and in the corresponding figure legends. We have clarified the interpretation of the Reactome "FcγR-dependent phagocytosis" annotation in the Analyses (Page 8, Lines 168–172) and added corresponding limiting language in the Figure 5B legend (Page 25, Lines 632–633).<br>21. Potential mismatch in Supplementary figure citations: the statement "IFN-γ protein levels were also lower (Supplementary Fig. 2A)" appears inconsistent with the use of Supplementary Fig. 2A for DOX dose-response. Please verify and harmonize supplementary figure numbering.<br>Thank you for pointing out the potential mismatch in the Supplementary Figure citations. We have carefully checked and harmonized the supplementary figure numbering throughout the manuscript. The citation associated with IFN-γ protein levels has been corrected from Supplementary Fig. 2A to Supplementary Fig. 3A. The revised sentence now reads (Page 6, Lines 105): "Concomitantly, IFN-γ protein levels were also lower (Supplementary Fig. 3A)."<br>22. If exosome dose is reported as total protein (μg/mL), please also report particle concentration (particles/mL) or at least the average particle-to-protein ratio to facilitate cross-study comparison and reproducibility.<br>We thank the reviewer for this helpful suggestion. To facilitate cross-study comparison and improve reproducibility, we revised the manuscript to report MSC-Exos dosing using both protein- and particle-based metrics. Specifically, we now provide the particle-to-protein ratio measured by NTA (1.9×10^8 particles/μg), and we report the corresponding particle concentration (particles/mL) for each protein dose; we also include the reciprocal protein-to-particle ratio (52.63 μg per 1×10^10 particles) in Supplementary Table 1. We have also added the details to the Analyses (Page 4, Lines 46–48, Lines 52–54).<br>23. Specify whether n denotes biological replicates (donors) vs technical replicates, paired vs unpaired design, SD vs SEM, and ensure consistency between legends and Methods/text.<br>We thank the reviewer for this important suggestion. We have thoroughly revised the

	figure legends and the Methods to explicitly define (i) whether n refers to biological replicates (donors) or technical replicates, (ii) whether comparisons were performed using paired or unpaired designs, and (iii) whether data are presented as mean ± SD or mean ± SEM. We ensured consistency of the statistical descriptions across the figure legends, Methods, and main text, and updated the relevant legends accordingly (Figure 1–4 legends; Supplementary Figure 1–3 legends).<br>24. Supplementary Fig. 2B: consider adding quantification for β-gal staining.<br>We sincerely thank the reviewer for this valuable suggestion. We have added figure legend (Supplementary Fig. 2B):<br><br>"(B) Representative bright-field images showing morphological changes and senescence-associated β-galactosidase (SA-β-Gal) staining in human dermal fibroblasts (HDFs). HDF-CON indicates untreated control cells, and HDF-DOX represents cells treated with 150 nM doxorubicin (DOX) for 48 h. Quantification of SA-β-Gal positive cells is shown on the right. Scale bar, 200 μm."<br>These revisions are reflected in the Analyses (Page 5, Lines 72–74).
**Additional Information:**	
**Question**	**Response**
Are you submitting this manuscript to a special series or article collection?	No
**Experimental design and statistics**<br><br>Full details of the experimental design and statistical methods used should be given in the Methods section, as detailed in our Minimum Standards Reporting Checklist. Information essential to interpreting the data presented should be made available in the figure legends.<br><br>Have you included all the information requested in your manuscript?	Yes
**Resources**<br><br>A description of all resources used, including antibodies, cell lines, animals and software tools, with enough information to allow them to be uniquely identified, should be included in the Methods section. Authors are strongly encouraged to cite Research Resource Identifiers (RRIDs) for antibodies, model organisms and tools, where possible.<br><br>Have you included the information requested as detailed in our Minimum Standards Reporting Checklist?	Yes

**Availability of data and materials**<br><br>All datasets and code on which the conclusions of the paper rely must be either included in your submission or deposited in publicly available repositories (where available and ethically appropriate), referencing such data using a unique identifier in the references and in the "Availability of Data and Materials" section of your manuscript.<br><br>Have you have met the above requirement as detailed in our Minimum Standards Reporting Checklist?	Yes
GigaScience has policies and guidelines in place for the use of generative AI-writing tools such as ChatGPT. If you have used such writing tools to assist with writing the manuscript this must be declared and cited in the text. Authors should not list AI-writing tools and other AI-assisted technologies as an author or co-author and should acknowledge that they are fully responsible for text generated or refined by AI-writing tools.<p><br><br>A summary of use (particularly in the introduction or among methods) needs to be included at the end of the paper, and the outputs should also be included as a supplementary file hosted in GigaDB or other open repositories. Please read our guidelines for more information. <p><br><br>By submitting to GigaScience, you are aware of the journal's AI-writing tools policy, and if you have declared use of such tools below, you have acknowledged this where appropriate in your manuscript and have made a summary of use and outputs available. <p><br><b>AI-assisted writing tools have been used in the preparation of this manuscript?	Yes

Manuscript

Click here to access/download;Manuscript;Manuscript_gigascience_260415

Integrative single-cell transcriptomics and proteomics reveal an immunometabolic framework for MSC-exosome-mediated remodeling of expanded NK cells

Yunyun Fu[1, 2], Yi Liu[1, 2], Mingwen Xu [2, 3], Gaojun Liu[1, 2], Jianzhi Sun[1, 2], Fanyu Bu[2], Wenqing Xie [4], Jiayi Zhao [5], Jun Luo[1,6], Qiang Guo[2], Yinghua Huang[7], Fengping Xu[8], Siqi Liu[6], Longqi Liu[2], Ying Fu[2,*], Xuan Dong[2,9,*]

1. College of Life Sciences, University of Chinese Academy of Sciences, Beijing 100049, China.

2. BGI Research, Hangzhou 310030, China.

3. Key Laboratory of Systems Health Science of Zhejiang Province, School of Life Science, Hangzhou Institute for Advanced Study, University of Chinese Academy of Sciences, Hangzhou 310024, China.

4. Interdisciplinary Research Center on Biology and Chemistry, Shanghai Institute of Organic Chemistry, Chinese Academy of Sciences, Shanghai 201210, China.

5. School of Life Science and Technology, China Pharmaceutical University, Nanjing 210009, China.

6. HIM-BGI Omics Center, Hangzhou Institute of Medicine (HIM), Chinese Academy of Sciences, Hangzhou 310018, China.

7. BGI Cell, Hangzhou 310030, China.

8. BGI Cell, Shenzhen 518083, China.

9. Guangdong Provincial Key Laboratory of Human Disease Genomics, BGI Research, Shenzhen 518083, China

* Corresponding authors

Correspondence: dongxuan@genomics.cn, fuying3@genomics.cn.

ORCID IDs:

Yunyun Fu [0009-0008-8393-4429]; Yi Liu [0009-0006-9858-8798]; Ying Fu [0000-0002-3251-9849]; Xuan Dong [0000-0001-8288-322X].

Abstract

Background

Natural killer (NK) cells play a central role in anti-tumor immunity and immunosurveillance of senescence, yet their clinical performance is frequently limited by functional exhaustion during *ex vivo* expansion. Mesenchymal stem cell-derived exosomes (MSC-Exos) are increasingly recognized as immunomodulators, but their broader effects on NK cell fitness and functional states remain incompletely characterized.

Results

Here, we assessed MSC-Exos-mediated regulation of human NK cells using a standardized *ex vivo* priming platform integrated with single-cell transcriptomics and proteomic profiling. MSC-Exos significantly improved NK cell viability in a dose- and time-dependent manner while preserving a $CD56^{+}CD3^{-}$ NK-cell-enriched phenotype. MSC-Exos-treated NK cells showed enhanced cytotoxicity against K562 tumor cells and senescent fibroblasts. This phenotype was accompanied by increased expression of the activating receptors NKG2D and CD16, reduced LAG3 expression, and enhanced granzyme B expression and degranulation. Consistent with improved NK cell fitness, MSC-Exos treatment was also associated with upregulated expression of genes involved in NRF2-linked redox programs and improved mitochondrial readouts in NK cells. Single-cell analyses of MSC-Exos-treated NK cells revealed enhanced immune-effector programs and reduced inflammatory stress, while trajectory inference indicated that MSC-Exos may bias the NK cell state distribution toward more cytotoxic effector-like states. Proteomic profiling of MSC-Exos identified enrichment of FcγR-associated signaling components, supporting the hypothesis that exosomal composition may be related to the FcγR/CD16-associated transcriptional and phenotypic features observed in MSC-Exos-treated NK cells.

Conclusions

Our data indicate that MSC-Exos improve NK cell viability and functional fitness during *ex vivo* expansion and bias NK cells toward a more effector-cytotoxic state. Together, these findings provide an immunometabolic framework for MSC-Exos-assisted NK cell manufacturing, while underscoring the need for further causal validation.

Keywords

Mesenchymal stem cell-derived exosomes, natural killer cells, single-cell transcriptomics, effector-cytotoxic state, immunometabolic framework

Background

The global population is aging rapidly, intensifying the burden of immunosenescence and increasing susceptibility to infections, malignancies, and chronic degenerative diseases. This trend underscores the need for safe, effective, and scalable immune interventions. As key components of innate immunity, natural killer (NK) cells eliminate infected or malignant cells in a non-major histocompatibility complex (non-MHC)-restricted manner and contribute to both antitumor immunity and clearance of senescent cells [1-4]. However, clinical translation typically requires *ex vivo* expansion, which often induces functional attenuation, metabolic exhaustion, and phenotypic instability, thereby limiting efficacy in adoptive cell therapy [5, 6].

Mesenchymal stem cell-derived exosomes (MSC-Exos) are nanoscale extracellular vesicles secreted by mesenchymal stem cells (MSCs). They mediate intercellular communication by delivering diverse bioactive cargo, including proteins, lipids, and nucleic acids. With their low immunogenicity and high biocompatibility, MSC-Exos have emerged as important regulators of tissue repair, immune modulation, and tumor microenvironment remodeling [7, 8].

Notably, the immunomodulatory effects of MSC-Exos are context dependent rather than fixed. Although MSC-derived signals are generally associated with immunosuppressive and anti-inflammatory effects in graft-versus-host disease (GvHD) and systemic inflammation models, MSCs can exert divergent effects on T-cell proliferation depending on cell ratios and culture conditions [9-12], indicating marked functional plasticity of MSC-derived regulatory cues. This plasticity suggests that MSC-Exos may not be limited to immune suppression, but may also support effector immune-cell function under defined conditions. In parallel, MSC-Exos have been implicated in immune regulation through effects on regulatory T-cell differentiation [13], macrophage polarization [14], and directly activate pro-regenerative signaling pathways to promote regeneration [15, 16].

Recently, the potential of MSC-Exos to enhance innate immune cell function under specific conditions has garnered increasing attention. For instance, bone marrow-derived MSC-Exos can augment NK cell cytotoxicity against hepatocellular carcinoma cells [17], and a positive feedback regulatory loop between MSCs and NK cells may improve impaired NK cell function in severe pathological states [18]. Nevertheless, systematic investigations into how MSC-Exos influence NK cell viability, phenotypic state, and function during *ex vivo* expansion, as well as the underlying molecular coordination, remain limited.

Single-cell approaches are well suited to resolve hierarchical immune substructures and state transitions within heterogeneous populations, providing a high-resolution context for interpreting multi-omic changes [19, 20]. In this study, we established a standardized *ex vivo* workflow to investigate how MSC-Exos modulate expanding human peripheral blood-derived NK cells. Specifically, we examined their effects on cell viability, functional state, and cytotoxic programs, while assessing the preservation of core lineage features. By integrating flow cytometry, functional assays, single-cell transcriptomics, and proteomics, we aimed to define an immunometabolic framework for MSC-Exos-mediated NK cell remodeling and to provide a rationale for MSC-Exos-augmented NK cell immunotherapy.

Results

MSC-Exos improve NK cell viability during *ex vivo* expansion while preserving an NK-cell-enriched phenotype

To evaluate the effects of MSC-Exos on NK cell viability and functional state during *ex vivo* expansion, we established an expansion-and-intervention workflow with multidimensional readouts (**Fig. 1A**). Briefly, peripheral blood mononuclear cells (PBMCs) from healthy donors were cultured and expanded for 10 days using a commercially available, standardized culture system to generate NK cells. Starting on day 10, NK cells in the MSC-Exos-treated group (EXO) received MSC-Exos every three days, whereas those in the control group (CON) received no exosome supplementation. On day 16, NK cells from both the CON and EXO groups were subjected to flow cytometry, functional assays, and single-cell RNA sequencing (scRNA-seq).

Prior to subsequent intervention experiments, the isolated MSC-Exos were systematically characterized with respect to their morphology, size distribution, and marker protein expression. Vesicles isolated from MSC-conditioned medium by size-exclusion chromatography (SEC) exhibited typical exosome-like morphology under transmission electron microscopy (TEM) (**Fig. 1B**). These vesicles were further characterized by nanoparticle tracking analysis (NTA), which revealed a relatively homogeneous particle population with a mean diameter of 150.7 nm; most particles ranged from 60 to 200 nm, with a particle-to-protein ratio of 1.9×10^8 particles/µg (**Fig. 1C**). Immunoblotting analysis of these vesicles confirmed enrichment of established exosomal markers (CD63, TSG101, and Syntenin) and the absence of the endoplasmic reticulum contaminant Calnexin (**Fig. 1D**), supporting their exosomal identity and purity in

accordance with the Minimal Information for Studies of Extracellular Vesicles (MISEV) guidelines. To facilitate cross-preparation standardization, MSC-Exos input was quantified using protein-, particle-, and cell-normalized dosing metrics (**Supplementary Table S1**). Specifically, a dose of 20 μg/mL corresponded to 3.8×10^9 particles/mL based on the measured particle-to-protein ratio of 1.9×10^8 particles/μg. Under the culture condition used here (1.5×10^6 cells/mL), this corresponded to an estimated exposure of 2,530 particles per cell, with a protein-to-particle ratio of 52.63 μg per 1×10^{10} particles.

We next evaluated whether MSC-Exos improve NK cell viability under standard expansion conditions by comparing NK cells from multiple donors in the CON and EXO groups, with the EXO group tested across graded MSC-Exos doses of 10, 20, and 40 μg/mL (**Supplementary Tables S1 and S2**). After 6 days of MSC-Exos treatment, corresponding to day 16 of culture, NK cell viability was significantly enhanced in a dose-dependent manner, as measured by the Cell Counting Kit-8 (CCK-8) assay with optical density (OD) read at 450 nm, with the greatest effect observed at 20 μg/mL (**Fig. 1E; Supplementary Fig. S1A**). Longitudinal analysis further showed that this pro-viability effect increased over time and was most pronounced on day 16 (**Fig. 1F and Supplementary Fig. S1B–C**).

Flow cytometry indicated that both groups maintained a high frequency of $CD56^+CD3^-$ cells (approximately 56.5%–79.0%), with no significant differences between CON and EXO (**Fig. 1G and Supplementary Fig. S1D**). Thus, MSC-Exos improve NK cell viability during *ex vivo* expansion without compromising NK-lineage identity, providing a foundation for subsequent analyses of functional potentiation and mechanism.

MSC-Exos enhance NK cell cytotoxicity with increased activating-receptor expression and improved mitochondrial fitness

Having established that MSC-Exos improve NK cell viability during *ex vivo* expansion while maintaining a $CD56^+CD3^-$ NK-cell-enriched phenotype, we next examined NK cell effector function. MSC-Exos pretreatment markedly increased NK-cell-mediated lysis of K562 tumor cells (**Fig. 2A and Supplementary Fig. S2D**). To model a senescence-relevant *in vitro* context, we induced senescence in human dermal fibroblasts (HDFs) using doxorubicin (DOX). DOX exposure for 48 h reduced HDF viability in a dose-dependent manner (**Supplementary Fig. S2A**). Treatment with 150 nM DOX for 48 h robustly induced hallmark senescent features in HDFs, characterized by an enlarged, flattened morphology, a significant

increase in senescence-associated β-galactosidase (SA-β-Gal) positivity (**Supplementary Fig. S2B**), and transcriptional upregulation of senescence- and inflammation-associated markers, including *TNF, CDKN1A*, and *IL1B* (**Supplementary Fig. S2C**). In cytotoxicity assays against senescent HDFs, MSC-Exos significantly enhanced NK cell killing in multiple donors (NK001, NK002, NK004), whereas donor NK003 showed a non-significant trend (**Fig. 2B and Supplementary Fig. S2E**), indicating inter-donor variability in response to exosomal priming.

To identify phenotypic correlates of enhanced cytotoxicity, we profiled NK cell receptor expression. Compared with controls, NK cells in the EXO group exhibited increased expression of the activating receptors NKG2D, CD16, and NKG2C, together with reduced mean fluorescence intensity (MFI) of the inhibitory checkpoint LAG3 (**Fig. 2C–F**). At the effector level, MSC-Exos-treated NK cells displayed stronger degranulation responses, with higher CD107a mobilization and increased granzyme B expression upon target stimulation (**Fig. 2G–H**).

Given that sustained cytotoxic function depends on adequate metabolic support [21, 22], we next examined whether MSC-Exos also improved mitochondrial fitness in NK cells. NK cells in the EXO group showed an increased MitoTracker Red/Green ratio and elevated mitochondrial membrane potential ($\Delta\Psi m$; JC-1 ratio), and these advantages were maintained after exposure to target tumor cells (**Fig. 2I–J**). Consistently, quantitative real-time polymerase chain reaction (qRT-PCR) showed higher NRF2 (*NFE2L2*) mRNA levels in MSC-Exos-treated NK cells (**Fig. 2K**), consistent with the engagement of NRF2-associated antioxidant programs that may help sustain mitochondrial homeostasis during repeated cytotoxic challenges [22].

Together, these findings support an association between MSC-Exos treatment and enhanced NK cell cytotoxicity against both tumor and senescent targets, accompanied by increased activating receptor expression, reduced inhibitory features, and improved mitochondrial fitness linked to redox-supportive programs. Collectively, these changes correspond to greater cytotoxic efficiency across multiple complementary functional readouts.

MSC-Exos promote cytotoxic transcriptional programs while reducing stress-related signatures in NK cells

To characterize transcriptional programs underlying MSC-Exos-mediated remodeling, we performed scRNA-seq on NK cells from CON and EXO groups followed by group-level differential expression (DE) analysis and subcluster annotation (**Fig. 3A**). To minimize lineage contamination and transcriptional ambiguity, major immune lineages were annotated using canonical marker sets, and non-NK-lineage cells were removed prior to constructing the NK-cell-only dataset for downstream analyses. Clusters lacking defining NK cell markers and exhibiting poor marker specificity were further excluded to ensure high-quality lineage-restricted analyses (**Supplementary Table S4**). A total of 28,382 NK cells were retained for downstream analyses. Compared with controls, NK cells in the EXO group showed higher expression of cytotoxic effector genes, including *GZMB, FCGR3A, NKG7, CTSW*, and *GNLY*, whereas pro-inflammatory cytokine transcripts such as *IFNG* and *TNF* were reduced (**Fig. 3B**). Concomitantly, the EXO group exhibited lower IFN-γ protein levels (**Supplementary Fig. S3A**) alongside higher cytotoxic granule scores (**Fig. 3C**), consistent with features of a more cytotoxic phenotype. In addition, NK cells in the EXO group showed attenuated expression of genes associated with the senescence-associated secretory phenotype (SASP), cellular senescence, and oxidative stress responses, suggesting a reduced cellular stress burden and improved functional competence (**Fig. 3D**).

Differential expression analysis further showed that NK cells in the EXO group upregulated activation-associated genes, including *EGR1, ISG20, JUNB*, and *MYC* (**Fig. 3E**). Consistent with these transcriptional changes, pathway analyses showed that NK cells in the EXO group were enriched for cytoplasmic translation and type I interferon signaling pathways (**Fig. 3F**), in line with enhanced biosynthetic capacity and immune activation. In this context, enrichment of type I interferon signaling likely reflects antiviral and activation-associated transcriptional circuitry rather than a direct increase in IFN-γ output. Gene set enrichment analysis (GSEA) further showed enrichment of FcγR-related processes, Rho GTPase-IQGAP signaling, and glycolytic pathways in the EXO group (Fig. 3G), suggesting coordinated cytoskeletal and metabolic remodeling associated with enhanced NK cell effector function. This interpretation was further supported by metabolic features of NK cells in the EXO group, including increased oxidative phosphorylation and pentose phosphate pathway activity, whereas decreased pyruvate and ketone body metabolism suggested altered carbon substrate utilization (**Supplementary Fig. S3B–C**). These transcriptomic shifts were concordant with the improved mitochondrial fitness and increased NRF2 (*NFE2L2*) expression observed in

EXO-group NK cells (**Fig. 2I–K**), together indicating enhanced energy management and redox buffering that may help restrain inflammatory stress while maintaining functional stability.

MSC-Exos are associated with a shift in NK cell states toward effector-cytotoxic phenotypes at the single-cell level

Given that MSC-Exos substantially reshaped the transcriptional programs of the expanded NK cell products, we hypothesized that MSC-Exos might also modulate NK cell differentiation states at the single-cell level. To test this, we performed unsupervised clustering of the integrated scRNA-seq dataset to resolve NK cell subpopulations and to determine whether MSC-Exos bias the population toward specific functional states. Unsupervised clustering identified four NK cell subclusters (**Fig. 4A and 4C**): (i) CD56^{bright}, a regulatory-leaning subset with enhanced metabolic fitness features, characterized by high expression of *MALAT1, NEAT1, CD247* and *MT-ATP6*; (ii) Trans_NK, a transitional or inflammatory subset enriched for *FCER1G, KLRB1, IL2RB* and *CD44*; (iii) CD56^{dim}_prolif, a proliferative subset marked by cell-cycle-associated genes, including *MKI67, TOP2A* and *HIST1H2AC*; and (iv) CD56^{dim}_eff, an effector-cytotoxic subset with elevated expression of *NKG7, CD52*, and *IL32*. These subclusters were consistently detected across donors and treatment conditions (**Fig. 4B**), supporting robust integration and effective batch correction. This was further supported by UMAP visualizations before and after Harmony correction, which showed improved donor mixing while preserving the overall NK cell subcluster architecture under both CON and EXO conditions (**Supplementary Fig. S4A–C**).

MSC-Exos treatment significantly increased the proportion of CD56^{dim}_eff cells ($p = 0.003$), whereas the fraction of CD56^{bright} cells remained largely unchanged (**Fig. 4D**). Donor-stratified analyses further indicated that the EXO-associated enrichment of CD56^{dim}_eff cells was not driven by a single donor (**Supplementary Fig. S4D and Supplementary Table S5**). This shift toward the effector-cytotoxic compartment provides a cellular basis for the enhanced cytotoxic function observed in EXO-group NK cell products.

To examine whether MSC-Exos influence NK cell maturation dynamics, we reconstructed differentiation trajectories using pseudotime analysis (**Fig. 4E–F**). Trajectory inference suggested a shift from CD56^{bright} to CD56^{dim}_eff states, with the EXO group showing a higher density of later effector-like cells along pseudotime. Supporting this observation, NK cells in the EXO group exhibited significantly increased cytotoxicity scores in both the CD56^{dim}_prolif and CD56^{dim}_eff subsets (**Fig. 4G**). Gene Ontology (GO)

enrichment analysis of subset-specific differentially expressed genes further revealed upregulation of pathways associated with lymphocyte activation, ribosome biogenesis, and cellular respiration in NK cells from the EXO group **(Fig. 4H)**, supporting enhanced effector function and metabolic activity in these NK cell subsets. These findings suggest that MSC-Exos favor functional maturation of the CD56^{dim} NK cell compartment toward a more metabolically active and cytotoxic state.

Collectively, these results indicate that MSC-Exos remodel NK cell differentiation trajectories while preserving NK-lineage identity, thereby enhancing the balance between NK cell fitness and cytotoxic function.

Proteomic profiling identifies FcγR-associated signaling components in MSC-Exos and supports a hypothesis of FcγR/CD16-related priming

To explore the molecular features of MSC-Exos that may relate to NK cell activation programs [23], we performed quantitative proteomic profiling of MSC-Exos. In total, 1,098 proteins were identified in MSC-Exos (**Supplementary Fig. S5A; Supplementary Tables S6–S8**). Most identified peptides ranged from 8 to 20 amino acids in length (**Supplementary Fig. S5B**). Notably, 83.88% of proteins were supported by two or more peptides (**Supplementary Fig. S5C**), and 69% of proteins showed sequence coverage greater than 10% (**Supplementary Fig. S5D**), indicating high confidence in MSC-Exos protein identification.

To relate exosomal protein composition to transcriptional programs upregulated in MSC-Exos-treated NK cells, we calculated the Jaccard similarity between gene sets represented by proteins identified in the MSC-Exos proteome and gene sets from pathways upregulated in NK cells by transcriptomic analysis. Among the top-overlapping Reactome terms were FcγR-dependent phagocytosis, *FCGR3A*-mediated IL-10 synthesis, and glycolysis/gluconeogenesis (**Fig. 5A**). As NK cells are not professional phagocytes, we interpret the Reactome annotation "FcγR-dependent phagocytosis" in an NK cell context, where it more likely reflects FcγRIIIa/CD16-linked programs involving cytoskeletal remodeling, immune-synapse organization, membrane trafficking, and trogocytosis-like membrane transfer during Fc-mediated engagement, rather than canonical antibody-dependent cellular phagocytosis (ADCP). Venn analysis identified 64 proteins shared between the MSC-Exos proteome and the Reactome FcγR-dependent phagocytosis pathway gene set (**Fig. 5B**). This overlap included proximal kinases (e.g., SRC, BTK), downstream effectors (e.g., PLCG2, MAPK1), and cytoskeletal regulators (e.g., RAC1), which were involved in receptor-proximal signaling and

immune-synapse organization [24-26]. Ranking by normalized protein abundance showed that several of these components were among the more abundant proteins detected in MSC-Exos (**Fig. 5C**), supporting their potential relevance to FcγR-associated signaling biology. We note that immunoglobulin-related proteins (e.g., IGHG1) detected in exosome proteomics might reflect co-isolation with MSC-Exos-associated proteins or surface adsorption during processing, rather than serum IgG carryover; therefore, their presence is interpreted with caution and is not used as standalone evidence for functional cargo delivery.

Together, these proteomic signatures are consistent with and support the hypothesis that MSC-Exos contain FcγR/CD16-associated signaling components that may be linked to a primed signaling state in NK cells. However, pathway overlap and proteomic enrichment by themselves do not establish functional cargo delivery to NK cells, nor do they constitute evidence of enhanced antibody-dependent cellular cytotoxicity (ADCC). Future mechanistic studies, including uptake and transfer assays, functional blockade, and targeted perturbation, will be required to test whether specific exosomal components are transferred to NK cells and whether they are necessary for the enhanced FcγR/CD16-associated transcriptional and phenotypic features observed in our system.

Discussion

NK cells act as a key component of the innate immune system, but their clinical application is limited by functional decline and metabolic exhaustion during large-scale *ex vivo* expansion [27, 28]. In this study, MSC-Exos applied during *ex vivo* NK cell expansion were associated with improved NK cell fitness and a cytotoxicity-biased functional profile. Our data support a framework in which MSC-Exos may contribute to an efficient, lower-stress NK cell state, while the underlying causal mechanisms remain to be validated.

MSC-Exos are often described as immunosuppressive in other settings [29, 30], underscoring the context-dependent nature of their effects. In our expansion setting, baseline cytokine support and culture conditions may establish a pre-activated state that shifts MSC-Exos function toward supporting metabolic adaptation and effector readiness rather than broadly dampening immune activity. Importantly, the phenotype observed in *ex vivo*-expanded NK cells was not one of generalized inflammatory amplification. Instead, it is consistent with functional skewing in which cytotoxic output is enhanced while inflammatory cytokine production is restrained. This interpretation is consistent with emerging evidence that MSC-derived factors can, under specific priming conditions, be repurposed to support rather than suppress immune surveillance [31].

Notably, IFNG mRNA and IFN-γ protein were reduced despite increased degranulation and activating-receptor expression; this pattern is compatible with partial uncoupling of cytotoxicity and cytokine programs, which are governed by overlapping but distinct regulatory circuits[32-34]. Such a cytotoxic state with restrained inflammatory output may be advantageous for therapeutic manufacturing, as it preserves killing capacity while potentially limiting cytokine-associated toxicity.

At the mechanistic level, our data implicate multiple convergent regulatory axes that could collectively influence NK cell behavior, although the current evidence is primarily correlative. One plausible axis involves redox control and mitochondrial homeostasis. NRF2 is a central transcriptional regulator that couples antioxidant programs to mitochondrial resilience [35, 36] and has been linked to the metabolic robustness required for NK cell persistence and function in stressful microenvironments [22]. In our results, increased NRF2 mRNA expression together with improved mitochondrial readouts was consistent with enhanced stress-buffering capacity in MSC-Exos-treated NK cells. Future work should therefore directly assess NRF2 dependence using loss-of-function strategies (e.g., genetic silencing, CRISPR interference, or pharmacologic inhibition) to determine whether the MSC-Exos-associated enhancement of mitochondrial membrane potential, degranulation, and cytotoxic capacity is attenuated by NRF2 blockade.

A second candidate axis involves FcγR/CD16-related signaling readiness. Concordance between MSC-Exos proteomics and NK-cell transcriptomic changes suggests enrichment of FcγR-associated components and CD16-related programs, which is consistent with a plausible hypothesis that MSC-Exos exposure may facilitate FcγR/CD16-linked activation states. However, this study does not directly demonstrate enhanced ADCC, nor does it establish functional transfer and requirement of specific exosomal proteins within recipient NK cells. These possibilities could be tested by (i) directly measuring antibody-triggered ADCC in standardized assays, (ii) tracing exosome uptake and assessing receptor-proximal signaling events (e.g., phosphorylation of FcγR pathway nodes), and (iii) evaluating sensitivity to pathway inhibition (e.g., BTK/PLCG2/SRC perturbation) to determine whether any observed augmentation depends on these components.

Beyond proteins, MSC-Exos may also exert effects through non-protein cargo such as microRNAs, which can reprogram signaling and transcriptional states in recipient cells [17, 37]. Dedicated profiling and functional interrogation of MSC-Exo microRNA cargo will therefore be important to define the contributions of distinct cargo classes. Finally, the limited number of donors and the evident inter-donor variability across

functional readouts should be recognized as important limitations of this study. While MSC-Exos-associated functional benefits were observed across donors, the magnitude of these effects was variable, highlighting the need to identify baseline predictors of responsiveness, such as mitochondrial fitness or activating-receptor repertoire. Future work in larger donor cohorts should further define these predictors and evaluate the durability of MSC-Exos-associated gains through long-term persistence studies, serial-killing assays, and ultimately *in vivo* models.

Our data support a testable framework in which MSC-Exos are linked to coordinated immunometabolic remodeling and a bias toward effector-like maturation in *ex vivo*-expanded human NK cells, while highlighting the need for targeted mechanistic studies to define the underlying causal pathways and the mode of exosomal cargo action.

Methods

Isolation, purification, and characterization of MSC-Exos

Human umbilical cord-derived mesenchymal stem cells (hUC-MSCs; Cyagen Biosciences, China) were cultured in Xeno-Free Human Mesenchymal Stem Cell Medium (Applied Cell, China) at 37 °C in a humidified incubator with 5% CO_2. According to the manufacturer, this xeno-free medium is produced under serum-free conditions and contains no exogenous animal-derived protein components; therefore, conditioned medium for MSC-Exos isolation was collected under serum-free culture conditions. Conditioned medium was collected and stored at −80 °C until further processing.

MSC-Exos were isolated from MSC culture supernatants using SEC, following previously published SEC optimization protocols [38]. A Sepharose CL-6B column (10 mL bed volume) was pre-equilibrated with phosphate-buffered saline (PBS) and stored overnight at 4 °C to stabilize the resin. Supernatants were sequentially centrifuged at 300 × g for 5 min and 3,000 × g for 5 min to remove cells and debris, followed by filtration through a 0.22 µm membrane. The filtrate was concentrated to 1 mL using a 50 kDa molecular weight cut-off ultrafiltration device (3,000 × g for 30 min) and loaded onto the CL-6B column. After loading, the column was eluted with PBS in 500 µL increments. The first 2 mL of flow-through was discarded, and subsequent fractions were collected in 2 mL volumes. Exosome-rich SEC fractions were pooled based on high particle counts and minimal soluble-protein carryover, operationalized by a higher particle-to-protein

ratio as a relative purity metric, consistent with published SEC optimization protocols.

Purified vesicles were characterized using orthogonal approaches. Morphology was assessed by TEM, particle size distribution was measured by NTA, and exosomal identity was confirmed by immunoblotting for positive markers (CD63, TSG101, and Syntenin) and the absence of the negative marker Calnexin.

Ex vivo expansion of peripheral blood–derived NK cells and intervention with MSC-Exos

All human PBMCs were obtained under approval from the Drug Clinical Trial Ethics Committee of Liaocheng Second People's Hospital and BGI Research. PBMCs from healthy donors were expanded *ex vivo* for 16 days using a commercial NK cell expansion kit (Jiake Biotechnology, China) to generate NK cell products. Beginning on day 10 of culture, the MSC-Exos-treated group received MSC-Exos at final concentrations of 10–40 μg/mL in NK cell expansion medium. Fresh medium containing the corresponding exosome concentration was replenished every 3 days. The control group was maintained under identical conditions but received exosome-free medium during medium changes.

CCK-8 assay for evaluating the effect of MSC-Exos on NK cell viability

To assess the effect of MSC-Exos on NK cell viability, expanded NK cells were collected on day 10 and seeded into 96-well plates at 5×10^4 cells per well in a total volume of 100 μL, with at least three replicate wells per condition. Cells were then cultured either in NK cell expansion medium alone (CON) or in the same medium supplemented with MSC-Exos (EXO) (10–40 μg/mL). Cell viability was measured on days 11, 13, and 16, corresponding to 1, 3, and 6 days after intervention, respectively. Medium-only wells were included as blanks.

For the day 16 measurement, 50% of the medium was replaced on day 13 with fresh medium containing the corresponding treatment, with MSC-Exos added to the EXO group at the same final concentration. At each indicated time point, 10 μL of CCK-8 reagent was added to each well, followed by incubation at 37 °C for 2 h in the dark. The OD was measured at 450 nm using a microplate reader, and following blank subtraction, the absorbance values were used to calculate relative cell viability. Data are presented as mean ± standard deviation (SD) from at least three replicate wells. Relative cell viability was calculated using the following formula:

$$\text{Relative activity} = \frac{(\text{OD}_{\text{sample}} - \text{OD}_{\text{blank}})}{(\text{OD}_{\text{control}} - \text{OD}_{\text{blank}})}$$

Induction of senescence in HDFs

Primary HDFs (Jinyuan Biotechnology, China) were cultured in DMEM/Ham's F-12 (1:1) supplemented with 10% fetal bovine serum (FBS) and 4 mM L-glutamine at 37 °C in a humidified 5% CO_2 atmosphere. To induce senescence, confluent HDFs were treated with DOX for 48 h. After treatment, medium was replaced with fresh complete medium, and cells were incubated for an additional 4–6 days to allow full development of senescence-associated phenotypes. Senescence was quantified using the Senescence β-Galactosidase Staining Kit (MedChemExpress, USA) according to the manufacturer's instructions. Cultures were considered significantly senescent when more than 50% of cells were positive for β-galactosidase staining.

qRT-PCR

Total RNA was extracted using the FastPure Cell/Tissue Total RNA Isolation Kit V2 (Vazyme, China). RNA concentration and purity were assessed with a NanoDrop 2000 spectrophotometer (Thermo Fisher Scientific, USA). Complementary DNA (cDNA) was synthesized from 1 μg of total RNA using the HiScript II Q RT SuperMix for qPCR kit (Vazyme, China). qRT-PCR was performed using SYBR Green master mix (Yeasen, China) and primers (**Supplementary Table S3**) on a QuantStudio 5 Real-Time PCR System (Applied Biosystems, USA). Relative gene expression was calculated using the $2^{(-\Delta\Delta Ct)}$ method and normalized to *ACTB*.

***In vitro* cytotoxicity assay of NK cells**

NK cell cytotoxicity was assessed using the CytoTox 96® Non-Radioactive Cytotoxicity Assay (Promega, USA), which quantifies lactate dehydrogenase (LDH) release following target-cell lysis. For antitumor activity, NK cells from EXO and CON groups were co-cultured with K562 target cells at effector-to-target (E:T) ratios of 5:1 and 20:1 for 5 h. To assess senescent-cell clearance, NK cells were co-cultured with DOX-induced senescent HDFs at identical E:T ratios for 20 h. Prior to co-culture, HDFs were trypsinized and counted to ensure accurate E:T setup.

After incubation, supernatants were collected and the OD was measured at 490 nm with a reference wavelength of 680 nm using a microplate reader. Cytotoxicity was calculated according to the manufacturer's instructions. All experiments were performed in triplicate, and data are presented as mean ± SD. NK-cell-mediated cytotoxicity was calculated using the following formula:

$$\text{NK cytotoxicity (\%)} = \frac{(\text{OD}_{\text{experimental}} - \text{OD}_{\text{spontaneous target}} - \text{OD}_{\text{spontaneous effector}})}{(\text{OD}_{\text{maximum release}} - \text{OD}_{\text{spontaneous target}})} \times 100\%.$$

Flow cytometry

For cell surface staining, single-cell suspensions were incubated with fluorochrome-conjugated antibodies against CD56, CD3, CD16, LAG3, NKG2D, NKG2C, and CD107a at 4 °C for 20 min in the dark, followed by washing with FACS buffer. For intracellular staining, cells were fixed and permeabilized using the Cyto-Fast™ Fix/Perm Buffer Set (Biolegend, USA) and then incubated with antibodies against granzyme B at room temperature for 20 min in the dark. Samples were acquired on a BD FACSAria III flow cytometer and analyzed using FlowJo (v10.0).

Mitochondrial membrane potential was assessed using the JC-1 Mitochondrial Membrane Potential Assay Kit (Yeasen, China). Mitochondrial mass and activity were evaluated using MitoTracker® Green FM and MitoTracker® Red CMXRos (Yeasen, China), respectively, followed by flow cytometric detection.

Cytometric bead array (CBA) measurement of IFN-γ

MSC-Exos-treated and control NK cells were co-cultured with K562 target cells at an E:T ratio of 5:1 for 12 h. Cell suspensions were centrifuged at 500 × g for 5 min, and supernatants were collected for cytokine quantification. IFN-γ concentrations were measured using the CBA Human Soluble Protein Master Buffer Kit (BD Biosciences, USA) according to the manufacturer's instructions. Capture beads were incubated with supernatants for 1 h at room temperature in the dark, followed by addition of PE-conjugated detection reagent for 2 h to form bead–cytokine–detector complexes. Beads were washed, resuspended, acquired on a BD FACSAria III, and analyzed using FCAP Array software. Results are reported as pg/mL based on standard curves.

Single-cell RNA-seq data processing and analysis

Raw sequencing reads were filtered, demultiplexed, and aligned to the hg38 human reference genome using a custom pipeline [39]. Only reads aligned to annotated gene exons were counted. Potential doublets were identified and removed using DoubletFinder (v2.0.3), and ambient RNA contamination was corrected using SoupX (v1.4.8) under default parameters.

Cells were retained based on the following quality thresholds: 500–20,000 UMIs and 500-6,000 detected genes, and $<10\%$ mitochondrial gene content. Downstream analyses were performed using Scanpy (v1.9.3) in Python 3.7. After library-size normalization and log transformation, the 2,000 most variable genes were selected. UMI counts and mitochondrial percentages were regressed out, and the expression matrix was scaled. Dimensionality reduction was performed by principal component analysis (PCA), followed by batch

correction with Harmony (v0.0.10) with theta = 5 and max.iter.harmony = 50; all other parameters were kept at their default values. The top 30 Harmony-corrected principal components were used to construct a k-nearest neighbor (kNN) graph (k = 15). Clustering was performed using the Leiden algorithm (Scanpy implementation) with resolution = 0.1, and clusters were annotated based on canonical marker genes.

Major immune lineages were annotated using canonical marker sets (T cell markers: *CD3D, CD3E, CD4, IL7R, CD8A, CD8B*; B cell markers: *CD79A, MS4A1*; NK cell markers: *NCAM1, KLRD1, FCGR3A, GNLY, NKG7*). Non-NK-lineage cells were excluded prior to generating the NK-cell-only subset used for integration, clustering, differential expression, and trajectory inference. Additionally, clusters lacking canonical NK cell markers and exhibiting low marker specificity were considered transcriptionally ambiguous and removed to reduce the influence of low-quality cells, potential doublets, or uninformative populations. After subsetting to NK cells, batch correction was re-applied with Harmony (using theta = 2 and max.iter.harmony = 50) and the kNN graph was recomputed, followed by de novo UMAP embedding and Leiden clustering (with resolution = 0.4) on the NK-cell-only dataset. NK cell subclusters were then annotated and used for downstream comparisons between the MSC-Exos-treated and untreated conditions. Differential expression analysis between the CON and EXO groups was performed using Scanpy. Genes with $|\log_2(\text{fold change})| > 0.25$ and an adjusted p value < 0.05 (Benjamini-Hochberg corrected) were identified as differentially expressed genes (DEGs).

Pseudotime trajectory analysis

Pseudotime trajectory analysis was performed on NK cells extracted from the integrated scRNA-seq dataset to infer differentiation dynamics. For Monocle 2 (v2.26.0), we selected ordering genes as cell-type differential genes across the four NK cell subclusters (qval < 0.01) and reduced dimensionality using DDRTree to reconstruct branched trajectories; the root state was determined automatically by Monocle 2. In parallel, we used Monocle 3 (v1.0.0) with UMAP for dimensionality reduction to validate the global differentiation manifold. The pseudotime root node was specified based on cell-type annotation, using cells from the CD56^{bright} subset as the starting population. Together, these complementary analyses mapped a continuum from early regulatory-like states toward mature cytotoxic effector phenotypes based on dynamic, lineage-associated transcriptional programs.

Metabolic profiling

Metabolic pathway activity scores were computed using scMetabolic (v0.2.1) with the built-in KEGG

metabolism gene sets under default settings, enabling single-cell level comparison of energy metabolism and biosynthetic programs between treatment groups.

Gene ontology and gene set enrichment analysis

GO enrichment analyses were performed in R using clusterProfiler (SCR_016884). The enrichGO function was executed with Benjamini–Hochberg correction and a false discovery rate (FDR) threshold of $q < 0.01$. Curated gene sets (c2.cp.v2025.1.Hs.symbols.gmt) were obtained from the Molecular Signatures Database (MSigDB). For single-sample and preranked enrichment analyses, GSEApy (SCR_025803, v1.1.8) was used in Python, with parameters set to min_size = 5, max_size = 1000, and 1000 permutations. Gene sets with FDR $q < 0.25$ were considered significantly enriched.

Exosome proteomic analysis

Purified MSC-Exos were resuspended in lysis buffer containing 8 M urea and 1× protease inhibitor cocktail. Samples were lysed by ultrasonication and centrifuged at 25,000 × g for 15 min at 4 °C to collect supernatants for protein quantification. Proteins were reduced with 10 mM dithiothreitol (DTT) at 37 °C for 30 min and alkylated with 55 mM iodoacetamide (IAM) in the dark for 45 min. Trypsin was added at a 1:50 (w/w) enzyme-to-substrate ratio and digestion was performed at 37 °C for 8 h. Peptides were purified and desalted using a C18 column, collected by centrifugation (20,000 × g, 10 min, 4 °C), and reconstituted in mobile phase A (0.1% formic acid in ultrapure water) for liquid chromatography-tandem mass spectrometry (LC-MS/MS) analysis.

Raw mass spectrometry data were processed using Spectronaut 17 (Biognosys) and searched against the UniProt Homo sapiens database (20,360 Swiss-Prot entries) using the directDIA+ (Deep) workflow. This library-free DIA strategy was coupled with label-free quantification (LFQ) to enable high-coverage protein identification and accurate quantification without a prebuilt spectral library, generating a global proteomic landscape and differential abundance profiles for MSC-Exos.

Statistical analysis

Data are presented as mean ± SD, unless otherwise stated in the figure legends. Statistical analyses were performed using GraphPad Prism 8.3.1. For comparisons between two groups, a two-tailed Student's t-test was used for normally distributed data, with paired or unpaired tests applied according to the experimental design. For non-normally distributed data, group comparisons were performed using a two-sided Mann-Whitney U test. For single-cell transcriptomic analyses, differential expression between groups was assessed

using a two-sided Wilcoxon rank-sum test. Statistical significance was defined as *P < 0.05, **P < 0.01, ***P < 0.001, and ****P < 0.0001. The definition of n (biological replicates vs technical replicates) and the specific test used for each analysis are provided in the corresponding figure legends.

Availability of source code and requirements

Project name: NK-MSC-exos-scRNAseq-analysis

Project homepage: https://github.com/fuyunyun-95/NK-MSC-exos-scRNAseq-analysis.git

Operating system: Linux

Programming language: Python and R

Package management: Conda

Hardware requirements: Tested on a laptop with 8-core CPU, 64 GB RAM and 256 GB SSD

License: MIT License

Data Availability

The scRNA-seq data generated in this study and supporting its findings have been deposited in the China National GeneBank Sequence Archive (CNSA) [40] under accession number CNP0008912 and in the Genome Sequence Archive (GSA) under accession number PRJCA057478. The mass spectrometry proteomics data have been deposited to the ProteomeXchange Consortium via the PRIDE partner repository with the accession number PXD073707.

Abbreviations

ADCC: antibody-dependent cellular cytotoxicity; ADCP: antibody-dependent cellular phagocytosis; CBA: cytometric bead array; CCK-8: Cell Counting Kit-8; cDNA: complementary DNA; CNSA: China National GeneBank Sequence Archive; CON: the control group; DE: differential expression; DEGs: differentially expressed genes; DOX: doxorubicin; DTT: dithiothreitol; EXO: the MSC-Exos-treated group; FBS: fetal bovine serum; GSA: Genome Sequence Archive; GSEA: gene set enrichment analysis; GvHD: graft-versus-host disease; HDFs: human dermal fibroblasts; IAM: iodoacetamide; LC-MS/MS: liquid chromatography-tandem mass spectrometry; LDH: lactate dehydrogenase; LFQ: label-free quantification; MFI: mean

fluorescence intensity; MISEV: Minimal Information for Studies of Extracellular Vesicles; MSCs: mesenchymal stem cells; MSC-Exos: mesenchymal stem cell-derived exosomes; NK: natural killer; NTA: nanoparticle tracking analysis; non-MHC: non-major histocompatibility complex; OD: optical density; PBMCs: peripheral blood mononuclear cells; PBS: phosphate-buffered saline; PCA: principal component analysis; qRT-PCR: quantitative real-time polymerase chain reaction; SASP: senescence-associated secretory phenotype; SA-β-Gal: senescence-associated β-galactosidase; SD: standard deviation; scRNA-seq: single-cell RNA sequencing; SEC: size-exclusion chromatography; TEM: transmission electron microscopy.

Declarations

Not applicable.

Consent for publication

Not applicable.

Competing interests

The authors declare that they have no competing interests.

Funding

This research received no specific grant from any funding agency in the public, commercial, or not-for-profit sectors.

Authors' contributions

YY.F.: investigation, methodology, formal analysis, validation, visualization, writing–original draft; Y.L.: methodology, formal analysis, visualization, writing–review & editing; M.X.: formal analysis, visualization; G.L.: formal analysis, visualization; J.S.: formal analysis, visualization; F.B.: formal analysis, visualization; W.X.: validation; J.Z.: validation; J.L.: validation; Q.G.: methodology; Y.H.: resources; F.X.: resources; S.L.: resources, project administration; L.L.: resources; Y.F.: supervision, writing–review & editing; X.D.: conceptualization, project administration, supervision, resources, writing–review & editing.

Acknowledgements

We would like to thank DCS Cloud (https://cloud.stomics.tech/) for providing the computational resources and software support necessary for this study.

References

1. Deng X and Terunuma H. Adoptive NK cell therapy: a potential revolutionary approach in longevity therapeutics. Immun Ageing. 2024;21 1:43. doi:10.1186/s12979-024-00451-2.
2. Sagiv A, Burton DG, Moshayev Z, Vadai E, Wensveen F, Ben-Dor S, et al. NKG2D ligands mediate immunosurveillance of senescent cells. Aging (Albany NY). 2016;8 2:328-44. doi:10.18632/aging.100897.
3. Brighton PJ, Maruyama Y, Fishwick K, Vrljicak P, Tewary S, Fujihara R, et al. Clearance of senescent decidual cells by uterine natural killer cells in cycling human endometrium. Elife. 2017;6 doi:10.7554/eLife.31274.
4. Antonangeli F, Zingoni A, Soriani A and Santoni A. Senescent cells: Living or dying is a matter of NK cells. J Leukoc Biol. 2019;105 6:1275-83. doi:10.1002/JLB.MR0718-299R.
5. Bryceson YT, March ME, Ljunggren H-G and Long EO. Synergy among receptors on resting NK cells for the activation of natural cytotoxicity and cytokine secretion. Blood. 2006;107 1:159-66. doi:10.1182/blood-2005-04-1351.
6. Myers JA and Miller JS. Exploring the NK cell platform for cancer immunotherapy. Nature Reviews Clinical Oncology. 2020;18 2:85-100. doi:10.1038/s41571-020-0426-7.
7. Zhao W, Zhang H, Liu R and Cui R. Advances in Immunomodulatory Mechanisms of Mesenchymal Stem Cells-Derived Exosome on Immune Cells in Scar Formation. International Journal of Nanomedicine. 2023;Volume 18:3643-62. doi:10.2147/ijn.S412717.
8. Kalluri R and LeBleu VS. The biology, function, and biomedical applications of exosomes. Science. 2020;367 6478 doi:10.1126/science.aau6977.
9. Tse WT, Pendleton JD, Beyer WM, Egalka MC and Guinan EC. Suppression of allogeneic T-cell proliferation by human marrow stromal cells: implications in transplantation. Transplantation. 2003;75 3:389-97. doi:10.1097/01.Tp.0000045055.63901.A9.

10. Bartholomew A, Sturgeon C, Siatskas M, Ferrer K, McIntosh K, Patil S, et al. Mesenchymal stem cells suppress lymphocyte proliferation in vitro and prolong skin graft survival in vivo. Exp Hematol. 2002;30 1:42-8. doi:10.1016/s0301-472x(01)00769-x.

11. Glennie S, Soeiro Is, Dyson PJ, Lam EWF and Dazzi F. Bone marrow mesenchymal stem cells induce division arrest anergy of activated T cells. Blood. 2005;105 7:2821-7. doi:10.1182/blood-2004-09-3696.

12. Zhou Y, Day A, Haykal S, Keating A and Waddell TK. Mesenchymal stromal cells augment CD4+ and CD8+ T-cell proliferation through a CCL2 pathway. Cytotherapy. 2013;15 10:1195-207. doi:10.1016/j.jcyt.2013.05.009.

13. Misaghian A, Ghadiri AA, Asadirad A, Amirzadeh S and Amari A. The Effect of Exosomes Isolated from Poly (I:C) Treated Human Wharton's Jelly Mesenchymal Stem Cells on CD4+CD25+Foxp3+ Regulatory T Cells. Iranian Journal of Allergy, Asthma and Immunology. 2024; doi:10.18502/ijaai.v23i3.15638.

14. Arabpour M, Saghazadeh A and Rezaei N. Anti-inflammatory and M2 macrophage polarization-promoting effect of mesenchymal stem cell-derived exosomes. Int Immunopharmacol. 2021;97 doi:10.1016/j.intimp.2021.107823.

15. Yang L, Wang X, Wei X, Yu P, Liu Y, Wang S, et al. Oral Delivery of R-spondin1-Loaded Small Extracellular Vesicles Activates WNT Signalling Pathway to Accelerate Intestinal Injury Repair and Reverse Ageing. J Extracell Vesicles. 2026;15 1:e70226. doi:10.1002/jev2.70226.

16. Yang L, Wang S, Qiao Z, Liu Y, Wang X, Liu L, et al. Dual-ligand engineered exosome regulates WNT signaling activation to promote liver repair and regeneration. Nat Commun. 2025;16 1:9019. doi:10.1038/s41467-025-64069-8.

17. Ding C, Zheng Y, Li D, Zhu M and Zhu Y. Up-Regulation of miR-1925 by Bone Marrow Mesenchymal Stem Cell (BMSC) Inhibits the Growth of Liver Cancer by Promoting the Anti-Tumor Activity of Natural Killer (NK) Cells. Journal of Biomaterials and Tissue Engineering. 2022;12 3:630-3.

18. Cui R, Rekasi H, Hepner-Schefczyk M, Fessmann K, Petri RM, Bruderek K, et al. Human mesenchymal stromal/stem cells acquire immunostimulatory capacity upon cross-talk with natural killer cells and might improve the NK cell function of immunocompromised patients. Stem Cell Res Ther. 2016;7 1:88. doi:10.1186/s13287-016-0353-9.

19. Rebuffet L, Melsen JE, Escalière B, Basurto-Lozada D, Bhandoola A, Björkström NK, et al. High-dimensional single-cell analysis of human natural killer cell heterogeneity. Nat Immunol. 2024;25 8:1474-88. doi:10.1038/s41590-024-01883-0.

20. Powell JE. Charting immune variation through genetics and single-cell genomics. Gigascience. 2026;15 doi:10.1093/gigascience/giaf161.

21. Luo J, Guo M, Huang M, Liu Y, Qian Y, Liu Q, et al. Neoleukin-2/15-armored CAR-NK cells sustain superior therapeutic efficacy in solid tumors via c-Myc/NRF1 activation. Signal Transduct Target Ther. 2025;10 1:78. doi:10.1038/s41392-025-02158-2.

22. Poznanski SM, Singh K, Ritchie TM, Aguiar JA, Fan IY, Portillo AL, et al. Metabolic flexibility determines human NK cell functional fate in the tumor microenvironment. Cell Metabolism. 2021;33 6:1205-20.e5. doi:10.1016/j.cmet.2021.03.023.

23. Zhang H, Xiao X, Wang L, Shi X, Fu N, Wang S, et al. Human adipose and umbilical cord mesenchymal stem cell-derived extracellular vesicles mitigate photoaging via TIMP1/Notch1. Signal Transduct Target Ther. 2024;9 1:294. doi:10.1038/s41392-024-01993-z.

24. Nimmerjahn F and Ravetch JV. Fcγ receptors as regulators of immune responses. Nature Reviews Immunology. 2008;8 1:34-47. doi:10.1038/nri2206.

25. Capuano C, Pighi C, Battella S, De Federicis D, Galandrini R and Palmieri G. Harnessing CD16-Mediated NK Cell Functions to Enhance Therapeutic Efficacy of Tumor-Targeting mAbs. Cancers. 2021;13 10 doi:10.3390/cancers13102500.

26. Galvez-Cancino F, Simpson AP, Costoya C, Matos I, Qian D, Peggs KS, et al. Fcγ receptors and immunomodulatory antibodies in cancer. Nat Rev Cancer. 2024;24 1:51-71. doi:10.1038/s41568-023-00637-8.

27. Szmania S, Lapteva N, Garg T, Greenway A, Lingo J, Nair B, et al. Ex Vivo–expanded Natural Killer Cells Demonstrate Robust Proliferation In Vivo in High-risk Relapsed Multiple Myeloma Patients. Journal of Immunotherapy. 2015;38 1:24-36. doi:10.1097/cji.0000000000000059.

28. Granzin M, Soltenborn S, Müller S, Kollet J, Berg M, Cerwenka A, et al. Fully automated expansion and activation of clinical-grade natural killer cells for adoptive immunotherapy. Cytotherapy. 2015;17 5:621-32. doi:10.1016/j.jcyt.2015.03.611.

29. Liu X, Wei Q, Lu L, Cui S, Ma K, Zhang W, et al. Immunomodulatory potential of mesenchymal stem cell-derived extracellular vesicles: Targeting immune cells. Front Immunol. 2023;14 doi:10.3389/fimmu.2023.1094685.

30. Fan Y, Herr F, Vernochet A, Mennesson B, Oberlin E and Durrbach A. Human Fetal Liver Mesenchymal Stem Cell-Derived Exosomes Impair Natural Killer Cell Function. Stem Cells Dev. 2019;28 1:44-55. doi:10.1089/scd.2018.0015.

31. Almutairi A, Alshehri NA, Al Subayyil A, Bahattab E, Alshabibi M, Abomaray F, et al. Human decidua basalis mesenchymal stem/stromal cells enhance anticancer properties of human natural killer cells, in vitro. Front Cell Dev Biol. 2024;12:1435484. doi:10.3389/fcell.2024.1435484.

32. Vivier E, Tomasello E, Baratin M, Walzer T and Ugolini S. Functions of natural killer cells. Nat Immunol. 2008;9 5:503-10. doi:10.1038/ni1582.

33. Long EO, Kim HS, Liu D, Peterson ME and Rajagopalan S. Controlling natural killer cell responses: integration of signals for activation and inhibition. Annu Rev Immunol. 2013;31:227-58. doi:10.1146/annurev-immunol-020711-075005.

34. Schoenborn JR and Wilson CB. Regulation of interferon-gamma during innate and adaptive immune responses. Adv Immunol. 2007;96:41-101. doi:10.1016/s0065-2776(07)96002-2.

35. Wang T, Jian Z, Baskys A, Yang J, Li J, Guo H, et al. MSC-derived exosomes protect against oxidative stress-induced skin injury via adaptive regulation of the NRF2 defense system. Biomaterials. 2020;257 doi:10.1016/j.biomaterials.2020.120264.

36. Dinkova-Kostova AT and Abramov AY. The emerging role of Nrf2 in mitochondrial function. Free Radical Biology and Medicine. 2015;88:179-88. doi:10.1016/j.freeradbiomed.2015.04.036.

37. Bi Y, Qiao X, Cai Z, Zhao H, Ye R, Liu Q, et al. Exosomal miR-302b rejuvenates aging mice by reversing the proliferative arrest of senescent cells. Cell Metab. 2025;37 2:527-41.e6. doi:10.1016/j.cmet.2024.11.013.

38. Guo J, Wu C, Lin X, Zhou J, Zhang J, Zheng W, et al. Establishment of a simplified dichotomic size-exclusion chromatography for isolating extracellular vesicles toward clinical applications. J Extracell Vesicles. 2021;10 11:e12145. doi:10.1002/jev2.12145.

39. DNBelab C Series™ HT Single-Cell Analysis Software. https://github.com/MGI-tech-bioinformatics/DNBelab_C_Series_HT_scRNA-analysis-software. Accessed 10 June 2025.

40. Wang W, Tan C, Li L, Li X, Zhang L, Li X, et al. The China National GeneBank Sequence Archive (CNSA) 2024 update. Horticulture Research. 2025;12 5 doi:10.1093/hr/uhaf036.

Figure legend

Figure 1. MSC-Exos improve NK cell viability during *ex vivo* expansion while preserving an NK-cell-enriched phenotype.

(A) Experimental design and workflow. PBMCs were cultured for *ex vivo* NK cell expansion from day 0 to day 10. From day 10 to day 16, the EXO group received MSC-Exos every 3 days, whereas the CON group was maintained without exosome supplementation. Endpoint analyses included flow cytometry, CCK-8-based viability assessment, *in vitro* cytotoxicity assays, and scRNA-seq. **(B)** Representative TEM image showing the morphology of MSC-Exos (scale bar, 100 nm). **(C)** NTA showing the size distribution profile of MSC-Exos. **(D)** Immunoblotting analysis of MSC-Exos showing enrichment of exosomal markers (CD63, Syntenin, and TSG101) and absence of the endoplasmic reticulum marker Calnexin. **(E)** Quantification of NK cell viability in donor NK001 by CCK-8 assay (OD450) following treatment with MSC-Exos at 10–40 μg/mL. **(F)** Quantification of NK cell viability in donor NK001 by CCK-8 assay (OD450) following treatment with 20 μg/mL MSC-Exos during the late expansion phase, with measurements collected on days 11, 13, and 16. **(G)** Flow cytometry analysis showing comparable frequencies of $CD56^{+}CD3^{-}$ NK cells between the CON and EXO groups. Statistics: For panels E–F, data are presented as mean ± SD from n = 4 technical replicates, and significance was evaluated using an unpaired two-tailed Student's t-test. For panel G, n = 5 biological replicates (donors) were analyzed using a paired two-tailed Student's t-test (paired samples are connected by lines). ns, not significant; **P < 0.01.

Figure 2. MSC-Exos are associated with enhanced NK cell cytotoxicity, activating receptor expression, degranulation, and mitochondrial fitness-related readouts.

(A) Percentage lysis of K562 tumor cells by NK cells from different donors in the CON and EXO groups. The effector-to-target (E:T) ratio was 20:1. **(B)** Percentage lysis of DOX-induced senescent HDFs by NK cells from different donors in the CON and EXO groups. The E:T ratio was 20:1. **(C–E)** Flow cytometric analysis of the percentages of NK cells expressing the activating receptors CD16 (C), NKG2D (D), and NKG2C (E) from different donors in the CON and EXO groups. **(F)** Mean fluorescence intensity (MFI) of the inhibitory receptor LAG3 on NK cells from different donors in the CON and EXO groups. **(G)** Degranulation of NK cells, measured as the percentage of $CD107a^{+}$ cells after stimulation with K562 target cells. **(H)** Granzyme B expression in NK cells, measured as the percentage of GrB^{+} cells after stimulation with K562 target cells. **(I)** Mitochondrial staining-based readout in NK cells assessed by MitoTracker

Red/Green under basal conditions and after stimulation with K562 cells. **(J)** Mitochondrial membrane potential (ΔΨm) in NK cells, expressed as the JC-1 red/green fluorescence intensity ratio under basal conditions and after stimulation with K562 cells. **(K)** Relative mRNA expression of *NFE2L2* (NRF2), a key antioxidant regulator, in NK cells from different donors, as determined by qRT-PCR. Statistics: For panels A–K, data are presented as mean ± SD from n = 3 technical replicates, and significance was evaluated using an unpaired two-tailed Student's t-test. ns, not significant; $^{*}P < 0.05$, $^{**}P < 0.01$, $^{***}P < 0.001$.

Figure 3. Transcriptomic profiling reveals immunometabolic alterations associated with MSC-Exos treatment in NK cells.

(A) Experimental workflow for scRNA-seq of NK cells cultured with or without MSC-Exos. PBMCs from four independent healthy donors (NK001–NK004) were expanded for 16 days, with MSC-Exos added every 3 days during the final culture phase (days 10–16). Single-cell libraries were generated from each donor for group-level differential expression and subcluster analyses. **(B)** Heatmap showing the mean expression of cytotoxicity-associated genes (*FCGR3A, GZMB, NKG7, GNLY, IFNG, TNF*) across the CON and EXO groups. Gene expression is scaled by z-score. **(C)** Violin plots showing cytotoxic granule signature scores. **(D)** Violin plots showing signature scores for cellular senescence, SASP, and oxidative stress responses. **(E)** Volcano plot showing DEGs between EXO and CON groups, with selected genes of interest highlighted. **(F)** GO enrichment analysis of genes upregulated in the EXO group, highlighting biological processes related to cytoplasmic translation, immune activation, and cytokine responses. **(G)** GSEA showing enrichment of Fcγ receptor-dependent phagocytosis, Rho GTPase signaling, and glycolysis pathways in the EXO group (FDR $q < 0.25$). Statistics: For panels C–D, significance was assessed using a two-sided Mann-Whitney U test, $^{***}P < 0.001$. For panel E, differential expression was evaluated using a two-sided Wilcoxon rank-sum test.

Figure 4. Single-cell analyses suggest that MSC-Exos bias NK-cell subsets toward effector-cytotoxic states.

(A) UMAP plot showing four major NK cell subsets: Trans_NK, $CD56^{dim}$_prolif, $CD56^{dim}$_eff, and $CD56^{bright}$. **(B)** UMAP plots showing donor distribution (left) and treatment grouping (right), indicating comparable representation of subclusters across four donors and treatment conditions (CON vs EXO). **(C)**

Dot plot showing representative marker genes for inflammatory, proliferative, effector, and metabolic fitness signatures across NK cell subclusters, with dot size indicating fraction of cells per group and color indicating mean expression. **(D)** Box plot of NK cell subset proportions between CON and EXO groups. **(E)** Pseudotime analysis illustrating differentiation trajectories of NK cell subpopulations, with black lines representing predicted lineage paths. **(F)** Density distribution of pseudotime states across subclusters in CON and EXO groups. **(G)** Violin plots showing cytotoxicity expression scores of $CD56^{dim}$_prolif and $CD56^{dim}$_eff subsets between CON and EXO groups. **(H)** GO enrichment analysis of differentially expressed genes in $CD56^{dim}$_prolif and $CD56^{dim}$_eff subsets. Statistics: For panel D, significance was assessed using a paired two-tailed Student's t-test. For panel G, significance was assessed using a two-sided Mann-Whitney U test, ***P < 0.001.

Figure 5. Proteomic characterization of MSC-Exos and identification of FcγR-related signaling modules.

(A) Bar plot showing Jaccard similarity between gene sets represented by proteins identified in the MSC-Exos proteome and gene sets from pathways upregulated in NK cells by transcriptomic analysis. **(B)** Venn diagram showing the overlap between proteins identified in MSC-Exos and proteins annotated to the Reactome FcγR-dependent phagocytosis pathway. **(C)** Scatter plot showing the ranked normalized protein abundance in MSC-Exos, with selected components associated with the FcγR-dependent phagocytosis pathway (IGHG1, SRC, RAC1, PLCG2, BTK, and MAPK1) highlighted.

Figure5 Click here to access/download;Figure;Figure5.pdf

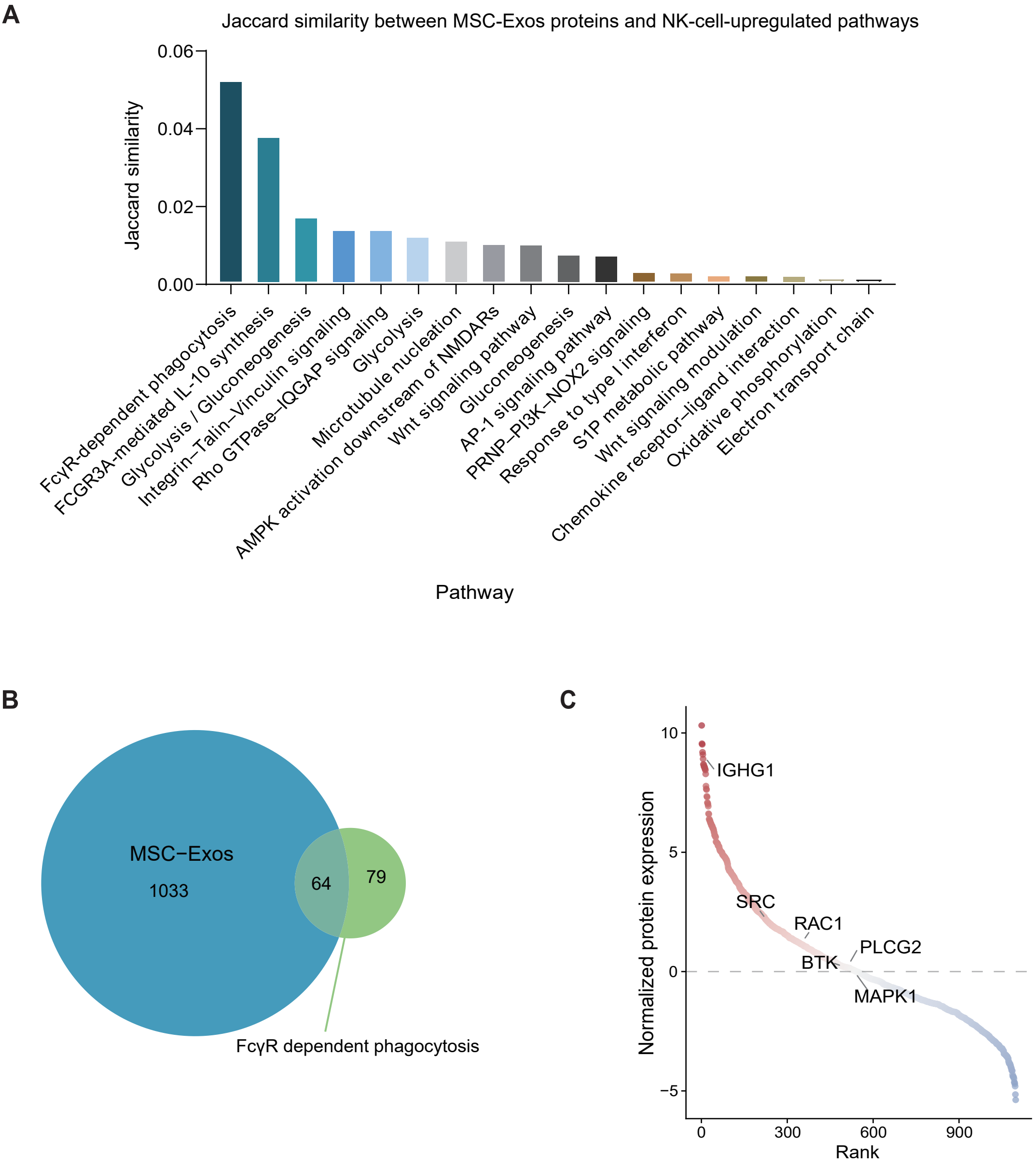

Figure4

Click here to access/download;Figure;Figure4.pdf

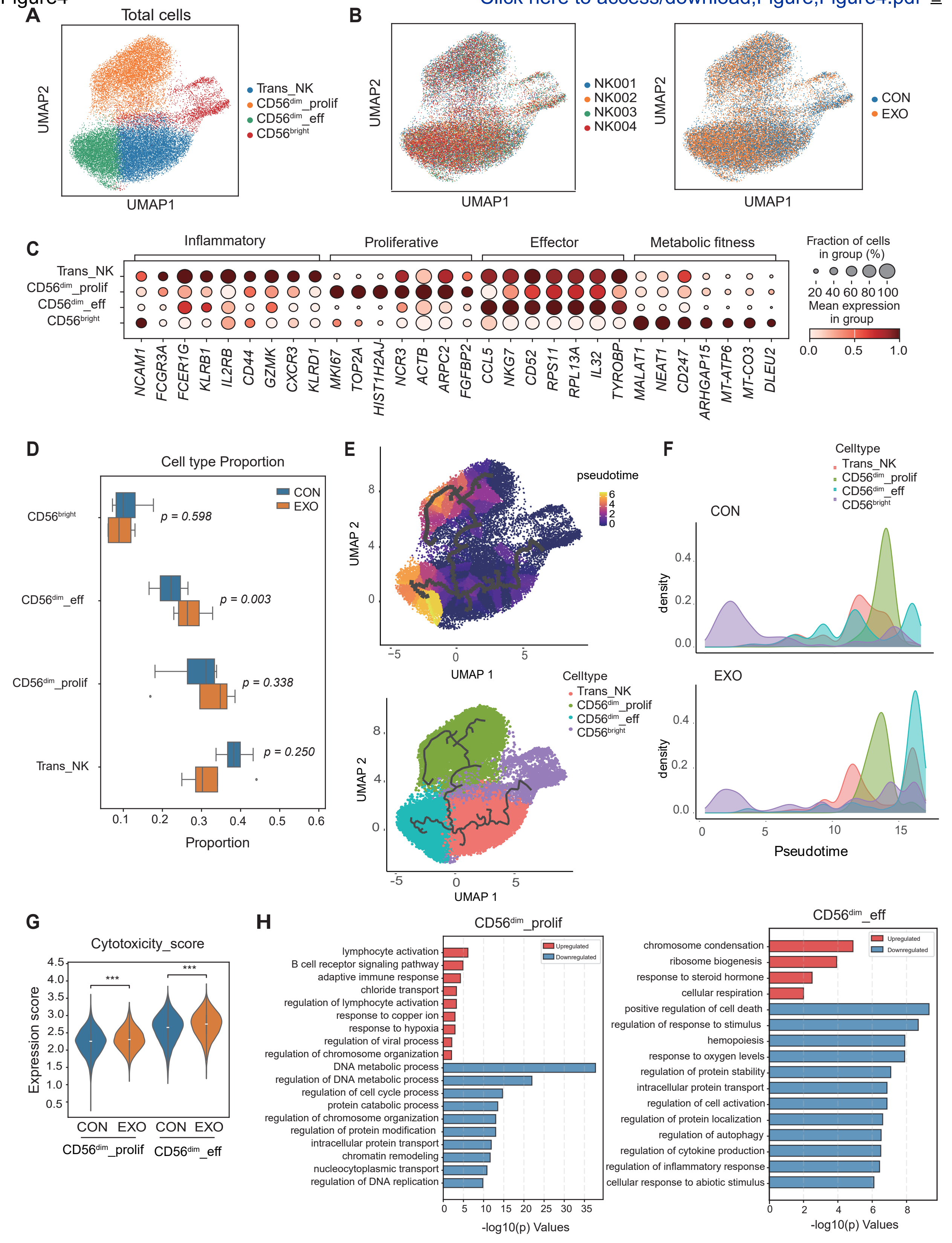

Figure3

Click here to access/download;Figure;Figure3.pdf

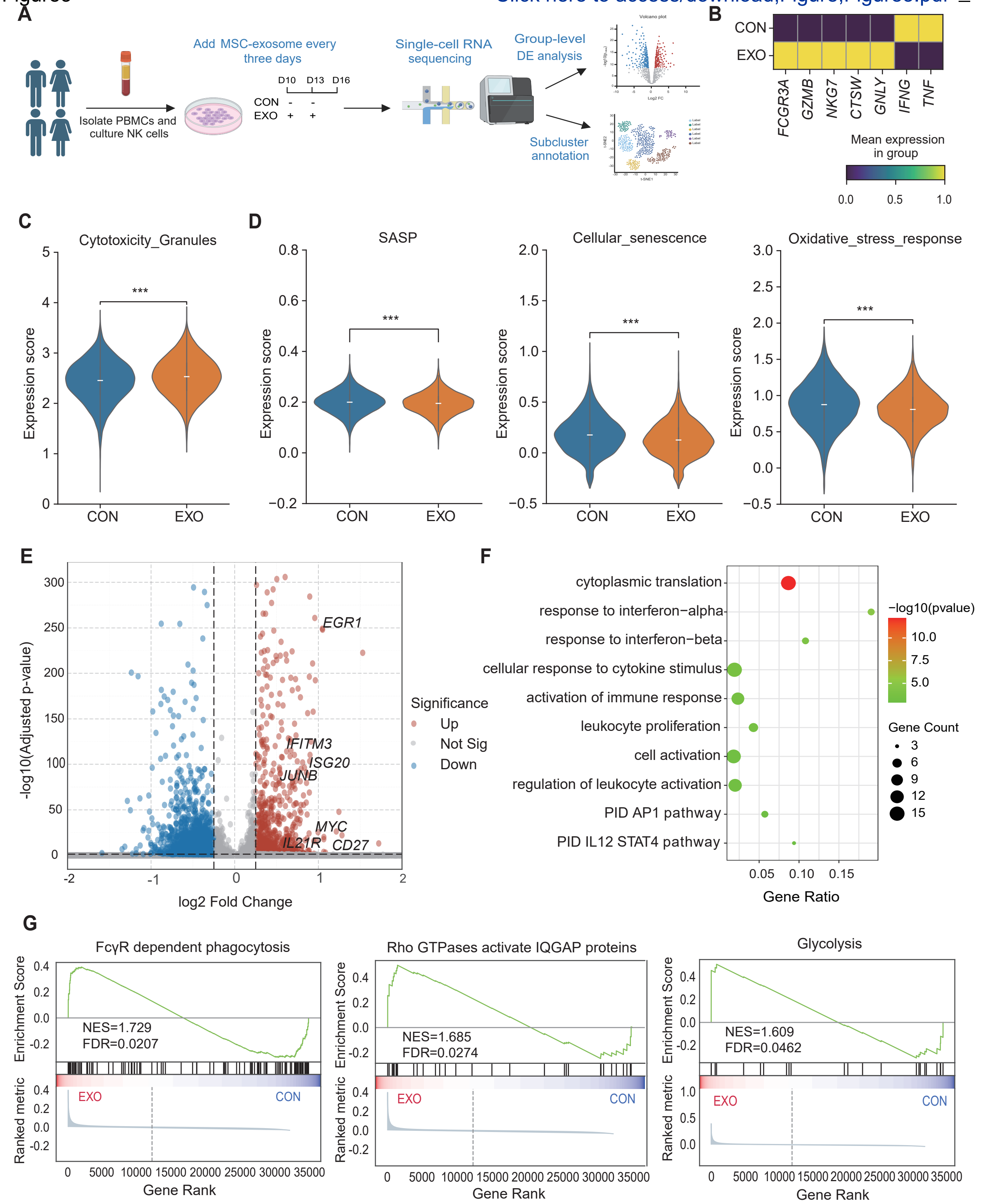

Figure2

Click here to access/download;Figure;Figure2.pdf

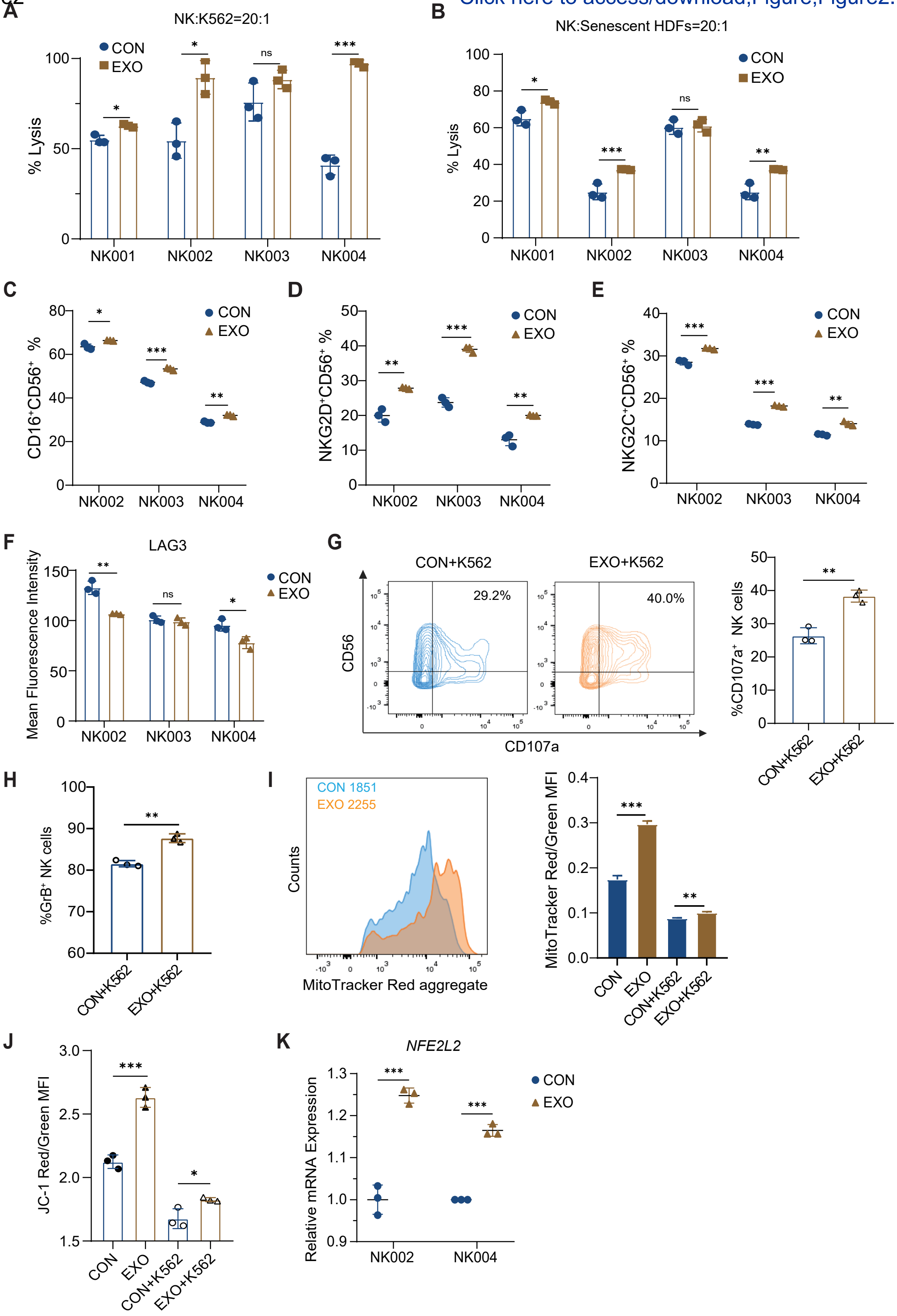

Figure1

Click here to access/download;Figure;Figure1.pdf

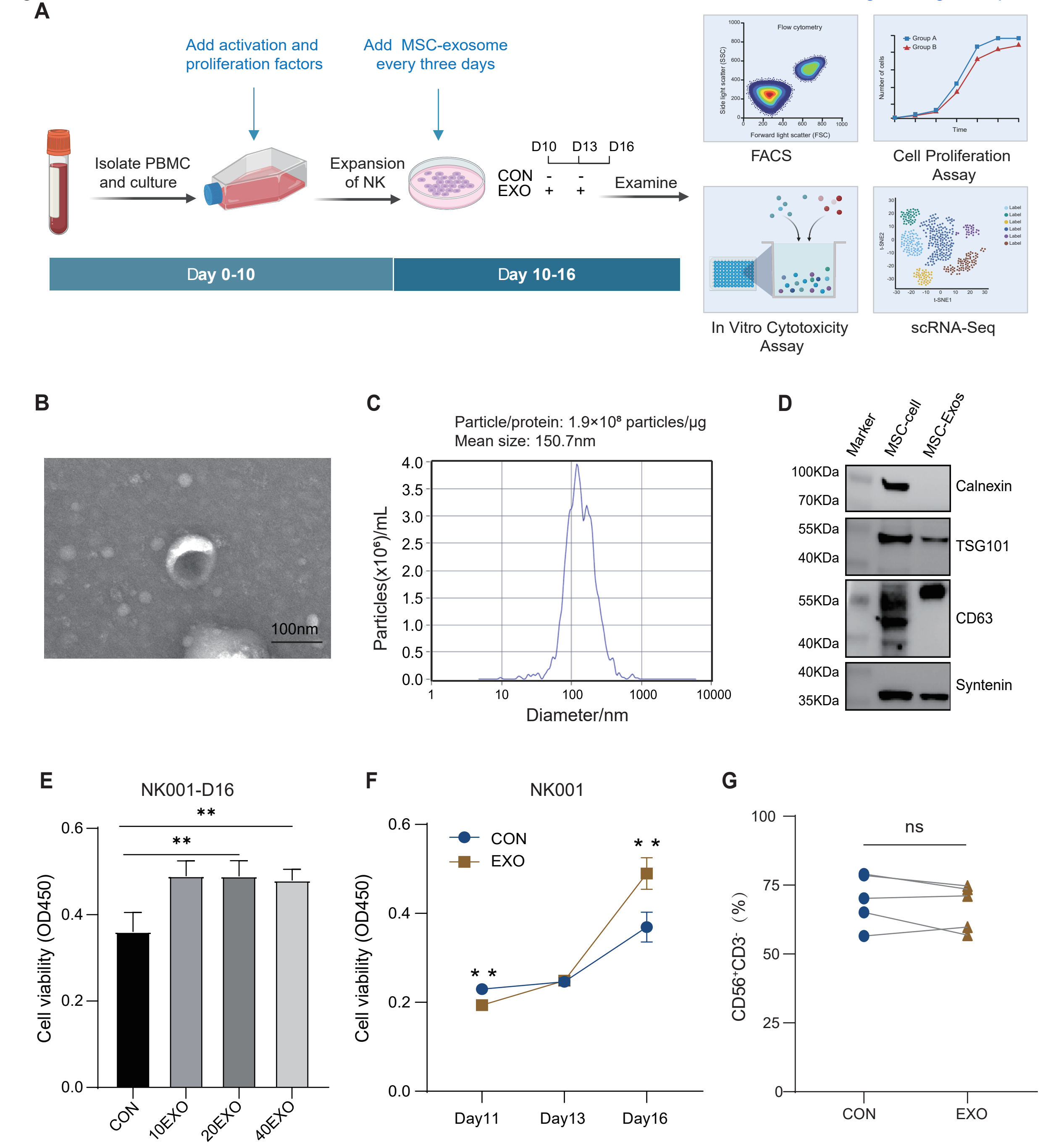

Supplementary Tables

Click here to access/download
Supplementary Material
Supplementary Tables_20260326.xlsx

Supplementary Figures

Click here to access/download
Supplementary Material
Supplementary Figures_260330.docx

Personal Cover

Click here to access/download;Personal Cover;renamed_bd766.docx

Dear Editors,

We are pleased to resubmit our revised manuscript entitled "**Integrative single-cell transcriptomics and MSC-exosome proteomics reveal a mechanistic basis for MSC-exosome–driven NK-cell expansion and effector reprogramming**" (GIGA-D-26-00032) for consideration for publication in *GigaScience*. We thank the editors and reviewers for their thoughtful and constructive comments, which have helped us substantially improve the clarity, rigor, and reproducibility of our work. All authors have reviewed and approved the revised manuscript.

In response to the reviewers' comments, we have made extensive revisions to the manuscript. Key improvements include:

1. Clarification of scRNA-seq analysis workflow: We have explicitly described the removal of non-NK lineages prior to NK-focused downstream analyses, and confirmed that all analyses (integration, clustering, trajectory inference) were re-performed *de novo* on the NK-only subset. Stepwise cell counts per donor and condition are now provided in a new supplementary table.

2. Enhanced methodological transparency: We now report key parameters for Harmony (theta, iterations), Leiden clustering (resolution), Monocle versions and settings, and scMetabolic gene-set sources. UMAP visualizations before and after Harmony correction (colored by donor, batch, and condition) are included in the Supplementary Information to improve transparency of batch correction.

3. Cautious interpretation of multi-omics integration: We have systematically revised language throughout the Abstract, Results, and Discussion to distinguish correlation from causality, replacing overstatements with phrases such as "is associated with," "is consistent with," and "supports a plausible hypothesis." We explicitly note that pathway overlap between exosome proteomics and NK transcriptomics is hypothesis-generating and does not demonstrate direct cargo delivery or functional necessity without targeted validation.

4. Improved exosome dosing and characterization: To enhance engineering-oriented reproducibility, we now report particle concentration (particles/mL), particle-to-cell dosing, protein-to-particle ratio, and the rationale for SEC fraction selection in both the main text and Supplementary Table 1.

5. Functional integration of single-cell findings: We have added cross-level synthesis statements linking scRNA-seq–defined NK subset shifts to cytotoxicity/degranulation assays and connecting metabolic pathway changes to mitochondrial readouts, strengthening the connection between transcriptomic observations and functional phenotypes.

6. Refined interpretation of FcγR-related enrichment: We now clarify that "FcγR-dependent phagocytosis" enrichment in NK cells more likely reflects cytoskeletal remodeling, immune-

synapse organization, and trogocytosis-like processes rather than canonical phagocytosis, with appropriate limiting language added to the Results and figure legends.

7. Discussion of IFN-γ reduction vs. enhanced cytotoxicity: We have added a focused paragraph explaining that NK-cell degranulation and inflammatory cytokine production can be functionally uncoupled, and discuss the physiological and translational significance of this "de-inflammatory" state (preserved killing with restrained inflammatory output).

8. Reproducibility and data/code availability: Version-locked analysis scripts (Scanpy, Harmony, Leiden, Monocle, scMetabolic, GSEA) are now publicly available on GitHub, with the computing environment and package versions clearly listed. All sequencing and proteomics data remain accessible via CNSA (CNP0008912) and PRIDE (PXD073707).

In accordance with the editor's suggestions, we have added relevant references to support the methodological statement that single-cell approaches help resolve immune heterogeneity and state transitions (Page 4, Lines 29–31). We have also ensured that all URLs are cited as references and that the manuscript conforms to the journal's style guidelines.

We believe the revised manuscript is now significantly strengthened and hope it will be suitable for publication in *GigaScience*. We thank you again for your time and consideration.

Sincerely,

Xuan DONG

BGI Research, Hangzhou

Zhenzhong Road, Xihu District, Hangzhou, China 310030

+86-15302791017

dongxuan@genomics.cn

Reviewer #1:

1. Please clarify whether, in your scRNA-seq analysis workflow, you removed non-NK lineages (e.g., T cells) prior to downstream analyses (e.g., integration, clustering, differential expression analysis, and trajectory inference). If so, please add the corresponding procedures and rationale to the Methods section.

We sincerely thank the reviewer for this important point. Prior to performing NK-focused downstream analyses (integration, clustering, differential expression, and trajectory inference), we applied a stringent lineage and quality filtering strategy to minimize lineage-driven confounding and low-quality cell interference. First, major immune lineages were annotated using canonical marker sets (T cell markers: *CD3D, CD3E, CD4, IL7R, CD8A, CD8B*; B cell markers: *CD79A, MS4A1*; NK cell markers: *NCAM1, KLRD1, FCGR3A, GNLY, NKG7*). Non-NK-lineage cells were removed prior to constructing the NK-only dataset. In addition, we excluded low-quality or transcriptionally ambiguous clusters that lacked defining lineage markers and exhibited poor marker specificity, as these likely represented low-quality cells, multiplets, or transcriptionally uninformative populations. The resulting curated NK-only dataset was then used for integration, clustering, differential expression analysis, and trajectory inference. We have clarified this filtering strategy in both the Analyses and Methods sections to improve transparency and reproducibility.

We have clarified this filtering strategy in the Analyses section (Page 6, Lines 98–102) and provided the corresponding details in the Methods (Page 15, Lines 350–355).

2. After generating the NK-only dataset, please specify whether you recomputed the neighborhood graph and re-ran UMAP/Leiden clustering, and whether you re-ran trajectory inference on the NK-only subset (rather than reusing results obtained from the full-cell dataset).
We thank the reviewer for pointing out this important issue. After generating the NK-only subset, we re-performed batch correction and rebuilt the kNN neighborhood graph. We then re-ran UMAP embedding and Leiden clustering *de novo* on the NK-only dataset, and only thereafter proceeded with NK subset annotation and downstream comparative analyses between the MSC-Exos–treated and untreated groups. These steps have now been explicitly described in the revised Methods section. These steps have now been explicitly described in the revised Methods section (Page 15, Lines 355—359).

3. Please provide stepwise cell counts for each donor×condition before and after filtering, including at minimum: (i) total cells after QC, (ii) number of cells removed (non-NK lineages), and (iii) final number of cells retained for NK-only analyses.
We have added a new supplementary table reporting stepwise cell counts per donor × condition, including: (i) Total cells after QC, (ii) Removed cells (non-NK lineages), and (iii) Retained

cells (NK-only) for downstream analyses. We also provide an overview of cell-type composition to facilitate evaluation of potential composition shifts. We have added Supplementary Table 4 (donor-by-condition cell counts pre- and post-filtering) and clarified the lineage/quality filtering procedure in the Analyses section (Page 6, Lines 98–102) (Supplementary Table 4).

4. The decrease in IFNG mRNA (Fig. 3B) and IFN-γ protein (Suppl. Fig. 3A) appears at odds with enhanced effector phenotypes. Please add a focused Discussion explaining that NK cytotoxic degranulation and inflammatory cytokine production can be partially uncoupled (functional skewing/uncoupling), and discuss the physiological and translational significance of this 'de-inflammatory' state (e.g., preserved killing with restrained inflammatory output).
We sincerely thank the reviewer for their insightful comment. We agree that the reduction in IFNG mRNA and IFN-γ protein may appear inconsistent with enhanced effector phenotypes. We have now added a focused paragraph in the Discussion explaining that NK cell cytotoxic degranulation and inflammatory cytokine production are partially regulated by distinct signaling modules and can be functionally uncoupled. We further discuss the physiological and translational implications of this functional skewing, highlighting that preserved cytotoxicity with restrained IFN-γ production may represent a de-inflammatory state that limits collateral tissue damage while maintaining anti-target activity. This clarification has been added to the Discussion section (Page 10, Lines 204–209) with supporting citations [28–30].

5. Enrichment of "FcγR-dependent phagocytosis" should be interpreted cautiously for NK cells. This signal likely reflects FcγR-associated cytoskeletal remodeling, immune-synapse organization, and trogocytosis/phagocytosis-like processes rather than canonical antibody-dependent cellular phagocytosis (ADCP). Please clarify this NK-specific meaning in Results/Discussion and add limiting statements in figure legends.
We agree and have revised the text to avoid implying canonical ADCP by NK cells. The relevant interpretation and limiting language have been added in the Analyses text (Page 9, Lines 176–180) and in the Figure 5B legend (Page 25, Lines 632–633).

6. Methods: please report key parameters (Harmony theta/iterations—state defaults if used; Leiden resolution; Monocle2/3 version and key settings; scMetabolic gene-set source and version).
Thank you for pointing this out. We agree that key single-cell analysis parameters should be explicitly reported for reproducibility. We have added these clarifications to the Methods section at the relevant subsections (Harmony/Leiden: Page 15, Lines 344–359; Monocle2/3: Page 15, Lines 362–370; scMetabolic: Page 16, Lines 372–374).

7. Provide UMAPs before/after Harmony correction (colored by donor/batch/condition) in the

Supplementary to improve transparency of batch correction.

We sincerely thank the reviewer for this valuable suggestion. To improve transparency of batch correction, we have added Supplementary Figure 4, which visualizes UMAP embeddings before and after Harmony integration:

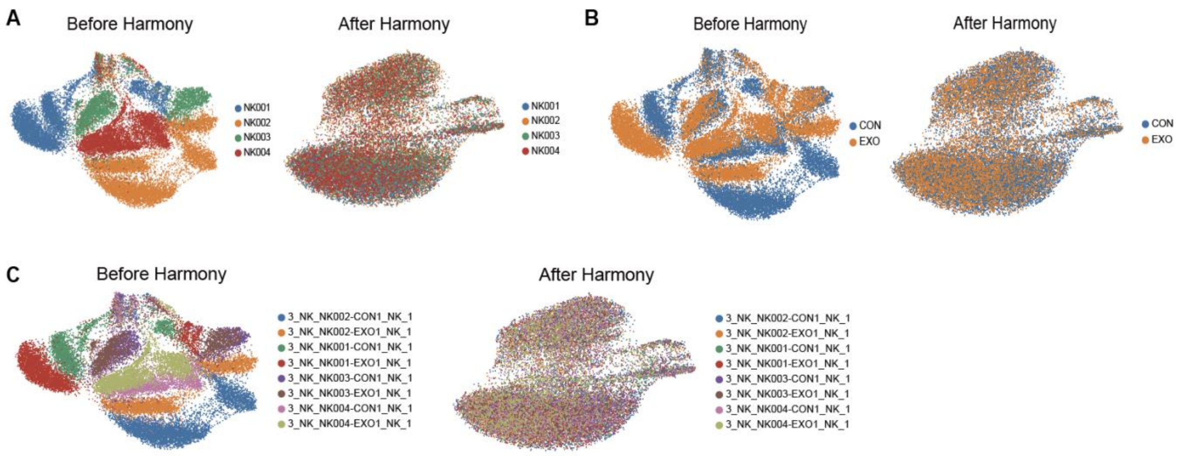

"(A) UMAP colored by donor (NK001–NK004) before and after Harmony, showing improved donor mixing after integration. (B) UMAP colored by condition (CON vs EXO) before and after Harmony, indicating that condition-associated structure remains observable after batch correction. (C) UMAP colored by batch before and after Harmony, showing reduced batch-associated separation after correction."

These clarifications have been added to the Analyses section (Page 7, Lines 134–136).

8. Add a donor×condition cell-count/composition overview plot (barplot/stacked bar) to help readers assess composition shifts.

We sincerely thank the reviewer for this valuable suggestion. To facilitate assessment of donor-by-condition composition shifts, we added (i) absolute cell counts for each donor × condition in Supplementary Table 5, and (ii) a donor-stratified compositional overview in Supplementary Figure 4D, which displays the proportions of annotated NK subclusters under CON and EXO conditions.

Added figure legend (Supplementary Fig. 4D):

D

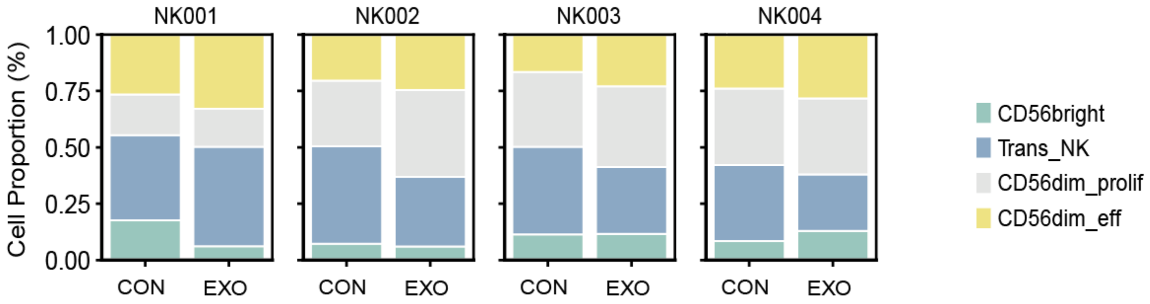

"(D) Stacked bar plots showing the proportions of annotated NK subclusters for each donor under CON and EXO treatment conditions."

We have added this clarification in the Analyses section (Page 7, Lines 138–139).

9. Share version-locked analysis scripts (Scanpy, Harmony, Leiden, Monocle, scMetabolic, GSEA) or provide a private review link; list the computing environment (Python/R and package versions) in the Supplementary.
We sincerely thank the reviewer for this insightful suggestion.

To support reproducibility, we have made the scRNA-seq analysis scripts publicly available at GitHub: https://github.com/fuyunyun-95/NK-MSC-exos-scRNAseq-analysis.git. The repository contains the complete analysis code for Scanpy-based preprocessing, Harmony integration, kNN graph reconstruction, UMAP visualization, Leiden clustering, trajectory inference (Monocle), scMetabolic scoring, and GSEA. We also provide the software environment information in the same repository, including the Python/R versions and the versions of key packages. In the Data Availability, we revised:

"The scRNA-seq analysis scripts used in this study are available at GitHub (repository: https://github.com/fuyunyun-95/NK-MSC-exos-scRNAseq-analysis.git)."

Reviewer #2:

10. In correlating exosomal proteomic findings (e.g., enrichment of FcγR signaling modules) with NK cell functional changes, certain expressions in the current manuscript may lead readers to overinterpret the certainty of causal relationships. It is recommended to systematically enhance the prudence of wording in key sections of the manuscript (abstract, conclusions, discussion)—for instance, by revising phrases to alternatives like "supports a plausible hypothesis" or "is consistent with an enhanced state."
We thank the reviewer for this important suggestion. We agree that, when integrating exosome proteomics with NK-cell functional readouts, causal relationships should not be overstated.

Accordingly, we systematically revised the wording in the Abstract, Conclusions, and Discussion to more clearly distinguish observations from hypotheses. Specifically, we replaced mechanistic/causal phrasing (e.g., “mechanistically,” “provide a mechanistic basis”) with more cautious language such as “is associated with,” “is consistent with,” “supports a plausible hypothesis,” and we added explicit limitation statements noting that the multi-omics concordance is hypothesis-generating and warrants further causal validation. These revisions are reflected in the Abstract (Page 2, Lines 40–46), Analyses (Page 9, Lines 174–176; Page 9, Lines 178–188), and Discussion (Page 9–10, Lines 192–203; Page 10, Lines 220–224).

11. Additionally, the discussion should clearly delineate direct evidence, hypotheses requiring future validation, and multiple possible interpretations, thereby precisely defining the contributions of this study and the directions for future verification.

We sincerely thank the reviewer for this valuable suggestion. We revised the Discussion to more clearly distinguish (i) conclusions directly supported by our functional and single-cell data, (ii) hypotheses suggested by multi-omics concordance that require causal validation, and (iii) alternative interpretations and future verification. Specifically, we now present the FcγR/CD16 “signaling readiness” concept as a plausible hypothesis (rather than a confirmed mechanism), explicitly note that enhanced ADCC and functional transfer/requirement of exosomal proteins were not directly demonstrated, and outline concrete validation experiments (ADCC assays, uptake/transfer and phosphorylation readouts, and BTK/PLCG2/SRC perturbation). We also discuss non-protein cargo (e.g., microRNAs) as an additional explanatory layer and highlight donor-to-donor variability as motivation to identify predictors and test durability in long-term, serial-killing, and in vivo models. These revisions are reflected in the Discussion (Page 10–11, Lines 220–235).

12. Exosome dosing should be more engineering-oriented and reproducible across batches. Reporting dose only as total protein (μg/mL) can be confounded by batch-to-batch variation in the protein-to-particle ratio. Please report in the main text and figure legends: particle concentration (particles/mL), particle-to-cell dosing (particles per cell), protein-to-particle ratio, and the rationale for SEC fraction selection.

We thank the reviewer for this insightful suggestion. To improve engineering-oriented reproducibility across preparations, we have revised the manuscript to report MSC-Exos dosing using both protein- and particle-based metrics. Specifically, we now provide (i) the particle-to-protein ratio measured by NTA, (ii) the corresponding particle concentration (particles/mL) for each protein dose, and (iii) the particle-to-cell dose (particles per cell) calculated using the dosing cell density, together with the reciprocal protein-to-particle ratio (μg per $1\times10^{\wedge 10}$ particles) (Supplementary Table 1). In addition, we clarified the rationale for SEC fraction selection: fractions were pooled from the particle-enriched peak with minimal soluble-protein

carryover (guided by a higher particle-to-protein ratio as a relative purity metric) and were subsequently validated by EV marker enrichment (CD63, TSG101, Syntenin) and depletion of the negative marker Calnexin, consistent with published SEC optimization studies and established CL-6B SEC practices.

We have added the NTA characterization and dosing/reproducibility reporting details to the Analyses section (Page 4, Lines 46–48, Lines 52–54) and Supplementary Table 1, and clarified the MSC-Exos isolation procedure (SEC) in the Methods (Page 11–12, Lines 256–259).

13. The multi-omics integration should more strictly separate pathway overlap from mechanistic delivery. Jaccard similarity and pathway overlap indicate potential concordance but do not demonstrate exosomal delivery and functional activity inside NK cells. Please harmonize phrasing across the Abstract/Conclusions/Discussion, replacing "provide a mechanistic basis/act as a delivery vehicle" with "supports a plausible hypothesis/is consistent with/suggestive of."

We thank the reviewer for this important point. This comment overlaps substantially with Reviewer Comment 10 (prudence of causal wording) and Comment 11 (clear separation of direct evidence vs hypotheses), and we have addressed these issues in a unified manner across the manuscript. Specifically, we revised the Abstract, Results/Analyses, Conclusions, and Discussion to (i) explicitly distinguish pathway overlap/Jaccard concordance from mechanistic delivery and functional activity, (ii) harmonize language to reflect the evidence strength using phrases such as “is associated with,” “is consistent with,” “supports a plausible hypothesis,” and (iii) add clear limitation statements noting that pathway overlap does not demonstrate intracellular delivery or causal necessity, which requires targeted validation. These edits are incorporated in the Abstract (Page 2, Lines 44–46), Analyses (Page 9, Lines 182–188), and Discussion (Page 10, Lines 220–228).

14. Language and terminology: refine grammar and standardize terms. For example, change line 35 "promote NK cells expansion" to "promote NK cell expansion," and line 57 "core lineage phenotypic" to "core lineage phenotype." Use a consistent term ("MSC-Exos" or "MSC-derived exosomes") and define the abbreviation at first mention.

We thank the reviewer for this helpful comment. We have thoroughly edited the manuscript to refine grammar and standardize terminology. We have updated the corresponding wording in the Analyses section (Page 4, Line 38; Page 5, Line 63–64).

15. Reproducibility details for scRNA-seq: add a supplementary table listing, per donor and condition, cell numbers pre/post filtering, Harmony parameters.

We thank the reviewer for this helpful suggestion. To improve reproducibility, we added a new supplementary table reporting stepwise cell counts per donor × condition, including (i) total cells post-QC, (ii) cells removed as non-NK lineages, and (iii) final NK-only cells retained for

downstream analyses. We also summarize donor-by-condition composition to facilitate assessment of potential composition shifts. In addition, we expanded the Methods to explicitly report the Harmony parameters and downstream graph/cluster settings used for both the full dataset and the NK-only reanalysis. These updates are reflected in Supplementary Table 4 and the corresponding text in the Analyses (Page 6, Lines 98–102) and Methods (Page 15, Lines 344–345 Page 15, Lines 355–359).

16. Strength of claims: several statements are overly strong (e.g., line177 "fundamentally differentiating"). Please soften and explicitly note where causality remains to be validated.
We thank the reviewer for this important comment. We have systematically softened the wording to better align claims with the current evidence strength and explicitly noted where causality remains to be established.

These changes are reflected in the relevant sections, including the main multi-omics interpretation framework and the statements on FcγR/CD16-related enrichment (Abstract: Page 2, Lines 44–46; Discussion: Page 9, Lines 192–196; Page 10, Lines 220–228).

17. Fig. 4H GO terms are long; consider truncating/rotating/abbreviating to improve readability.
We thank the reviewer for the suggestion. To improve readability of Fig. 4H, we have truncated overly long GO term labels (with standardized shortening) and adjusted the layout (including font size/margins) to avoid label crowding. The updated Fig. 4H is shown in the revised manuscript.

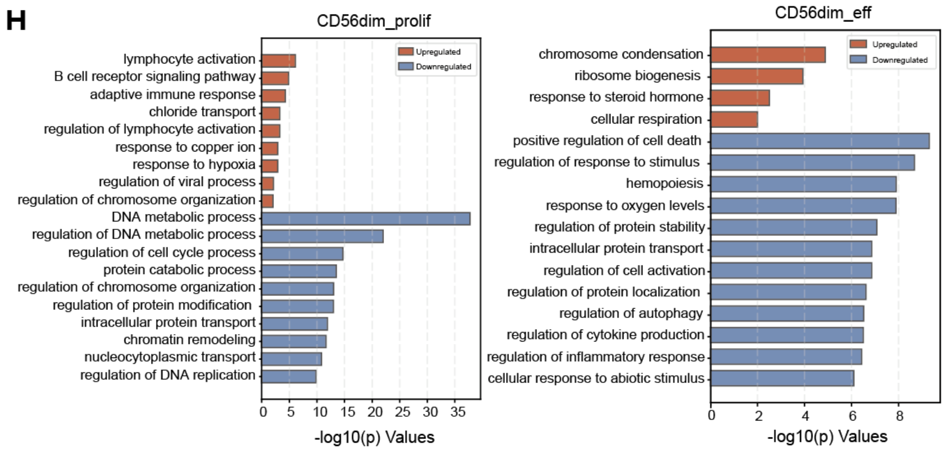

Reviewer #3:

18. Please clarify whether serum-free collection conditions were used. Detection of immunoglobulin proteins (e.g., IGHG1) in exosome proteomics may reflect serum/IgG contamination, co-isolation, or surface adsorption. Please provide appropriate controls (e.g.,

SEC fraction selection rationale) and discuss how this affects interpretation and mechanistic inferences.

We thank the reviewer for this important quality-control and interpretation comment. We have revised the manuscript to clarify serum-free collection conditions, strengthen SEC fraction-selection rationale, and temper mechanistic inferences related to immunoglobulin detection. Specifically, in the Methods, we now explicitly state that hUC-MSCs were cultured in a xeno-free, serum-free medium according to the manufacturer, and that conditioned medium for MSC-Exos isolation was therefore collected under serum-free conditions. We also expanded the SEC fraction-selection rationale, specifying that exosome-rich fractions were pooled from the particle-enriched peak with minimal soluble-protein carryover, operationalized by a higher particle-to-protein ratio as a relative purity metric and further validated by MSC-Exos marker enrichment (CD63/TSG101/Syntenin) and depletion of Calnexin. In the Results/Discussion, we revised the interpretation of immunoglobulin-related proteins (e.g., IGHG1) to note that their detection may reflect co-isolation or surface adsorption rather than serum IgG carryover, and we avoid using IGHG1 as standalone evidence for functional cargo delivery. Consistent with this, we harmonized multi-omics language to emphasize that pathway overlap/proteomic enrichment is hypothesis-generating and does not demonstrate intracellular delivery or functional activity without targeted validation. These updates are reflected at the relevant locations in the manuscript (Methods: Page 11, Lines 244–247; Page 11–12, Lines 256–258; Analyses: Page 9, Lines 178–181).

19. Although the single-cell analysis is rich, some sections read as descriptive and could better connect to functional assays. Please add cross-level synthesis statements in the Fig. 3-4 Results/Discussion (e.g., link increased CD56dim_eff frequency with killing/degranulation; connect metabolic pathway shifts with mitochondrial readouts such as membrane potential, ROS, or respiration).

We thank the reviewer for this constructive suggestion. We agree that the single-cell results should be more explicitly integrated with functional and metabolic readouts. Accordingly, we revised the Fig. 2–4 Results/Discussion to add cross-level synthesis statements linking (i) scRNA-seq–defined NK states and composition shifts to cytotoxicity/degranulation assays and (ii) transcriptomic metabolic programs to mitochondrial measurements. These revisions are reflected in the Analyses (Page 7, Lines 117–120; Page 7, Lines 140–141).

20. NK cells are not professional phagocytes; therefore, enrichment of "FcγR-dependent phagocytosis" should be interpreted cautiously. The data are more consistent with FcγR-associated cytoskeletal remodeling, immune-synapse organization, and trogocytosis/phagocytosis-like processes rather than canonical phagocytosis. Please clarify this distinction in Analyses and Discussion.

We agree and have revised the text to avoid implying canonical ADCP by NK cells. We added limiting language in Results and in the corresponding figure legends. We have clarified the interpretation of the Reactome “FcγR-dependent phagocytosis” annotation in the Analyses

(Page 8, Lines 168–172) and added corresponding limiting language in the Figure 5B legend (Page 25, Lines 632–633).

21. Potential mismatch in Supplementary figure citations: the statement "IFN-γ protein levels were also lower (Supplementary Fig. 2A)" appears inconsistent with the use of Supplementary Fig. 2A for DOX dose-response. Please verify and harmonize supplementary figure numbering.
Thank you for pointing out the potential mismatch in the Supplementary Figure citations. We have carefully checked and harmonized the supplementary figure numbering throughout the manuscript. The citation associated with IFN-γ protein levels has been corrected from Supplementary Fig. 2A to Supplementary Fig. 3A. The revised sentence now reads (Page 6, Lines 105): "Concomitantly, IFN-γ protein levels were also lower (Supplementary Fig. 3A)."

22. If exosome dose is reported as total protein (μg/mL), please also report particle concentration (particles/mL) or at least the average particle-to-protein ratio to facilitate cross-study comparison and reproducibility.
We thank the reviewer for this helpful suggestion. To facilitate cross-study comparison and improve reproducibility, we revised the manuscript to report MSC-Exos dosing using both protein- and particle-based metrics. Specifically, we now provide the particle-to-protein ratio measured by NTA ($1.9\times10^{\wedge 8}$ particles/μg), and we report the corresponding particle concentration (particles/mL) for each protein dose; we also include the reciprocal protein-to-particle ratio (52.63 μg per $1\times10^{\wedge 10}$ particles) in Supplementary Table 1. We have also added the details to the Analyses (Page 4, Lines 46–48, Lines 52–54).

23. Specify whether n denotes biological replicates (donors) vs technical replicates, paired vs unpaired design, SD vs SEM, and ensure consistency between legends and Methods/text.
We thank the reviewer for this important suggestion. We have thoroughly revised the figure legends and the Methods to explicitly define (i) whether n refers to biological replicates (donors) or technical replicates, (ii) whether comparisons were performed using paired or unpaired designs, and (iii) whether data are presented as mean ± SD or mean ± SEM. We ensured consistency of the statistical descriptions across the figure legends, Methods, and main text, and updated the relevant legends accordingly (Figure 1–4 legends; Supplementary Figure 1–3 legends).

24. Supplementary Fig. 2B: consider adding quantification for β-gal staining.
We sincerely thank the reviewer for this valuable suggestion. We have added figure legend (Supplementary Fig. 2B):

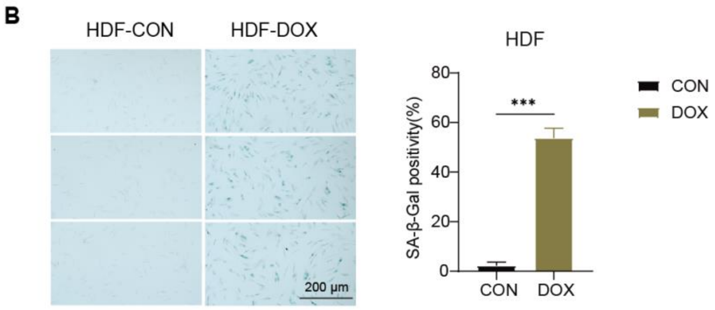

"(B) Representative bright-field images showing morphological changes and senescence-associated β-galactosidase (SA-β-Gal) staining in human dermal fibroblasts (HDFs). HDF-CON indicates untreated control cells, and HDF-DOX represents cells treated with 150 nM doxorubicin (DOX) for 48 h. Quantification of SA-β-Gal positive cells is shown on the right. Scale bar, 200 μm."

These revisions are reflected in the Analyses (Page 5, Lines 72–74).
