# Supplementary material for: Integrative single-cell transcriptomics and proteomics reveal an immunometabolic framework for MSC-exosome-mediated remodeling of expanded NK cells
Source: Gigascience. 2026 Apr 20;15:giag049. doi: 10.1093/gigascience/giag049 (PMC13273429; doi:10.1093/gigascience/giag049)
Supplement: giag049_Authors_Response_To_Reviewer_Comments_original_submission [file giag049_Authors_Response_To_Reviewer_Comments_original_submission.pdf]

Author's Response To Reviewer Comments

Reviewer #1:
1. Please clarify whether, in your scRNA-seq analysis workflow, you removed non-NK lineages (e.g., T cells) prior to downstream analyses (e.g., integration, clustering, differential expression analysis, and trajectory inference). If so, please add the corresponding procedures and rationale to the Methods section.
We sincerely thank the reviewer for this important point. Prior to performing NK-focused downstream analyses (integration, clustering, differential expression, and trajectory inference), we applied a stringent lineage and quality filtering strategy to minimize lineage-driven confounding and low-quality cell interference. First, major immune lineages were annotated using canonical marker sets (T cell markers: CD3D, CD3E, CD4, IL7R, CD8A, CD8B; B cell markers: CD79A, MS4A1; NK cell markers: NCAM1, KLRD1, FCGR3A, GNLY, NKG7). Non-NK-lineage cells were removed prior to constructing the NK-only dataset. In addition, we excluded low-quality or transcriptionally ambiguous clusters that lacked defining lineage markers and exhibited poor marker specificity, as these likely represented low-quality cells, multiplets, or transcriptionally uninformative populations. The resulting curated NK-only dataset was then used for integration, clustering, differential expression analysis, and trajectory inference. We have clarified this filtering strategy in both the Analyses and Methods sections to improve transparency and reproducibility. We have clarified this filtering strategy in the Analyses section (Page 6, Lines 98–102) and provided the corresponding details in the Methods (Page 15, Lines 350–355).
2. After generating the NK-only dataset, please specify whether you recomputed the neighborhood graph and re-ran UMAP/Leiden clustering, and whether you re-ran trajectory inference on the NK-only subset (rather than reusing results obtained from the full-cell dataset).
We thank the reviewer for pointing out this important issue. After generating the NK-only subset, we re-performed batch correction and rebuilt the kNN neighborhood graph. We then re-ran UMAP embedding and Leiden clustering de novo on the NK-only dataset, and only thereafter proceeded with NK subset annotation and downstream comparative analyses between the MSC-Exos–treated and untreated groups. These steps have now been explicitly described in the revised Methods section. These steps have now been explicitly described in the revised Methods section (Page 15, Lines 355-–359).
3. Please provide stepwise cell counts for each donor×condition before and after filtering, including at minimum: (i) total cells after QC, (ii) number of cells removed (non-NK lineages), and (iii) final number of cells retained for NK-only analyses.
We have added a new supplementary table reporting stepwise cell counts per donor × condition, including: (i) Total cells after QC, (ii) Removed cells (non-NK lineages), and (iii) Retained cells (NK-only) for downstream analyses. We also provide an overview of cell-type composition to facilitate evaluation of potential composition shifts. We have added Supplementary Table 4 (donor-by-condition cell counts pre- and post-filtering) and clarified the lineage/quality filtering procedure in the Analyses section (Page 6, Lines 98–102) (Supplementary Table 4).
4. The decrease in IFNG mRNA (Fig. 3B) and IFN-γ protein (Suppl. Fig. 3A) appears at odds with enhanced effector phenotypes. Please add a focused Discussion explaining that NK cytotoxic degranulation and inflammatory cytokine production can be partially uncoupled (functional skewing/uncoupling), and discuss the physiological and translational significance of this 'de-inflammatory' state (e.g., preserved killing with restrained inflammatory output).
We sincerely thank the reviewer for their insightful comment. We agree that the reduction in IFNG mRNA and IFN-γ protein may appear inconsistent with enhanced effector phenotypes. We have now added a focused paragraph in the Discussion explaining that NK cell cytotoxic degranulation and inflammatory cytokine production are partially regulated by distinct signaling modules and can be functionally uncoupled. We further discuss the physiological and translational implications of this functional skewing, highlighting that preserved cytotoxicity with restrained IFN-γ production may represent a de-inflammatory state that limits collateral tissue damage while maintaining anti-target activity. This clarification has been added to the Discussion section (Page 10, Lines 204–209) with supporting citations [28–30].
5. Enrichment of "FcγR-dependent phagocytosis" should be interpreted cautiously for NK cells. This signal likely reflects FcγR-associated cytoskeletal remodeling, immune-synapse organization, and trogocytosis/phagocytosis-like processes rather than canonical antibody-dependent cellular phagocytosis (ADCP). Please clarify this NK-specific meaning in Results/Discussion and add limiting statements in figure legends.

We agree and have revised the text to avoid implying canonical ADCP by NK cells. The relevant interpretation and limiting language have been added in the Analyses text (Page 9, Lines 176–180) and in the Figure 5B legend (Page 25, Lines 632–633).
6. Methods: please report key parameters (Harmony theta/iterations—state defaults if used; Leiden resolution; Monocle2/3 version and key settings; scMetabolic gene-set source and version).
Thank you for pointing this out. We agree that key single-cell analysis parameters should be explicitly reported for reproducibility. We have added these clarifications to the Methods section at the relevant subsections (Harmony/Leiden: Page 15, Lines 344–359; Monocle2/3: Page 15, Lines 362–370; scMetabolic: Page 16, Lines 372–374).
7. Provide UMAPs before/after Harmony correction (colored by donor/batch/condition) in the Supplementary to improve transparency of batch correction.
We sincerely thank the reviewer for this valuable suggestion. To improve transparency of batch correction, we have added Supplementary Figure 4, which visualizes UMAP embeddings before and after Harmony integration:

"(A) UMAP colored by donor (NK001–NK004) before and after Harmony, showing improved donor mixing after integration. (B) UMAP colored by condition (CON vs EXO) before and after Harmony, indicating that condition-associated structure remains observable after batch correction. (C) UMAP colored by batch before and after Harmony, showing reduced batch-associated separation after correction."
These clarifications have been added to the Analyses section (Page 7, Lines 134–136).
8. Add a donor×condition cell-count/composition overview plot (barplot/stacked bar) to help readers assess composition shifts.
We sincerely thank the reviewer for this valuable suggestion. To facilitate assessment of donor-by-condition composition shifts, we added (i) absolute cell counts for each donor × condition in Supplementary Table 5, and (ii) a donor-stratified compositional overview in Supplementary Figure 4D, which displays the proportions of annotated NK subclusters under CON and EXO conditions.
Added figure legend (Supplementary Fig. 4D):

"(D) Stacked bar plots showing the proportions of annotated NK subclusters for each donor under CON and EXO treatment conditions."
We have added this clarification in the Analyses section (Page 7, Lines 138–139).
9. Share version-locked analysis scripts (Scanpy, Harmony, Leiden, Monocle, scMetabolic, GSEA) or provide a private review link; list the computing environment (Python/R and package versions) in the Supplementary.
We sincerely thank the reviewer for this insightful suggestion.
To support reproducibility, we have made the scRNA-seq analysis scripts publicly available at GitHub: https://github.com/fuyunyun-95/NK-MSC-exos-scRNAseq-analysis.git. The repository contains the complete analysis code for Scanpy-based preprocessing, Harmony integration, kNN graph reconstruction, UMAP visualization, Leiden clustering, trajectory inference (Monocle), scMetabolic scoring, and GSEA. We also provide the software environment information in the same repository, including the Python/R versions and the versions of key packages. In the Data Availability, we revised:
"The scRNA-seq analysis scripts used in this study are available at GitHub (repository: https://github.com/fuyunyun-95/NK-MSC-exos-scRNAseq-analysis.git)."

Reviewer #2:
10. In correlating exosomal proteomic findings (e.g., enrichment of FcγR signaling modules) with NK cell functional changes, certain expressions in the current manuscript may lead readers to overinterpret the certainty of causal relationships. It is recommended to systematically enhance the prudence of wording in key sections of the manuscript (abstract, conclusions, discussion)—for instance, by revising phrases to alternatives like "supports a plausible hypothesis" or "is consistent with an enhanced state."
We thank the reviewer for this important suggestion. We agree that, when integrating exosome proteomics with NK-cell functional readouts, causal relationships should not be overstated. Accordingly, we systematically revised the wording in the Abstract, Conclusions, and Discussion to more clearly distinguish observations from hypotheses. Specifically, we replaced mechanistic/causal phrasing (e.g., "mechanistically," "provide a mechanistic basis") with more cautious language such as "is associated with," "is consistent with," "supports a plausible hypothesis," and we added explicit limitation statements noting that the multi-omics concordance is hypothesis-generating and warrants further causal validation. These

revisions are reflected in the Abstract (Page 2, Lines 40–46), Analyses (Page 9, Lines 174–176; Page 9, Lines 178–188), and Discussion (Page 9–10, Lines 192–203; Page 10, Lines 220–224).

11. Additionally, the discussion should clearly delineate direct evidence, hypotheses requiring future validation, and multiple possible interpretations, thereby precisely defining the contributions of this study and the directions for future verification.

We sincerely thank the reviewer for this valuable suggestion. We revised the Discussion to more clearly distinguish (i) conclusions directly supported by our functional and single-cell data, (ii) hypotheses suggested by multi-omics concordance that require causal validation, and (iii) alternative interpretations and future verification. Specifically, we now present the FcγR/CD16 "signaling readiness" concept as a plausible hypothesis (rather than a confirmed mechanism), explicitly note that enhanced ADCC and functional transfer/requirement of exosomal proteins were not directly demonstrated, and outline concrete validation experiments (ADCC assays, uptake/transfer and phosphorylation readouts, and BTK/PLCG2/SRC perturbation). We also discuss non-protein cargo (e.g., microRNAs) as an additional explanatory layer and highlight donor-to-donor variability as motivation to identify predictors and test durability in long-term, serial-killing, and in vivo models. These revisions are reflected in the Discussion (Page 10–11, Lines 220–235).

12. Exosome dosing should be more engineering-oriented and reproducible across batches. Reporting dose only as total protein (μg/mL) can be confounded by batch-to-batch variation in the protein-to-particle ratio. Please report in the main text and figure legends: particle concentration (particles/mL), particle-to-cell dosing (particles per cell), protein-to-particle ratio, and the rationale for SEC fraction selection.

We thank the reviewer for this insightful suggestion. To improve engineering-oriented reproducibility across preparations, we have revised the manuscript to report MSC-Exos dosing using both protein- and particle-based metrics. Specifically, we now provide (i) the particle-to-protein ratio measured by NTA, (ii) the corresponding particle concentration (particles/mL) for each protein dose, and (iii) the particle-to-cell dose (particles per cell) calculated using the dosing cell density, together with the reciprocal protein-to-particle ratio (μg per 1×10^10 particles) (Supplementary Table 1). In addition, we clarified the rationale for SEC fraction selection: fractions were pooled from the particle-enriched peak with minimal soluble-protein carryover (guided by a higher particle-to-protein ratio as a relative purity metric) and were subsequently validated by EV marker enrichment (CD63, TSG101, Syntenin) and depletion of the negative marker Calnexin, consistent with published SEC optimization studies and established CL-6B SEC practices.

We have added the NTA characterization and dosing/reproducibility reporting details to the Analyses section (Page 4, Lines 46–48, Lines 52–54) and Supplementary Table 1, and clarified the MSC-Exos isolation procedure (SEC) in the Methods (Page 11–12, Lines 256–259).

13. The multi-omics integration should more strictly separate pathway overlap from mechanistic delivery. Jaccard similarity and pathway overlap indicate potential concordance but do not demonstrate exosomal delivery and functional activity inside NK cells. Please harmonize phrasing across the Abstract/Conclusions/Discussion, replacing "provide a mechanistic basis/act as a delivery vehicle" with "supports a plausible hypothesis/is consistent with/suggestive of."

We thank the reviewer for this important point. This comment overlaps substantially with Reviewer Comment 10 (prudence of causal wording) and Comment 11 (clear separation of direct evidence vs hypotheses), and we have addressed these issues in a unified manner across the manuscript. Specifically, we revised the Abstract, Results/Analyses, Conclusions, and Discussion to (i) explicitly distinguish pathway overlap/Jaccard concordance from mechanistic delivery and functional activity, (ii) harmonize language to reflect the evidence strength using phrases such as "is associated with," "is consistent with," "supports a plausible hypothesis," and (iii) add clear limitation statements noting that pathway overlap does not demonstrate intracellular delivery or causal necessity, which requires targeted validation. These edits are incorporated in the Abstract (Page 2, Lines 44–46), Analyses (Page 9, Lines 182–188), and Discussion (Page 10, Lines 220–228).

14. Language and terminology: refine grammar and standardize terms. For example, change line 35 "promote NK cells expansion" to "promote NK cell expansion," and line 57 "core lineage phenotypic" to "core lineage phenotype." Use a consistent term ("MSC-Exos" or "MSC-derived exosomes") and define the abbreviation at first mention.

We thank the reviewer for this helpful comment. We have thoroughly edited the manuscript to refine grammar and standardize terminology. We have updated the corresponding wording in the Analyses section (Page 4, Line 38; Page 5, Line 63–64).

15. Reproducibility details for scRNA-seq: add a supplementary table listing, per donor and condition, cell numbers pre/post filtering, Harmony parameters.

We thank the reviewer for this helpful suggestion. To improve reproducibility, we added a new supplementary table reporting stepwise cell counts per donor × condition, including (i) total cells post-QC, (ii) cells removed as non-NK lineages, and (iii) final NK-only cells retained for downstream analyses. We also summarize donor-by-condition composition to facilitate assessment of potential composition shifts. In addition, we expanded the Methods to explicitly report the Harmony parameters and downstream graph/cluster settings used for both the full dataset and the NK-only reanalysis. These updates are reflected in Supplementary Table 4 and the corresponding text in the Analyses (Page 6, Lines 98–102) and Methods (Page 15, Lines 344–345 Page 15, Lines 355–359).

16. Strength of claims: several statements are overly strong (e.g., line177 "fundamentally differentiating"). Please soften and explicitly note where causality remains to be validated.

We thank the reviewer for this important comment. We have systematically softened the wording to better align claims with the current evidence strength and explicitly noted where causality remains to be established.

These changes are reflected in the relevant sections, including the main multi-omics interpretation framework and the statements on FcγR/CD16-related enrichment (Abstract: Page 2, Lines 44–46; Discussion: Page 9, Lines 192–196; Page 10, Lines 220–228).

17. Fig. 4H GO terms are long; consider truncating/rotating/abbreviating to improve readability.

We thank the reviewer for the suggestion. To improve readability of Fig. 4H, we have truncated overly long GO term labels (with standardized shortening) and adjusted the layout (including font size/margins) to avoid label crowding. The updated Fig. 4H is shown in the revised manuscript.

Reviewer #3:

18. Please clarify whether serum-free collection conditions were used. Detection of immunoglobulin proteins (e.g., IGHG1) in exosome proteomics may reflect serum/IgG contamination, co-isolation, or surface adsorption. Please provide appropriate controls (e.g., SEC fraction selection rationale) and discuss how this affects interpretation and mechanistic inferences.

We thank the reviewer for this important quality-control and interpretation comment. We have revised the manuscript to clarify serum-free collection conditions, strengthen SEC fraction-selection rationale, and temper mechanistic inferences related to immunoglobulin detection. Specifically, in the Methods, we now explicitly state that hUC-MSCs were cultured in a xeno-free, serum-free medium according to the manufacturer, and that conditioned medium for MSC-Exos isolation was therefore collected under serum-free conditions. We also expanded the SEC fraction-selection rationale, specifying that exosome-rich fractions were pooled from the particle-enriched peak with minimal soluble-protein carryover, operationalized by a higher particle-to-protein ratio as a relative purity metric and further validated by MSC-Exos marker enrichment (CD63/TSG101/Syntenin) and depletion of Calnexin. In the Results/Discussion, we revised the interpretation of immunoglobulin-related proteins (e.g., IGHG1) to note that their detection may reflect co-isolation or surface adsorption rather than serum IgG carryover, and we avoid using IGHG1 as standalone evidence for functional cargo delivery. Consistent with this, we harmonized multi-omics language to emphasize that pathway overlap/proteomic enrichment is hypothesis-generating and does not demonstrate intracellular delivery or functional activity without targeted validation. These updates are reflected at the relevant locations in the manuscript (Methods: Page 11, Lines 244–247; Page 11–12, Lines 256–258; Analyses: Page 9, Lines 178–181).

19. Although the single-cell analysis is rich, some sections read as descriptive and could better connect to functional assays. Please add cross-level synthesis statements in the Fig. 3-4 Results/Discussion (e.g., link increased CD56dim_eff frequency with killing/degranulation; connect metabolic pathway shifts with mitochondrial readouts such as membrane potential, ROS, or respiration).

We thank the reviewer for this constructive suggestion. We agree that the single-cell results should be more explicitly integrated with functional and metabolic readouts. Accordingly, we revised the Fig. 2–4 Results/Discussion to add cross-level synthesis statements linking (i) scRNA-seq–defined NK states and composition shifts to cytotoxicity/degranulation assays and (ii) transcriptomic metabolic programs to mitochondrial measurements. These revisions are reflected in the Analyses (Page 7, Lines 117–120; Page 7, Lines 140–141).

20. NK cells are not professional phagocytes; therefore, enrichment of "FcγR-dependent phagocytosis" should be interpreted cautiously. The data are more consistent with FcγR-associated cytoskeletal remodeling, immune-synapse organization, and trogocytosis/phagocytosis-like processes rather than canonical phagocytosis. Please clarify this distinction in Analyses and Discussion.

We agree and have revised the text to avoid implying canonical ADCP by NK cells. We added limiting language in Results and in the corresponding figure legends. We have clarified the interpretation of the Reactome “FcγR-dependent phagocytosis” annotation in the Analyses (Page 8, Lines 168–172) and added corresponding limiting language in the Figure 5B legend (Page 25, Lines 632–633).

21. Potential mismatch in Supplementary figure citations: the statement "IFN-γ protein levels were also lower (Supplementary Fig. 2A)" appears inconsistent with the use of Supplementary Fig. 2A for DOX dose-response. Please verify and harmonize supplementary figure numbering.

Thank you for pointing out the potential mismatch in the Supplementary Figure citations. We have carefully checked and harmonized the supplementary figure numbering throughout the manuscript. The citation associated with IFN-γ protein levels has been corrected from Supplementary Fig. 2A to Supplementary Fig. 3A. The revised sentence now reads (Page 6, Lines 105): “Concomitantly, IFN-γ protein levels were also lower (Supplementary Fig. 3A).”

22. If exosome dose is reported as total protein (μg/mL), please also report particle concentration (particles/mL) or at least the average particle-to-protein ratio to facilitate cross-study comparison and reproducibility.

We thank the reviewer for this helpful suggestion. To facilitate cross-study comparison and improve reproducibility, we revised the manuscript to report MSC-Exos dosing using both protein- and particle-based metrics. Specifically, we now provide the particle-to-protein ratio measured by NTA (1.9×10^8 particles/μg), and we report the corresponding particle concentration (particles/mL) for each protein dose; we also include the reciprocal protein-to-particle ratio (52.63 μg per 1×10^10 particles) in Supplementary Table 1. We have also added the details to the Analyses (Page 4, Lines 46–48, Lines 52–54).

23. Specify whether n denotes biological replicates (donors) vs technical replicates, paired vs unpaired design, SD vs SEM, and ensure consistency between legends and Methods/text.

We thank the reviewer for this important suggestion. We have thoroughly revised the figure legends and the Methods to explicitly define (i) whether n refers to biological replicates (donors) or technical replicates, (ii) whether comparisons were performed using paired or unpaired designs, and (iii) whether data are presented as mean ± SD or mean ± SEM. We ensured consistency of the statistical descriptions across the figure legends, Methods, and main text, and updated the relevant legends accordingly (Figure 1–4 legends; Supplementary Figure 1–3 legends).

24. Supplementary Fig. 2B: consider adding quantification for β-gal staining.

We sincerely thank the reviewer for this valuable suggestion. We have added figure legend (Supplementary Fig. 2B):

“(B) Representative bright-field images showing morphological changes and senescence-associated β-galactosidase (SA-β-Gal) staining in human dermal fibroblasts (HDFs). HDF-CON indicates untreated control cells, and HDF-DOX represents cells treated with 150 nM doxorubicin (DOX) for 48 h. Quantification of SA-β-Gal positive cells is shown on the right. Scale bar, 200 μm.”

These revisions are reflected in the Analyses (Page 5, Lines 72–74).

Cookie Preference Center

We use cookies which are necessary to make our site work. We may also use additional cookies to analyze, improve and personalize our content and your digital experience. For more information, see our Cookie Policy.

You may choose not to allow some types of cookies. However, blocking some types may impact your experience of our site and the services we are able to offer. See the different category headings below to find out more or change your settings.

Allow all

Manage Consent Preferences

Strictly Necessary Cookies

Always active

These cookies are necessary for the website to function and cannot be switched off in our systems. They are usually only set in response to actions made by you which amount to a request for services, such as setting your privacy preferences, logging in or filling in forms. You can set your browser to block or alert you about these cookies, but some parts of the site will not then work.

Functional Cookies

These cookies enable the website to provide enhanced functionality and personalisation. They may be set by us or by third party providers whose services we have added to our pages. If you do not allow these cookies then some or all of these services may not function properly.

Performance Cookies

These cookies allow us to count visits and traffic sources so we can measure and improve the performance of our site. They help us to know which pages are the most and least popular and see how visitors move around the site.

Targeting Cookies

These cookies may be set through our site by our advertising partners. They may be used by those companies to build a profile of your interests and show you relevant adverts on other sites. If you do not allow these cookies, you will experience less targeted advertising.

Cookie List

Clear

Apply Cancel

Consent Leg.Interest

Confirm my choices

[illegible]
