# Supplementary material for: Integrative single-cell transcriptomics and proteomics reveal an immunometabolic framework for MSC-exosome-mediated remodeling of expanded NK cells
Source: Gigascience. 2026 Apr 20;15:giag049. doi: 10.1093/gigascience/giag049 (PMC13273429; doi:10.1093/gigascience/giag049)
Supplement: giag049_Reviewer_1_Report_original_submission — Reviewer 1 -- 2/10/2026 [file giag049_Reviewer_1_Report_original_submission.pdf]

Reviewer Report

Title: Integrative single-cell transcriptomics and proteomics reveal an immunometabolic framework for MSC-exosome-mediated remodeling of expanded NK cells

Version: Original Submission **Date:** 2/10/2026

Reviewer name: Reviewer 1

Reviewer Comments to Author:

This manuscript establishes a standardized NKâ€‘cell expansion/intervention system and integrates multidimensional functional assays with singleâ€‘cell transcriptomics and exosome proteomics to interrogate how MSCâ€‘derived exosomes (MSCâ€‘Exos) reshape expanded NK cells. The data suggest that, while preserving core lineage identity, MSCâ€‘Exos promote proliferation, enhance degranulation/killingâ€‘dominant effector output, and alleviate signatures consistent with metabolic-inflammatory stress. A key strength is the singleâ€‘cell resolution analysis of NK subset composition and trajectory shifts toward a CD56dim effector state, alongside a hypothesisâ€‘generating framework in which an NRF2-mitochondrial homeostasis axis and FcÎ³R/CD16â€‘associated signaling modules jointly support a 'highâ€‘efficiency, lowâ€‘inflammation' NK functional state. Overall, the dataset is substantial and well aligned with GigaScience's dataâ€‘driven scope; however, several claims would benefit from tighter alignment with evidence strength, emphasizing the frameworkâ€‘building nature of the study.Major issues:(1) Please clarify whether, in your scRNA-seq analysis workflow, you removed non-NK lineages (e.g., T cells) prior to downstream analyses (e.g., integration, clustering, differential expression analysis, and trajectory inference). If so, please add the corresponding procedures and rationale to the Methods section.(2) After generating the NK-only dataset, please specify whether you recomputed the neighborhood graph and re-ran UMAP/Leiden clustering, and whether you re-ran trajectory inference on the NK-only subset (rather than reusing results obtained from the full-cell dataset).(3) Please provide stepwise cell counts for each donorÃ—condition before and after filtering,including at minimum: (i) total cells after QC, (ii) number of cells removed (non-NK lineages), and (iii) final number of cells retained for NK-only analyses.(4) The decrease in IFNG mRNA (Fig. 3B) and IFNâ€‘Î³ protein (Suppl. Fig. 3A) appears at odds with enhanced effector phenotypes. Please add a focused Discussion explaining that NK cytotoxic degranulation and inflammatory cytokine production can be partially uncoupled (functional skewing/uncoupling), and discuss the physiological and translational significance of this 'deâ€‘inflammatory' state (e.g., preserved killing with restrained inflammatory output).(5) Enrichment of "FcÎ³Râ€‘dependent phagocytosis" should be interpreted cautiously for NK cells. This signal likely reflects FcÎ³Râ€‘associated cytoskeletal remodeling, immuneâ€‘synapse organization, and trogocytosis/phagocytosisâ€‘like processes rather than canonical antibodyâ€‘dependent cellular phagocytosis (ADCP). Please clarify this NKâ€‘specific meaning in Results/Discussion and add limiting statements in figure legends.Minor issues:(1) Methods: please report key parameters (Harmony theta/iterationsâ€"state defaults if used; Leiden resolution; Monocle2/3 version and key settings; scMetabolic geneâ€‘set source and version).(2) Provide UMAPs before/after Harmony correction (colored by donor/batch/condition) in the Supplementary to improve

transparency of batch correction.(3) Add a donorÃ—condition cellâ€‘count/composition overview plot (barplot/stacked bar) to help readers assess composition shifts.(4) Share versionâ€‘locked analysis scripts (Scanpy, Harmony, Leiden, Monocle, scMetabolic, GSEA) or provide a private review link; list the computing environment (Python/R and package versions) in the Supplementary.

Level of Interest

Please indicate how interesting you found the manuscript: Choose an item.

Quality of Written English

Please indicate the quality of language in the manuscript: Choose an item.

Declaration of Competing Interests

Please complete a declaration of competing interests, considering the following questions:

- Have you in the past five years received reimbursements, fees, funding, or salary from an organisation that may in any way gain or lose financially from the publication of this manuscript, either now or in the future?
- Do you hold any stocks or shares in an organisation that may in any way gain or lose financially from the publication of this manuscript, either now or in the future?
- Do you hold or are you currently applying for any patents relating to the content of the manuscript?
- Have you received reimbursements, fees, funding, or salary from an organization that holds or has applied for patents relating to the content of the manuscript?
- Do you have any other financial competing interests?
- Do you have any non-financial competing interests in relation to this paper?

If you can answer no to all of the above, write 'I declare that I have no competing interests' below. If your reply is yes to any, please give details below.

I declare that I have no competing interests

I agree to the open peer review policy of the journal. I understand that my name will be included on my report to the authors and, if the manuscript is accepted for publication, my named report including any attachments I upload will be posted on the website along with the authors' responses. I agree for my report to be made available under an Open Access Creative Commons CC-BY license (http://creativecommons.org/licenses/by/4.0/). I understand that any comments which I do not wish to be included in my named report can be included as confidential comments to the editors, which will not be published.

I agree to the open peer review policy of the journal

To further support our reviewers, we have joined with Publons, where you can gain additional credit to further highlight your hard work (see: https://publons.com/journal/530/gigascience). On publication of this paper, your review will be automatically added to Publons, you can then choose whether or not to claim your Publons credit. I understand this statement.

Yes Choose an item.
