# Supplementary material for: Integrative single-cell transcriptomics and proteomics reveal an immunometabolic framework for MSC-exosome-mediated remodeling of expanded NK cells
Source: Gigascience. 2026 Apr 20;15:giag049. doi: 10.1093/gigascience/giag049 (PMC13273429; doi:10.1093/gigascience/giag049)
Supplement: giag049_Reviewer_2_Report_original_submission — Reviewer 2 -- 2/10/2026 [file giag049_Reviewer_2_Report_original_submission.pdf]

Reviewer Report

Title: Integrative single-cell transcriptomics and proteomics reveal an immunometabolic framework for MSC-exosome-mediated remodeling of expanded NK cells

Version: Original Submission **Date:** 2/10/2026

Reviewer name: Reviewer 2

Reviewer Comments to Author:

This study systematically evaluates the impact of mesenchymal stem cell-derived exosomes (MSCâ€‘Exos) on ex vivo NKâ€‘cell expansion. The authors report that MSCâ€‘Exos promote NKâ€‘cell proliferation, enhance degranulation/killingâ€‘dominant effector output, and improve metabolic homeostasis. By integrating singleâ€‘cell transcriptomics with exosome proteomics, the manuscript proposes that MSCâ€‘Exos may confer a highâ€‘efficiency and lowâ€‘stress NK functional state through immunometabolic reprogramming and enrichment/supply of FcÎ³Râ€‘associated signaling components. From an engineering and therapeuticâ€‘translation standpoint, the work is timely and potentially impactful, with a clear multiâ€‘omics framework and an appropriate emphasis on context dependency. However, several aspects require clarification before the manuscript is considered ready for publication: stricter delineation of correlation versus causality, more cautious interpretation of proteomic 'cargo' signals.Major comments(1) In correlating exosomal proteomic findings (e.g., enrichment of FcÎ³R signaling modules) with NK cell functional changes, certain expressions in the current manuscript may lead readers to overinterpret the certainty of causal relationships. It is recommended to systematically enhance the prudence of wording in key sections of the manuscript (abstract, conclusions, discussion)â€”for instance, by revising phrases to alternatives like "supports a plausible hypothesis" or "is consistent with an enhanced state."(2) Additionally, the discussion should clearly delineate direct evidence, hypotheses requiring future validation, and multiple possible interpretations, thereby precisely defining the contributions of this study and the directions for future verification.(3) Exosome dosing should be more engineeringâ€‘oriented and reproducible across batches. Reporting dose only as total protein (Î¼g/mL) can be confounded by batchâ€‘toâ€‘batch variation in the proteinâ€‘toâ€‘particle ratio. Please report in the main text and figure legends: particle concentration (particles/mL), particleâ€‘toâ€‘cell dosing (particles per cell), proteinâ€‘toâ€‘particle ratio, and the rationale for SEC fraction selection.(4) The multiâ€‘omics integration should more strictly separate pathway overlap from mechanistic delivery. Jaccard similarity and pathway overlap indicate potential concordance but do not demonstrate exosomal delivery and functional activity inside NK cells. Please harmonize phrasing across the Abstract/Conclusions/Discussion, replacing "provide a mechanistic basis/act as a delivery vehicle" with "supports a plausible hypothesis/is consistent with/suggestive of."Minor comments(1) Language and terminology: refine grammar and standardize terms. For example, change line 35 "promote NK cells expansion" to "promote NK cell expansion," and line 57 "core lineage phenotypic" to "core lineage phenotype." Use a consistent term ("MSCâ€‘Exos" or "MSCâ€‘derived exosomes") and define the abbreviation at first mention.(2) Reproducibility details for scRNAâ€‘seq: add a supplementary table listing, per donor and condition, cell numbers pre/post filtering, Harmony parameters.(3) Strength of

claims: several statements are overly strong (e.g., line177 "fundamentally differentiating"). Please soften and explicitly note where causality remains to be validated.(4) Fig. 4H GO terms are long; consider truncating/rotating/abbreviating to improve readability.

Level of Interest

Please indicate how interesting you found the manuscript: Choose an item.

Quality of Written English

Please indicate the quality of language in the manuscript: Choose an item.

Declaration of Competing Interests

Please complete a declaration of competing interests, considering the following questions:

- Have you in the past five years received reimbursements, fees, funding, or salary from an organisation that may in any way gain or lose financially from the publication of this manuscript, either now or in the future?
- Do you hold any stocks or shares in an organisation that may in any way gain or lose financially from the publication of this manuscript, either now or in the future?
- Do you hold or are you currently applying for any patents relating to the content of the manuscript?
- Have you received reimbursements, fees, funding, or salary from an organization that holds or has applied for patents relating to the content of the manuscript?
- Do you have any other financial competing interests?
- Do you have any non-financial competing interests in relation to this paper?

If you can answer no to all of the above, write 'I declare that I have no competing interests' below. If your reply is yes to any, please give details below.

I declare that I have no competing interest.

I agree to the open peer review policy of the journal. I understand that my name will be included on my report to the authors and, if the manuscript is accepted for publication, my named report including any attachments I upload will be posted on the website along with the authors' responses. I agree for my report to be made available under an Open Access Creative Commons CC-BY license (http://creativecommons.org/licenses/by/4.0/). I understand that any comments which I do not wish to be included in my named report can be included as confidential comments to the editors, which will not be published.

I agree to the open peer review policy of the journal

To further support our reviewers, we have joined with Publons, where you can gain additional credit to further highlight your hard work (see: https://publons.com/journal/530/gigascience). On publication of this paper, your review will be automatically added to Publons, you can then choose whether or not to claim your Publons credit. I understand this statement.

Yes Choose an item.
