# Supplementary material for: Integrative single-cell transcriptomics and proteomics reveal an immunometabolic framework for MSC-exosome-mediated remodeling of expanded NK cells
Source: Gigascience. 2026 Apr 20;15:giag049. doi: 10.1093/gigascience/giag049 (PMC13273429; doi:10.1093/gigascience/giag049)
Supplement: giag049_Reviewer_3_Report_original_submission — Reviewer 3 -- 2/12/2026 [file giag049_Reviewer_3_Report_original_submission.pdf]

Reviewer Report

Title: Integrative single-cell transcriptomics and proteomics reveal an immunometabolic framework for MSC-exosome-mediated remodeling of expanded NK cells

Version: Original Submission **Date:** 2/12/2026

Reviewer name: Reviewer 3

Reviewer Comments to Author:

This study establishes a standardized ex vivo NKâ€‘cell expansion platform with MSCâ€‘Exos intervention and integrates functional assays with singleâ€‘cell transcriptomics and exosome proteomics. The data indicate that MSCâ€‘Exos promote NK proliferation, enhance degranulation/killingâ€‘dominant effector output, and are associated with reduced inflammatory cytokine signatures and improved metabolic robustness. This work is aligned with clinically relevant manufacturing settings, positioning MSCâ€‘Exos as a functional adjuvant and proposing a CD16/FcÎ³Râ€‘axis "signaling readiness/priming" hypothesis with potential pharmacological implications. Nevertheless, the manuscript would benefit from tighter causal boundaries in mechanistic claims and a more explicit discussion of synergy with existing immunotherapeutics (antibodies, NK engagers).Major comments(1) Please clarify whether serumâ€‘free collection conditions were used. Detection of immunoglobulin proteins (e.g., IGHG1) in exosome proteomics may reflect serum/IgG contamination, coâ€‘isolation, or surface adsorption. Please provide appropriate controls (e.g., SEC fraction selection rationale) and discuss how this affects interpretation and mechanistic inferences.(2) Although the singleâ€‘cell analysis is rich, some sections read as descriptive and could better connect to functional assays. Please add crossâ€‘level synthesis statements in the Fig. 3-4 Results/Discussion (e.g., link increased CD56dim_eff frequency with killing/degranulation; connect metabolic pathway shifts with mitochondrial readouts such as membrane potential, ROS, or respiration).(3) NK cells are not professional phagocytes; therefore, enrichment of "FcÎ³Râ€‘dependent phagocytosis" should be interpreted cautiously. The data are more consistent with FcÎ³Râ€‘associated cytoskeletal remodeling, immuneâ€‘synapse organization, and trogocytosis/phagocytosisâ€‘like processes rather than canonical phagocytosis. Please clarify this distinction in Analyses and Discussion.Minor comments(1) Potential mismatch in Supplementary figure citations: the statement "IFNâ€‘Î³ protein levels were also lower (Supplementary Fig. 2A)" appears inconsistent with the use of Supplementary Fig. 2A for DOX dose-response. Please verify and harmonize supplementary figure numbering.(2) If exosome dose is reported as total protein (Î¼g/mL), please also report particle concentration (particles/mL) or at least the average particleâ€‘toâ€‘protein ratio to facilitate crossâ€‘study comparison and reproducibility.(3) Specify whether n denotes biological replicates (donors) vs technical replicates, paired vs unpaired design, SD vs SEM, and ensure consistency between legends and Methods/text.(4) Supplementary Fig. 2B: consider adding quantification for Î²â€‘gal staining.

Level of Interest

Please indicate how interesting you found the manuscript: Choose an item.

Quality of Written English

Please indicate the quality of language in the manuscript: Choose an item.

Declaration of Competing Interests

Please complete a declaration of competing interests, considering the following questions:

- Have you in the past five years received reimbursements, fees, funding, or salary from an organisation that may in any way gain or lose financially from the publication of this manuscript, either now or in the future?
- Do you hold any stocks or shares in an organisation that may in any way gain or lose financially from the publication of this manuscript, either now or in the future?
- Do you hold or are you currently applying for any patents relating to the content of the manuscript?
- Have you received reimbursements, fees, funding, or salary from an organization that holds or has applied for patents relating to the content of the manuscript?
- Do you have any other financial competing interests?
- Do you have any non-financial competing interests in relation to this paper?

If you can answer no to all of the above, write 'I declare that I have no competing interests' below. If your reply is yes to any, please give details below.

I declare that I have no competing interests.

I agree to the open peer review policy of the journal. I understand that my name will be included on my report to the authors and, if the manuscript is accepted for publication, my named report including any attachments I upload will be posted on the website along with the authors' responses. I agree for my report to be made available under an Open Access Creative Commons CC-BY license (http://creativecommons.org/licenses/by/4.0/). I understand that any comments which I do not wish to be included in my named report can be included as confidential comments to the editors, which will not be published.

I agree to the open peer review policy of the journal

To further support our reviewers, we have joined with Publons, where you can gain additional credit to further highlight your hard work (see: https://publons.com/journal/530/gigascience). On publication of this paper, your review will be automatically added to Publons, you can then choose whether or not to claim your Publons credit. I understand this statement.

Yes Choose an item.
